# Recent HPLC-UV Approaches for Cannabinoid Analysis: From Extraction to Method Validation and Quantification Compliance

**DOI:** 10.3390/ph18060786

**Published:** 2025-05-24

**Authors:** Eduarda M. P. Silva, Antonella Vitiello, Agnese Miro, Carlos J. A. Ribeiro

**Affiliations:** 1 Associate Laboratory i4HB—Institute for Health and Bioeconomy, University Institute of Health Sciences—CESPU, 4585-116 Gandra, Portugal; eduarda.silva@iucs.cespu.pt; 2 UCIBIO—Applied Molecular Biosciences Unit, Translational Toxicology Research Laboratory, University Institute of Health Sciences (1H-TOXRUN, IUCS-CESPU), 4585-116 Gandra, Portugal; 3 Department of Pharmacy, University of Naples Federico II, 80131 Naples, Italy; antonella.vitiello3@unina.it (A.V.); agnese.miro@unina.it (A.M.); 4 Avextra Portugal SA, 7570-003 Grândola, Portugal

**Keywords:** cannabis, cannabinoids, extraction, HPLC, UV, validation

## Abstract

Since the 1990s, cannabis has experienced a gradual easing of access restrictions, accompanied by the expansion of its legalization and commercialization. This shift has led to the proliferation of cannabis-based products, available as cosmetics, food supplements, and pharmaceutical dosage forms. Consequently, there has been a growing demand for reliable and reproducible extraction techniques alongside precise analytical methods for detecting and quantifying cannabinoids, both of which are essential for ensuring consumer safety and product quality. Given the variability in extraction and quantification techniques across laboratories, significant attention has recently been directed toward method validation. Validated methods ensure precise cannabinoid measurement in cannabis-based products, supporting compliance with dosage guidelines and legal limits. Thus, this review highlights recent advancements in these areas, with a particular focus on High-Performance Liquid Chromatography (HPLC) coupled with Ultraviolet (UV) detection, as it is considered the gold standard for cannabinoid analysis included in cannabis monographs present in several pharmacopeias. The research focused on studies published between January 2022 and December 2024, sourced from PubMed, Scopus, and Web of Science, that employed an HPLC-UV analytical technique for the detection of phytocannabinoids. Additionally, the review examines cannabinoid extraction techniques and the validation methodologies used by the authors in the selected papers. Notably, ultrasound extraction has emerged as the most widely utilized technique across various matrices, with Deep Eutectic Solvents (DESs) offering a promising, efficient, and environmentally friendly extraction alternative. Analytical chromatographic separations continue to be predominantly conducted using C18 reversed-phase columns. Nevertheless, in recent years, researchers have explored various stationary phases, particularly to achieve the enantioseparation of cannabinoids.

## 1. Introduction

Cannabis is a versatile plant with a long history of medicinal, recreational, and industrial use, garnering significant scientific and commercial interest in recent years. Due to its pronounced therapeutic properties, it is considered a highly promising medicinal plant, despite its common use as a substance of abuse [1]. The legalization of cannabis in various regions has catalyzed an expansion of research efforts, focusing on its chemical composition, therapeutic potential, and safety profile. From a taxonomic point of view, there is no general agreement regarding the classification of this plant. However, three species of the *Cannabis* genus have been described: *Cannabis sativa* L., *Cannabis indica* Lam., and *Cannabis ruderalis* [2]. Among its many constituents, including terpenes, terpenoids, stilbenoids, lignanamides, carotenoids, flavonoids, and alkaloid compounds, the most notable class of compound in cannabis remains the phytocannabinoids [3]. In addition to these phytochemicals, terpenes are thought to interact synergistically with cannabinoids, potentially enhancing their therapeutic effects by contributing to the entourage effect [4].

Medical cannabis has swiftly gained significant importance in contemporary society, fueled by renewed interest in its use as a therapeutical tool. Two cannabinoids, Δ^9^-tetrahydrocannabinol (Δ^9^-THC) and cannabidiol (CBD), are currently available on the market in formulations approved by the Food and Drug Administration (FDA) and the European Medicines Agency (EMA) [5]. These drugs represent a significant shift in how cannabinoids are perceived and used in medical practice, representing a departure from traditional herbal preparations to standardized pharmaceutical formulations backed by scientific evidence and regulatory approval. Beyond its use as pharmaceuticals, cannabis is also becoming increasingly popular in the food supplement and cosmetics industries. The expansion of commercial cannabinoid-based products is prompting revisions of cannabis regulatory frameworks to permit their legal use [6]. At the same time, efforts are being made to curb recreational usage, as cannabis remains the most widely used illegal drug worldwide. A legal limit has been established to distinguish between drug-type and non-drug-type cannabis. In most European countries, this classification relies on the content of Δ^9^-THC, the primary psychoactive compound in cannabis. Cannabis plants with a Δ^9^-THC content of 0.2% or less are classified as hemp and are primarily cultivated for industrial uses, such as fiber production. In contrast, cannabis plants with a Δ^9^-THC content greater than 0.2% are classified as marijuana and are subject to stricter regulations due to their psychoactive properties [7].

Over the past decade, numerous review articles have been published outlining the various analytical methods available for detecting phytocannabinoids [8,9,10,11,12,13,14,15,16,17,18], with some specifically focusing on Gas Chromatography (GC) [19,20] or Liquid Chromatography (LC) [21,22,23,24]. Due to the widespread use of High-Performance Liquid Chromatography (HPLC) coupled with Ultraviolet (UV) and Diode Array Detection (DAD), which has been featured in an astonishing 169 publications from 2022 to 2024, this review will comprehensively compile information regarding this technique. Furthermore, since recent reviews often fail to provide a thorough discussion of the approaches used to validate analytical techniques, the present review will analyze key validation parameters, such as precision, accuracy, and lower limits, utilized by the authors to achieve accurate and reliable quantification of cannabinoids. In addition, this review will briefly examine, within these manuscripts, the types of extraction methods and matrices selected for HPLC–UV analysis.

## 2. Cannabinoids

**Figure 1:**
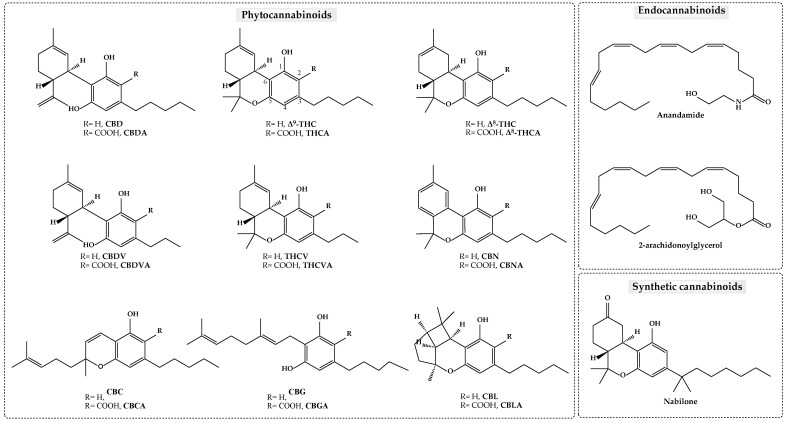
Chemical structures of key cannabinoids.

The term “cannabinoids” refers to a complex family of herbal, endogenous, and synthetic compounds that bind to specific receptors within the body’s endocannabinoid system, causing a medicinal response. These are classified into three groups: endocannabinoids, synthetic cannabinoids, and phytocannabinoids (Figure 1) [25]. Endocannabinoids play critical roles in regulating various physiological processes such as pain, mood, appetite, and memory by interacting with cannabinoid receptors (CB1 and CB2) in the endocannabinoid system [26]. Synthetic cannabinoids consist of a diverse range of chemical structures designed to interact with this endogenous system to mimic and enhance the effects of natural cannabinoids, particularly Δ^9^-THC [27]. Phytocannabinoids are primarily responsible for the plant’s medicinal and psychoactive properties. To date, more than 100 distinct phytocannabinoids have been identified from *Cannabis sativa* L. [28]. Most phytocannabinoids occur in two forms: as acids (dominant form in plants) and as neutral, decarboxylated compounds (commonly found in processed plant material). Acidic cannabinoids (AC) are thermally unstable and can undergo decarboxylation to neutral cannabinoids (NC) when subjected to light or heat (e.g., extraction at high temperatures, smoking) [29]. The two major cannabinoids in cannabis-related products are Δ^9^-THC and CBD. In this section, only phytocannabinoids with current therapeutic interest will be described.

### 2.1. Δ^9^-Tetrahydrocannabinol (Δ^9^-THC)

Among all cannabinoids, Δ^9^-THC shows the highest potency in both CB1 and CB2. It acts as a moderate partial agonist, resulting in a mixed agonist–antagonist behavior [30]. This interaction underpins many of its psychoactive effects, as well as its therapeutic properties as an analgesic, muscle relaxant, and antispasmodic agent [31]. Clinical trials have shown positive effect in treating neuropathic pain [32,33], opioid withdrawal [34,35], stress disorders [36,37], and sleep apnea [38]. Dronabinol (synthetic Δ^9^-THC) is already used in clinic for HIV/AIDS-induced loss of appetite [39] and to treat chemotherapy-induced nausea and vomiting (CINV) [40].

### 2.2. Cannabidiol (CBD)

CBD and its precursor, cannabidiolic acid (CBDA), are the most common nonpsychoactive cannabinoids found in cannabis-related products. CBD acts as an antagonist of CB1/CB2 agonists and a negative allosteric modulator of CB receptors [41,42]. Due to CBDA’s high content in certain cultivars, its non-psychoactive nature, coupled with its diverse pharmacological properties, CBD has gained significant attention in recent years, leading to a remarkable increase in the development of CBD-based product [43]. Several clinical trials involving CBD have revealed promising effects in epileptic seizures [44,45,46], Parkinson’s disease tremors [47], depression symptoms [48], anxiety [49,50], psychosis [51,52], substance use-disorder [53], and insomnia [54]. In addition, association of CBD and Δ^9^-THC has been studied in clinical trials for the management of multiple sclerosis-related spasticity [55,56], and CINV [57]. Sativex^®^ (oromucosal spray containing standardized extract with 2.7 mg Δ^9^-THC and 2.5 mg CBD per 100 µL spray) and Epidyolex^®^ (100 mg CBD per 1 mL oral solution) are already approved drugs to treat symptoms of multiple sclerosis and epilepsy, respectively [10].

### 2.3. Cannabinol (CBN)

CBN is formed by non-enzymatic oxidative aromatization of Δ^9^-THC [58]. Its levels in plant material are initially low but can increase over time as Δ^9^-THC is exposed to light, heat, and oxygen, resulting in higher concentrations of CBN in aged cannabis preparations [59]. CBN has lower binding affinities for the CB1 and CB2 receptors compared to Δ^9^-THC, which means it does not produce the same psychoactive effects. Recent clinical trial suggested that CBN may help reduce overall sleep disturbances [60] and possibly attenuate chronic itch in Epidermolysis Bullosa when applied in a cream form [61].

### 2.4. Cannabigerol (CBG)

CBG is a minor cannabinoid that displays weak partial agonist activity at both the CB1 and CB2 receptors [62]. Pre-clinical studies suggest several potential therapeutic interests, such as analgesic, anti-inflammatory [63], and antibacterial [64] effects, as well as a positive outcome in inflammatory bowel disease [65]. A patient survey revealed that consumers used CBG-dominant products to manage anxiety, depression, chronic pain, and insomnia [66], and a recent published clinical trial revealed positive effect of CBG in reducing anxiety and stress in healthy adults [67].

### 2.5. Tetrahydrocannabivarin (THCV)

THCV is a *n*-propyl analog of THC that lacks its psychoactive effects [68]. It binds to both CB1 and CB2 receptors acting as an antagonist or reverse agonist [69]. In rodent studies, THCV has been shown to reduce inflammatory pain [70], epileptic activity [71], and food intake [72]. In a clinical trial, significative weight loss was observed in subjects using THCV and CBD-infused mucoadhesive strips [73]. In addition, THCV has shown potential protective role against psychological and physiological effects induced by Δ^9^-THC in healthy volunteers [74].

### 2.6. Cannabidivarin (CBDV)

CBDV is a *n*-propyl homologue of CBD that displays low binding affinity for both CB1 and CB2 receptors [75]. CBDV has been link to intestinal anti-inflammatory [76], and anti-convulsant activity in animal models [77]. Recent human trials revealed a positive correlation between CBDV and seizure control in adults with focal seizures [78] and girls with Rett syndrome [79].

## 3. Extraction of Cannabinoids

**Figure 2:**
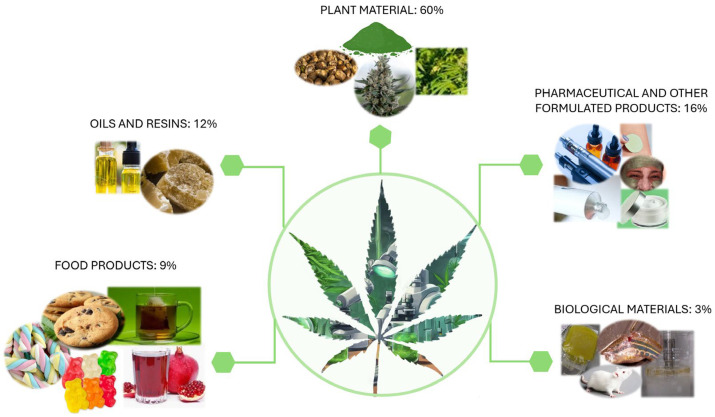
Distribution of the publications reported in Table S1, categorized by the types of matrices used and analyzed.

An overview and discussion of the extraction methods described in the manuscripts collected in Table 1 [80,81,82,83,84,85,86,87,88,89,90,91,92,93,94,95,96,97,98,99,100,101,102,103,104,105,106,107,108,109,110,111,112,113,114,115,116,117,118,119,120,121,122,123,124,125,126,127,128,129,130,131,132,133,134,135,136,137,138,139,140,141,142,143,144,145,146,147,148,149,150,151,152,153,154,155,156,157,158,159,160,161,162], Table 2 [163,164,165,166,167,168,169,170,171,172,173,174,175,176,177,178,179,180,181,182,183,184,185,186,187,188,189,190,191,192,193,194,195,196,197,198,199,200,201,202,203,204,205,206,207,208,209,210,211,212,213,214,215,216], Table S2 [217,218,219,220,221,222,223,224,225,226,227,228], and Table S3 [109,223,229,230,231,232,233,234,235,236,237,238,239,240,241,242,243,244,245,246,247,248,249,250,251], concerning original manuscripts published between 2022 and 2024, will be presented herein. Most of the studies are focused on the extraction of cannabinoids from plant materials (Figure 2), namely, inflorescence, leaves, and stems (Table S1, entries SE1–97). Nineteen manuscripts were published regarding the extraction of cannabinoids from resins and oils, natural and commercially obtained (entries SE98–116), while twenty-six considered pharmaceutical and other formulated products (entries SE132–157). Several research groups focused their research efforts on the extraction of cannabinoids incorporated into food, beverages, and tablets (entries SE117–131). Finally, a smaller number of publications considered the extraction of cannabinoids from biological matrices such as plasma and tissues (entries SE158–162). It is important to note that the authors do not intend to revise all extraction techniques applied to the analysis of cannabinoids, but rather to discuss those applied in recent research, considering the manuscripts collected in Table S1. For more detailed reviews on the topic, a considerable number have been published recently, namely by Sainz Martinez et al. [252], Al Ubeed et al. [253], Liu et al. [254], Nahar et al. [255], and Szijj [256].

### 3.1. Extraction of Cannabinoids from Plant Material

**Figure 3:**
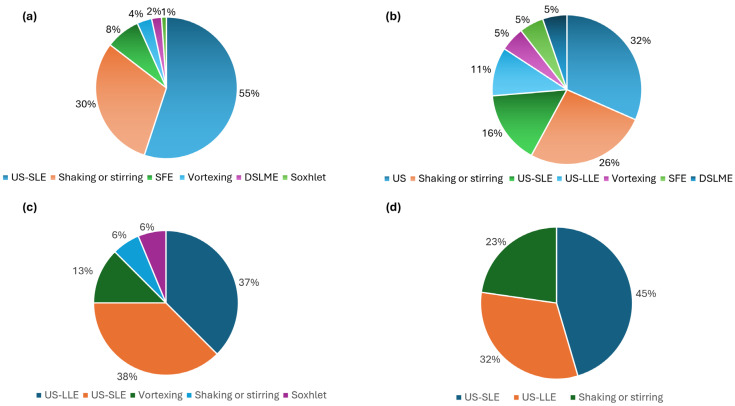
(**a**) Distribution of the reported extraction techniques based on the matrices employed: (**a**) Plant materials; (**b**) Oils and resins; (**c**) Food products; (**d**) Pharmaceutical and other formulated products. DSLME: dispersive solid–liquid microextraction; US: ultrasound; US-LLE: ultrasound liquid–liquid extraction; US-SLE: ultrasound solid–liquid extraction; SFE: supercritical fluid extraction (scCO_2_).

The flowering parts of the female cannabis plant (Table S1, entries SE1–70) are the most frequently used for cannabinoid extraction, as they contain the highest concentrations of cannabinoids [257,258]. Analysis of Table S1 indicate that solid–liquid extraction (SLE) is the most common technique used for extracting phytocannabinoids from plant material. To assist with this extraction methodology and increase the yield of extracted cannabinoids, various physical techniques are usually employed (Figure 3a). Dynamic maceration (DM), due to its simplicity, is an often-used procedure, normally accompanied by mechanical or manual stirring (entries SE10, SE11, SE13, SE45, SE47, SE50–52, SE54, SE67, and SE87) or shaking (entries SE1–7, SE9, SE24, SE26, SE52, SE65, SE71, SE73, SE76, and SE82). Vortexing is frequently used simply to allow thorough mixing between solvent and plant material; only in three manuscripts is vortexing used for extraction purposes (entries SE25, SE68, and SE83). Shaking-assisted extraction usually involves a broader and slower movement, often in a linear or circular fashion, accommodating larger volumes, making it more suitable for scaled-up extractions of up to 100 g of plant material and 500 mL of extraction solvent [83,125,183]. In comparison, vortexing creates a higher-speed circular motion, usually in small containers, suited only for small-batch extractions of around 100 mg of plant material [114,221]. In contrast to the simplicity of DM, supercritical fluid extraction (SFE) requires high-pressure solvents, especially supercritical CO_2_ (scCO_2_) and therefore entails specific automated equipment [259]. SFE is commonly used for cannabis extraction-separation in batch processes (Figure 3a) and is considered efficient for extracting both neutral and acidic cannabinoids, as it does not induce the degradation of acidic cannabinoid. Moreover, in SFE the co-extraction of undesirable compounds, like chlorophyll, is minimized [252,260,261]. SFE has recently been used to extract cannabinoids from ground plant material, namely inflorescences, leaves, and stalks, using scCO_2_ (entries SE48–51, SE53, SE67, and SE95). Fernández and co-workers [115] (entry SE50), performed the extraction of THC and CBD from two different cannabis inflorescences chemotypes containing high total THC and high total CBD content. Their strategy involved the addition of small amounts of ethanol (EtOH) as co-solvent to address the low polarity of scCO_2_, thereby enhancing the extraction process and increasing the total THC and CBD recovery. To optimize the extraction of the cannabinoids of interest, the authors also studied extraction temperature and pressure and performed a decarboxylation process. For the THC-dominant chemotype, the best extraction conditions were 70 °C, 300 bar using 10% of EtOH. For the CBD-rich chemotype, which is best extracted at lower temperatures, the optimum extraction conditions were 60 °C, 300 bar using 10% of EtOH. Furthermore, higher yields were obtained for both chemotypes using scCO_2_ extraction compared with EtOH DM at room temperature. Mileti et al. [221] (entry SE67) investigated various extraction techniques, including Soxhlet extraction (SE) and DM using EtOH as solvent, and compared their performance against SFE using scCO_2_. Although a direct comparison of these techniques is challenging due to significant differences in sample amount, temperature, time, and solvent volume (SE: 15 ± 1 g, 400 mL EtOH, 78 °C, 8 h; DM: 1 g, 100 mL EtOH (total volume), RT, 45 min (15 min each extraction); SFE: 25 ± 1 g, 40 °C, 140 min), the authors concluded that, under these specific conditions, SE yielded the highest amount of extracted material. Regarding CBD yield, DM performed best. They also noted the importance of considering cannabinoid degradation during longer extraction times and at higher temperatures, particularly with techniques like Soxhlet. SFE did not lead to higher cannabinoid yields, likely due to the non-polar nature of the supercritical fluid. It would have been important to study the use of a co-solvent, such as EtOH, as previously described, and to investigate whether comparable extraction times and temperatures across the different techniques would improve cannabinoid yield.

Mastellone et al. [93] (entry SE94) explored the use of natural eutectic solvents (ESs) in a dispersive solid–liquid microextraction (DSLME) method, complemented with sonication, for the extraction of flavonoids and cannabinoids from the aerial parts, mainly stem and leaves, of the cannabis plant. By integrating ultrasound with liquid–liquid microextraction, US-LLME enhances analyte transfer between immiscible phases while reducing the amount of solvent needed to enrich the final extract with analyte. The ESs used in the extraction process were prepared as a 1:1 mixture of hydrogen bond donor (HBD) and acceptor (HBA) natural compounds, namely menthol, linalool, thymol, carvacrol, terpinene-4-ol, and eugenol. Two years later, the group applied this methodology using different ESs (entry SE66), including an ammonium bromide salt (dimethyl-(2-hydroxy)ethyl-hexadecyl ammonium bromide) as the HBA and thymol as the HBD, as well as one based on natural terpenoids (menthol:linalool, 1:1) [220]. This DSLME ultrasound-assisted extraction (UAE) required only 100 μL of ESs and 2 mL of water as co-solvent per 100 mg of plant material (entry SE66). According to the authors, the ESs combining hydrophobic properties and hydrophilic moieties (ammonium bromide salt:thymol, 1:2), facilitated the interaction with both polar and less polar compounds, resulting in an enriched cannabinoid extraction from inflorescences. However, it slightly reduced the sustainability of the process compared to the natural ESs pair. It is important to note that the US-DSLME technique was used to analyze cannabinoids in three different hemp products—aerial parts of *Cannabis sativa* L., plant inflorescences, and CBD oil—and proved to be easily adaptable to these different matrices.

Of all the methods used for extraction of bioactive compounds, UAE is the most widely employed for the extraction of cannabinoids from plant materials (Table S1 and Figure 3a). The most used solvents in US-SLE are methanol (MeOH) (entries SE27–35, SE37, SE56, SE57, SE65, SE70, SE76, SE79, SE84, and SE97), EtOH (entries SE14–20, SE37, SE52, SE76, and SE85) and acetonitrile (ACN) (entries SE36, SE37, SE58, SE59, and SE80). Interestingly, a 9:1 mixture of MeOH:chloroform (CHCl_3_) (entries SE41, SE42, SE44, SE63, SE81, SE92, and SE93) is often used, even though CHCl_3_ has been associated with CBD instability [262]. Chlorinated solvents and MeOH are not considered safe for consumer application and are also discouraged for laboratory safety reasons, as they are not classified as low-toxicity solvents and have a significant environmental impact [263]. Hence, EtOH is recommended for cannabinoid extraction because of its safety, effectiveness, and regulatory acceptance. In 2024, an extraction protocol using 96% (*v*/*v*) EtOH was included as the official cannabis flower extraction method in the European Pharmacopoeia [264].

Several parameters and factors can be critical in optimizing the efficiency and effectiveness of the extraction process, namely the amount of sample relative to solvent volume, particle size, time taken for the extraction, number of consecutive extractions, and temperature [252,255]. These experimental conditions were studied in several manuscripts gathered in Table S1 and exhibit significant variability depending on the purpose of the research and the desired selectivity during the extraction process. For example, this process can involve extraction times ranging from as little as 1 min to as much as 1 h (entries SE91 and SE80) for the extraction of, respectively, 50 mg of cannabis samples using 10 mL of an EtOH:ACN (1:1, *v*/*v*) mixture and 150 mg of leaves and vegetative shoots using 1 mL of ACN at a temperature of 55 °C [95,172]. Concerning the number of consecutive extractions or the use of extended extraction times, de Souza et al. [141] (entry SE28) found that the latter led to a decrease in THCA and Δ^9^-THC, accompanied by a corresponding increase in CBN. This suggests, as mentioned previously, that prolonged extraction times may lead to the degradation of these cannabinoids. Coherently, THCA and Δ^9^-THC recoveries were slightly lower under conditions involving two extraction steps with solvent renewal compared to a single-step extraction, supporting the hypothesis of degradation during extended extraction. These findings align with a previous study by Mudge et al. [263], which demonstrated that UAE is superior to shaking, but that extended sonication time can lead to the degradation of cannabinoids. However, de Souza et al. also noted that a complete cannabinoid recovery might not be attainable with just a single extraction step. Design of Experiments (DoE) studies considering the effect of variables such as granulometry, time, and the number of extractions on cannabinoids recovery allowed the authors to conclude that the ideal compromise among these factors involves granulometries of 180 to 250 µm of plant material and two extractions of 10 min each (entry SE28). Recently, DoE has been applied in the optimization of cannabinoids extraction protocols, constituting a valuable tool for systematically studying the relationship among multiple input factors (such as solvent type and concentration, temperature, extraction time, particle size of plant material, solid-to-liquid ratio, etc.) and the desired output, namely yield, purity, or efficiency of the process.

### 3.2. Extraction of Cannabinoids from Oils and Resins

Concerning the study of hemp or cannabis concentrates, the terms ‘resin’ or ‘oil’ are freely applied and refer often to different types of extracts, namely full-spectrum extracts, CBD isolates, distillates, crude extracts, etc. Therefore, careful attention is required when assessing the results. Ultrasound is the technique generally employed for the extraction of cannabinoids from oils and resins (Table S1, entries SE98–116 and Figure 3b). The extraction procedures are primarily defined by the type of matrices used and secondarily by the extracting solvent. For example, when extracting bioactives from oils, extraction procedures are frequently conducted in polar solvents such as MeOH (entries SE99, SE103, and SE107), EtOH (entries SE102 and SE105), isopropanol (entry 104), or by partitioning in a more complex biphasic solvent system, namely *n*-Hexane:Ethyl acetate:ACN:MeOH (8:2:8:2, *v*/*v*) (entry SE106). When considering resins as matrices, cannabinoid extraction is primarily performed using US-SLE with polar solvents such as MeOH (entry SE115) or a combination of MeOH with CHCl_3_ (entries SE112 and SE113).

### 3.3. Extraction of Cannabinoids from Food Products

The number and variety of commercially available cannabis-infused food products have increased globally. These products cover a wide range of food matrices, including juices, tea and coffee to ice cream, honey, cookies, candies, gummies, and chocolates (Table S1, entries SE117–131). Such diversity in food matrices necessarily involves adapting extraction protocols for the efficient recovery of cannabinoids. Several manuscripts focused their research on, for example, gummies matrices. In these cases, ultrasound or shaking extraction methods were employed, using water or a mixture of this solvent with MeOH, MTBE, or ACN (entries SE126–128, SE130, and SE131). Melchert et al. analyzed CBD-fortified gummy bears (entry SE126) using a US-SLE procedure with a MeOH:H_2_O (1:1, *v*/*v*) mixture [121], while Song et al. examined several hemp-infused edibles (entry SE131) using the same solvent mixture but with a higher organic solvent content (95%). In this last example, the extraction was performed by first forming a suspension of the pulverized sample in water, followed by the addition of MeOH and ultrasonication [184].

### 3.4. Extraction of Cannabinoids from Pharmaceutical and Other Formulated Products

Cannabinoids in pharmaceutical and other formulated products of medicinal interest come in diverse forms, including pills, powders, ointments, oil-in-gel emulsion, lotions and creams, bandages, vaping products, and nanoparticles (Table S1, entries SE132–157). Each type of formulation presents unique analytical challenges due to its distinct matrices, and tailored testing methods are applied to ensure accurate measurement and identification of cannabinoids within these complex matrices. Nevertheless, ultrasonication was the technique most often used in these matrices (Figure 3d). Regarding cosmetic products, the reported studies utilize ACN or MeOH in combination with either ultrasound or stirring for extraction (entries SE135–137, and SE141). Pires et al. [208] (entry SE135) studied 31 commercially available cannabis-based products in Portugal, of which nine were for cosmetic use (creams, shower gel, shampoo, and balm). The extraction procedure for cosmetic creams comprised agitating 0.5 g of the sample in ACN for 15 min, followed by centrifugation and supernatant filtration. Mouton et al. [104] also used ACN as the extraction solvent, employing ultrasonication when analyzing a total of five cosmetic products. The extraction protocol was optimized for several types of matrices, with longer times (45 min) required for the topical ones (entry SE141).

### 3.5. Extraction of Cannabinoids from Biological Materials

Cannabinoids can be extracted from various biological matrices, including urine, blood, oral fluid, plasma, hair, and different tissues (Table S1, entries SE158–162). As new cannabis-based medicinal products and technologies are developed and tested, the number of types of matrices increases due to the rise in both in vitro and in vivo studies. Analyses of Table S1 indicate that classic protein precipitation followed by LLE using immiscible solvents like hexane and ethyl acetate, along with vortexing, was applied to human and mouse plasma samples, as well as mouse lung, liver, and brain tissues (entries SE158 and SE161) [82,235]. SLE was applied using a bead-beating system at lower temperature for the extraction of cannabinoids from zebrafish larvae tissue (entry SE162) [177]. Cheng et al. [234] developed a transdermal drug-delivery system based on a CBD nanosuspension that was incorporated into dissolving microneedles (entry SE161). In this example, the authors used a rat model to test the efficacy and safety of this delivery platform, and it was necessary to quantify the amount of CBD retained in the rat skin. The processing of skin samples involved their removal, weighing, and extraction in MeOH for 24 h with continuous agitation in a shaker, followed by two periods of 30 min using ultrasound. As can be seen in Table S1, depending on the assay performed and the specific aims of the study, adequate extraction protocols need to be designed.

In summary, regarding the manuscripts collected in Table S1, advanced methods such as SFE, UAE, and the use of DESs to extract cannabinoids were the most commonly applied extraction methods. It is important to note that while cannabinoids can be extracted from different matrices using a diverse array of techniques, each method has unique limitations. SFE offers clean, solvent-free extracts with high selectivity and efficiency; however, its accessibility is limited by high equipment costs and complexity. Similarly, techniques like UAE, though effective and rapid, can lead to the thermal degradation of heat-sensitive compounds if not carefully optimized, and may have poor scalability. UAE is clearly the most widely applied technique across all matrices as can be seen in Figure 3. EtOH extraction assisted by ultrasound is the most used technique for plant material matrices, as it is efficient at extracting a wide range of cannabinoids. Additionally, maceration is also often used, although it is a slower and less efficient process. The use of ESs in the extraction of cannabinoids is at an early stage of research. However, they are gaining popularity for the extraction of natural products due to their compatibility with most extraction techniques, lower toxicity, enhanced biodegradability compared to conventional solvents, and the fact that they are often derived from natural and less expensive materials. Regarding the extraction of cannabinoids from complex matrices, namely biological samples such as plasma and tissues, the traditional LLE method using immiscible solvents combined with vortexing is commonly employed. This technique is preceded, in the studies reviewed, by protein precipitation, which is used as a pre-treatment step before the extraction process. Furthermore, adding steps such as solid-phase extraction (SPE), can be beneficial, if applicable, resulting in cleaner samples, albeit at a higher cost. For example, Mastellone et al. [93] used SPE to remove chlorophylls from the plant extracts (entry SE94).

## 4. HPLC-UV Analysis

Among the several separation techniques available, LC is generally the preferred method for determining the cannabinoid profile of the cannabis plant. Its advantage, for instance, over GC lies in its ability to directly analyze all cannabinoids, including acidic precursors, without the need for derivatization. Derivatization is essential in GC because the high temperatures used cause acidic cannabinoids (AC) to decarboxylate into their neutral forms. Moreover, thermal degradation (e.g., oxidation, isomerization) of cannabinoids can potentially occur in the GC injector port and column, leading to inaccurate results [10,265].

For the analysis of cannabinoid content, Ultraviolet (UV) and Mass Spectrometry (MS) are widely used [24]. MS enhances the sensitivity and specificity of analyses, which is crucial for quantifying minor cannabinoids, especially in biological matrices [9]. However, HPLC-UV remains the favored method for analyzing cannabis plant samples due to its cost-effectiveness and operational benefits [17,21], making it more accessible for cannabis growers and commercial suppliers [184]. In 2024, with the addition of a cannabis monograph to the European Pharmacopoeia [264], an official HPLC-UV method was established for the quantification of CBD, CBDA, CBN, Δ^9^-THC, and THCA.

This section describes what has been done with LC coupled with UV detection for the identification and quantification of phytocannabinoids from 2022 to 2024 (Table 1 (entries 1–65), Table 2 (entries 66–104), Table S2 (entries SH01–12), and Table S3 (entries SH13–34).

### 4.1. Stationary Phases

Almost all studies used reversed-phase (RP) octadecyl (C18) packed columns, with five studies employing C18-Pentafluorophenyl (PFP) columns (entries 15, 101, and SH18), and one a column with mixed aromatic functionality (C18-AR) (entry SH28). In addition, two studies used octyl (C8) columns (entries 68 and 73). The most frequently used columns, each featured in at least five studies, were the following:

- (i) particle size > 2 μm: Ascentis Express C18 (150 × 3.0 mm, 2.7 μm), Waters Cortecs Shield RP18 (150 × 4.6 mm, 2.7 μm), InfinityLab Poroshell 120 EC-C18 (150 × 3 mm, 2.7 μm), NexLeaf CBX for Potency C18 (150 × 4.6 mm, 2.7 μm), Phenomenex Kinetex C18 (150 × 4.6 mm, 2.6 μm), and Phenomenex Luna C18(2) (150 × 4.6 mm, 5.0 μm), all with a length of 150 mm.
- (ii) particle size < 2 μm: Waters Cortecs UPLC C18 (100 × 2.1 mm, 1.6 μm) and Phenomenex Kinetex C18 (150 × 2.1 mm, 1.7 μm).

While using a guard column with the same stationary phase is recommended, especially when analyzing complex matrices [266], only about a quarter of the studies reported its use.

Since its introduction in 2004, the Ultra High-Performance Liquid Chromatography (UHPLC) system has gained significant popularity, offering numerous advantages over HPLC, including higher sensitivity, enhanced resolution and efficiency, reduced analysis time, and lower solvent consumption [9,267]. These benefits are particularly crucial when analyzing complex matrices, such as those found in cannabis analysis. Among the previously cited columns, UHPLC systems can operate with columns that have smaller particle sizes (1.7 µm) and smaller inner diameters (2.1 mm). Another notable advancement in column technology is the use of superficially porous particles, often referred to as core-shell or fused-core particles, which enhance performance [267,268]. This column chemistry merges the advantages of both fully porous and nonporous particles and currently represents the majority of columns utilized for cannabinoid separation. Furthermore, although most of the columns discussed are C18 types, there are subtle differences in their stationary phases: Poroshell 120 EC-C18, Kinetex C18, and Ascentis Express C18 contain dimethyl-octadecyl stationary phases, while Raptor ARC-18 features a diisobutyl-octadecyl phase, and Cortecs Shield RP-18 and Ascentis Express RP-Amide incorporate embedded polar functional groups. These minor variations affect column polarity, which, in turn, influences cannabinoid separation and may be beneficial depending on the analysis objectives. A recent study by Song et al. [269] evaluated four of these column types (Poroshell 120 EC-C18, Raptor ARC-18, Cortecs Shield RP-18, and Ascentis Express RP-Amide), all sharing the same dimensions (150 mm × 2.1 mm) and core-shell particle size (2.7 μm) for the separation of 18 cannabinoids. Their results indicated that when using HPLC-DAD for cannabinoid quantification, the two conventional RP stationary phases (Poroshell 120 EC-C18 and Raptor ARC-18) provided the best results, offering shorter run times under similar conditions. Szijj et al. [243] (entry SH28) used a C18-AR column. The choice of a column with mixed aromatic functionality was based on a prior study conducted by Ciolino et al. [262], which investigated four columns of the same dimensions (250 × 4.6 mm, 5 µm) with different derivatized silica stationary phases: Luna C18(2), phenylhexyl (Luna), and two columns featuring mixed C18/aromatic functionality (ACE 5 C18-AR and Siliachrom C18/phenyl). They reported that the ACE 5 C18 column was the only one capable of resolving all nine tested cannabinoids under the chromatographic conditions examined.

As an alternative to reversed-phase liquid chromatography, De Luca et al. [270] investigated several polar-bonded stationary phases (Si, CN, Diol, and NH_2_ absorbents) and their impact on cannabinoid separation. A promising condition was achieved when normal phase with NH_2_ adsorbent was employed for the separation of five neutral cannabinoids (CBC, CBD, CBG, CBN, and Δ^9^-THC) using as mobile phase a mixture of heptane/isopropanol 95:5% (*v*/*v*). The same group also investigated different polysaccharide-based (cellulose and amylose) chiral stationary phases. They achieved the best results using CHIRALPAK IC and IF columns for the separation of CBD, CBDA, THCA, (-)-Δ^9^-THC, and both CBC enantiomers, with ACN and phosphoric acid mixtures as the eluent [271]. In addition, Russo et al. [272] were able to achieve enantioseparation of *trans*-CBD, *trans*-CBDA, *trans*-Δ^9^-THC, and *trans*-Δ^9^-THCA. The best separation was obtained using a CHIRALPAK AD-RH column with different percentages of organic modifiers in the mobile phase. Other CHIRALPAK columns (IA, ID, IE, and IG) were also shown to deliver good separations of CBD enantiomers [273]. Huang et al. [216] conducted a comparative study using two HPLC-DAD-MS methods for the separation of CBD, Δ^(4)8^-iso-THC, Δ^8^-iso-THC, Δ^8^-THC, and Δ^9^-THC. One method employed a reversed-phase column (entry 83), but only the method using a normal-phase silica-Ag(I) column (entry 104) effectively separated these five isomers.

### 4.2. Mobile Phases and Type of Elution

Separating the main cannabinoids via LC can be challenging; therefore, several studies favor gradient elution (Table 1 and Table S2) over isocratic elution (Table 2 and Table S3). Generally, gradient methods allow for shorter run times, higher flow rates, and better peak resolution [14]. The most common solvent system was ACN and water acidified with 0.1% formic acid.

Regarding the choice of organic solvent (B), ACN was, as expected, the preferred option by far (entries 1–47, 66–99, SH01–10, and SH13–29) over MeOH (entries 48–60, 100–102, SH11, SH12, and SH30–33). ACN offers a few advantages in comparison to alcohols: (a) its lower viscosity results in less system backpressure, consequently enabling higher flows; and (b) its lower UV absorbance at wavelengths most frequently used for cannabinoid analysis can lead to increased method sensitivity and baseline uniformity [274]. Nevertheless, each organic modifier offers distinct characteristics that may be valuable to explore; for instance, MeOH has some acidic properties, while ACN exhibits dipolar properties [275]. As an example, Franklin et al. [276] were able to obtain a better separation of Δ^9^-THC, Δ^8^-THC, and CBL when a mixture of 50:50 ACN and MeOH was used. Six studies reported the use of both ACN and MeOH (entries 61–65, and 103).

The most used aqueous phase (A) was water acidified with 0.1% formic acid (entries 7–26, 48–55, 62, 63, 71–82, SH03–06, and SH20–22). Adding pH-modifying agents to the mobile phase can influence the selectivity factor by increasing ionic strength and altering the pH [117]. AC will be more affected, showing greater retention at lower pH values due to the prevalence of their non-ionized form, whereas NC are much less impacted by mobile phase pH changes [141]. Moreover, acid additives play an important role in achieving optimal peak shape with minimum tailing, especially for AC [276,277]. The choice of acid modifier can also affect baseline uniformity in the resulting chromatogram. Ryu et al. [278] tested 0.10% formic acid, 0.05% trifluoroacetic acid, and 0.10% phosphoric acid under gradient conditions and reported that formic acid produced a more pronounced raised baseline than trifluoroacetic acid, while phosphoric acid had minimal impact. In addition to formic acid, other researchers have employed different agents to adjust the pH of the aqueous phase, including trifluoroacetic acid (entries 60, 96–99, 102, SH10, and SH24), ammonium formate (entries 28–36, 56, 65, 84–94, SH11, and SH23), phosphoric acid (entries 37–45, 57, 64), acetic acid (entries 46, 47, 103, SH08, SH09, SH25–28), and ammonium acetate (entries 58 and 59). Only a few studies did not incorporate a pH modifier (entries 1–3, 66–69, SH01, SH13–19, SH30–32, and SH34).

Furthermore, the mobile phase is also optimized to ensure the best separation of the target cannabinoids without interference from other matrix components. It is important to note that in some of these investigations, the goal was to characterize the matrix composition and not only to assess cannabinoids, leading to long runs covering a wide range of solvent gradient (e.g., entries 9, and 10).

### 4.3. UV Detection

Detection depends on the UV absorption capability of the analyte. In cannabinoids, the primary chromophore is a substituted phenolic ring (Figure 1). Further contributions to the UV spectrum occur when cyclization of the non-phenolic part is present, leading to a conjugated double bond (CBC and CBCA) or the formation of a second phenyl ring (CBN and CBNA) [17,265]. Consequently, many cannabinoids exhibit similar UV spectra, which can result in low specificity. To address this limitation, diode array detection (DAD) can be valuable for peak characterization, as the absorption spectra differ, at least between neutral and acidic cannabinoids. Additionally, exploring these UV spectral differences can also enhance sensitivity by enabling the measurement of various cannabinoids at distinct wavelengths. For example, CBD, CBG, Δ^9^-THC, and NC in general, exhibit a single absorbance peak around 210 nm, while their corresponding acids show a primary peak near 220 nm, along with two smaller peaks around 260–270 nm and 300 nm [157,158,194,279,280]. These absorption profile differences have been applied to enhance method sensitivity for NC and AC, as demonstrated by the following examples: NC-210 nm, AC-220 nm (entries 7, 14, and 28), NC-210 nm, AC-221 nm (entry 17), NC-210 nm, AC-225 nm (entry 15), NC-210 nm, AC-228 nm (entry 88), NC-210 nm, AC-284 nm (entry 73), NC-225 nm, AC-306 nm (entry 43), and NC-228 nm, AC-306 nm (entry 45). For the main NC, exceptions to this absorbance spectrum trend are seen with CBN and CBC. They display two absorbance peaks, one around 220–230 and a second around 280–285 nm [129,180,194], enabling them to be also analyzed at the same wavelengths as AC (entries 7, 15, and 73). Their corresponding acid (CBCA and CBNA) also exhibit a different absorbance profile, with only one major peak around 254–262 [130,180,194]. Song et al. [157,182,184] used 262 nm for quantification of CBCA and CBNA (entry 61) or, individually, at 251 nm and 261 nm, respectively (entries 84 and 86), and 284 or 285 nm for CBC and CBN. These two NC were also detected at 283 nm by Carona et al. [82] (entry 3).

When a single wavelength was used, it was typically set between 210 and 240 nm with 220 nm and 228 nm being the most frequently used. A few exceptions were reported: 269 nm (entry 75), 270 nm (entries 36, SH04, and SH05), 272 nm (entry 68), 275 nm (entry 94), and 280 nm (entries 71 and 77). Barhdadi et al. [173] stated that by using 269 nm they were able to decrease the UV-absorbance of selected interfering terpenes; Križman [281] reported improved sensitivity (tenfold reduction in signal noise) at 275 nm compared to 228 nm, and Benkirane et al. [90] selected 280 nm since it is a typical maximum absorption wavelength for phenolic compounds. In addition, Song et al. [182] quantified cannabinoids using two wavelengths to increase method specificity (entry 84).

To validate and/or improve specificity, some authors used MS for cannabinoid assignment and qualitative evaluation, and UV for quantification (e.g., entries 14, 25, 29, 70, 75, 88, 98, 100, and 102). MS was also used to quantify cannabinoids below the limit of quantification (LOQ) obtained by UV (e.g., entry 88). In recent years, hyphenated dual techniques such as LC-UV-MS have become increasingly popular due to their enhanced sensitivity and specificity [24].

### 4.4. Retention Time and Difficult Resolutions

Retention times will differ depending on the solvent system and gradient employed. However, since almost all publications included in this review used reversed-phase columns, general considerations can be made based mainly on lipophilicity [270,282,283]:

- CBDV(A) and THCV(A) have shorter retention times than their homologous CBD(A) and Δ^9^-THC(A) due to their shorter C3-side chains (n = 3 vs. 5, respectively), which leads to decreased lipophilicity.
- Cannabinoids with two hydroxyl groups are more polar than their one-hydroxyl chromane or chromene counterpart, leading to shorter retention times (e.g., CBD, CBDV, and CBG vs. Δ^9^-THC, THCV, and CBC, respectively).
- Δ^9^-THC tends to elute slower than its oxidative product, CBN, due to a decrease in lipophilicity caused by the two extra double bonds formed during ring aromatization.
- CBC(A), which has two aliphatic side chains, is slightly less polar than Δ^9^-THC(A), resulting in longer retention times.

The retention times of NC compared to their acidic counterparts vary depending on several factors, such as pH, eluent system, and gradient. For instance, Büttenbender et al. [154] (entry 58) used a gradient of 10 mM ammonium acetate (pH 5.2) and MeOH, resulting in all AC eluting before any NC. Song et al. [213] (entry 102) noted that an elution system of water and MeOH exhibited a stronger retention for AC, leading to a significantly altered elution order of cannabinoids compared to the water and ACN elution system they tested.

When optimizing chromatographic conditions, among the most frequently detected cannabinoids, particular attention should be given to the CBD/CBG and Δ^8^-THC/Δ^9^-THC pairs. The CBD/CBG pair differs by a closed versus open ring side chain, while Δ^8^-THC and Δ^9^-THC vary by the position of a single double bond, resulting in similar polarity in both cases. These structural similarities can lead to co-elution, and their comparable UV spectra make accurate identification and quantification more challenging. Furthermore, as previously noted, these NC are less responsive to changes in the mobile phase pH. De Souza et al. [141] (entry 46), using DoE-assisted development, found that, within their design parameters, the CBD/CBG pair achieved better resolution at lower temperatures and with a flow rate close to 0.8 mL/min, while the Δ^8^-THC/Δ^9^-THC pair benefited from higher temperatures and flow rates. In both cases, extending the initial gradient (without altering the starting ACN percentage) improved resolution. Büttenbender et al. [154] (entry 58) also reported improved resolution between CBD and CBG when the temperature was decreased. Roussel et al. [105] (entry 18), applying prediction intervals for robustness, observed increased CBD/CBG resolution with higher temperatures and lower flow rates for their solvent system. Song et al. [182] (entry 84), in preliminary studies, reported improving the resolution of both pairs to the required minimum acceptable value of 1.5 by increasing the stationary phase length. In another work, Song et al. [213] (entry 102) found that the resolution for Δ^9^-THC/Δ^8^-THC was improved in a water/MeOH mobile phase, while the resolution for CBD/CBG was inferior compared to a water/ACN eluent. Focusing on the Δ^9^-THC/Δ^8^-THC pair, Pittiglio et al. [134] (entry 41) developed a method that allows baseline resolution at 500 ppm.

Additional cases of co-elution can also pose challenges, depending on the specific analytical method developed. For instance, Song et al. [168] (entry 70) were not able to separate THCVA and CBG under the primary conditions stablished. However, a second run of the sample with a different column temperature (45 vs. 30 °C) and detection at 269 nm instead of 230 nm, was sufficient to obtain good THCVA/CBG resolution.

Al-Maraseemi et al. [236] (entry SH19) evaluated retention time, peak areas, and tailing factors of CBD using Box–Behnken design experiments. They identified ACN composition as the most critical parameter affecting retention time, while the flow rate and oven temperature were the most significant factors influencing the tailing factor and peak area.

## 5. Analytical Method Validation

The validation of an analytical method is the process of proving that a specific method is suitable for its intended purpose by evaluating its performance characteristics under a variety of conditions [284]. Defining its intended use will have a major impact on the validation protocol and the established parameter limits. For instance, surveillance methods will prioritize speed and may tolerate lower precision and a higher rate of false positives, while dispute resolution methods will demand higher accuracy, precision, and reproducibility, making assay time less critical [285]. Nevertheless, method validation, which ensures the reliability, accuracy, and reproducibility of data, is crucial in regulated industries where product safety and quality are the highest priority [286].

### 5.1. Validation Guidelines

These guidelines provide a framework for analyzing key analytical performance characteristics for method validation such as specificity, linearity, reportable range, precision, accuracy, lower range limits, robustness, and system suitability. Several guidelines and compendiums were mentioned in the selected papers for validation analysis (Table S4, entries SV01-50), such as the following:

- International Council for Harmonisation of Technical Requirements for Pharmaceuticals for Human Use—ICH guidelines: Validation of analytical procedures Q2 (R1) [287] and Q2 (R2) [288]; M10 on bioanalytical method validation and study sample analysis [289]—22 papers.
- Association of Official Analytical Collaboration (AOAC) International guidelines: Appendix F: Guidelines for Standard Method Performance Requirements [285]; Appendix K: Guidelines for Dietary Supplements and Botanicals [290]; Standard Method Performance Requirements (SMPRs^®^) for Quantitation of Cannabinoids in Plant Materials of Hemp (Low THC Varieties Cannabis sp.) [291]—10 papers.
- International Organization for Standardization (ISO) 17025: General requirements for the competence of testing and calibration laboratories [292]—9 papers.
- United States Food and Drug Administration (FDA): Analytical Procedures and Methods Validation for Drugs and Biologics—Guidance for Industry [293]; ORA Lab Manual Vol. II—Methods, Method Verification and Validation (ORALAB.5.4.5) [294]—6 papers.
- Agência Nacional de Vigilância Sanitária (ANVISA) RDC Nº 166/2017 [295]—3 papers.
- Eurachem group—The Fitness for Purpose of Analytical Methods [296]—3 papers.
- United States Pharmacopeia (USP) (e.g., [297])—3 papers.
- American Academy of Forensic Science: Standard Practices for Method Validation in Forensic Toxicology (ANSI/ASB Standard 036) [298]—2 papers.
- SANTE 11312/2021—Analytical Quality Control and Method Validation Procedures for Pesticide Residues Analysis in Food and Feed [299]—2 papers.
- United Nations Office on Drugs and Crime (UNODC): Guidance For the Implementation of a Quality Management System in Drug Testing Laboratories [300]—1 paper.
- Scientific Working Group for Forensic Toxicology (SWGTOX): standard practices for method validation in forensic toxicology [301]—1 paper.

Notably, several papers combined multiple guidelines, with ICH being the most frequently included. Although these guidelines share common principles, they vary in their scope, focus, and application, reflecting the diverse needs of different sectors. In this section special attention will be given to three validation parameters—accuracy, precision, and lower limits—and to the most cited guidelines: ICH and AOAC.

### 5.2. Precision

The precision of an analytical procedure reflects the closeness of agreement, or degree of scatter, between a series of measurements obtained from multiple samplings of the same homogeneous sample under the specified conditions [299]. Precision can be assessed in terms of repeatability, intermediate precision, and reproducibility.

Repeatability, also referred to as intra-assay precision, refers to the precision of measurements obtained under identical operating conditions within a short timeframe. Several authors also report repeatability as intra-day precision. ICH Q2 recommends at least nine determinations covering the established linear range or a minimum of six determinations when analyzing the target concentration at 100%. Most frequently, the reviewed papers used three quality control (QC) concentration levels of the selected calibration curve: low, medium, and high, analyzed in triplicate. ICH M10 recommends a fourth QC corresponding to LOQ to evaluate precision and accuracy. However, overall, the investigated papers used between one to six concentrations, and between three to twelve injections per concentration to investigate repeatability.

Intermediate precision reflects the variability observed within a laboratory. Common variations that can be evaluated include different days (inter-day), analysts, equipment, and environmental conditions. In most studies, inter-day precision was assessed over a period of three days with a few papers using two days (entries SV01, SV13, SV31, and SV42); five days (entry SV38); six days (entries SV14 and SV37); and ten days (entry SV41). Less frequently, intermediate precision was also evaluated using different analysts (entry SV01 and SV02). Lastly, reproducibility involves evaluating inter-laboratory precision using the same method.

In most works, the precision of the method was expressed as relative standard deviation (RSD%) or coefficient of variation (CV%) and calculated according to Equation (1), where “SD” represents the standard deviation and “μ” the average of the data [298].

Depending on the scope of the papers, the procedure to assess precision ranged from merely evaluating the instrument precision using standard solutions of the analyte to incorporating complete sample preparation with different analysts, operating conditions, and equipment. Furthermore, the scope of the analysis determines the variability expected and accepted; precision typically decreases as concentration diminishes and as matrix complexity increases, especially in biological samples [284]. For that reason, the ICH M10 guideline established that precision should be within ±15% at each level of the linear range, but allowing ±20% at LOQ. The ANSI/ASB Standard 036 guideline establishes a maximum RSD of 20%, but notes that certain methods require RSD limits of 10% or lower. The AOAC SMPR^®^ 2019.003 guideline, which is specific for cannabinoids in plant materials, recommends different limits depending on the concentration of the analyte (% on dry weight basis): 0.05–0.5%: RSD ≤ 5% and ≤10%; >0.5–5%: RSD ≤ 3% and ≤8%; 5–35% (just for CBD and CBDA): RSD ≤ 2% and ≤6% for repeatability and reproducibility, respectively. Conversely, for drug products and drug active pharmaceutical ingredients, the FDA establishes a repeatability RSD of less than or equal to 3.0% and 2.0%, respectively [294]. In the analyzed papers, some authors also defined distinct limits for different precisions: RSD not exceeding 5% and 10% (entries SV30 and SV31) or 15% and 30% (entry SV36) for repeatability and intermediate precision, respectively.

For assay method validation, some authors include, as system suitability tests, other precision parameters such as the precision for retention time, peak area, peak height, or tailing factor (e.g., entries SV01, SV02, SV04, and SV05):

RSD% = SD/μ × 100(1)

### 5.3. Accuracy

As described by ICH Q2, accuracy, sometimes referred to as trueness, in an analytical procedure refers to how closely the results obtained agree with the true value or an accepted reference value [287,288]. Related to accuracy, the AOAC guideline uses the term “bias” to describe the difference between the expected test result and an accepted reference value [285]. Accuracy should be determined across the analytical procedure’s entire reportable range and demonstrated under standard analytical procedure conditions, including, when possible, sample matrix effects and the sample preparation protocol. As an example, ICH Q2 suggests testing accuracy using three concentration levels in triplicate across the established range. There are a few ways that accuracy can be determined:

- Comparison to a reference standard or to an already validated procedure.

Accuracy can be assessed by applying the analytical method to a known-purity analyte, such as a reference standard. Ideally, to prevent biased results unrelated to the method’s performance, calibration standards and QC samples should be prepared from different stock solutions [302].

Some studies used QCs prepared solely in solvent to assess validation parameters. However, for analytes in products, recovery experiments from matrices before and/or after extraction should also be performed to investigate potential matrix effects. For example, Song et al. [157,168,182,184,213] (entries SV19-23) used QCs prepared in MeOH containing an internal standard to assess accuracy and precision. However, all their analyzed hemp-infused samples were spiked with the non-naturally occurring cannabinoid ACBD to track recovery for every sample preparation. Likewise, Correia et al. [172] (entry SV34) verified the complete cannabinoid extraction recovery from a surrogate matrix to ensure that validation parameters obtained from neat standard solutions were valid.

- Recovery of the analyte spiked into blank matrix or surrogate matrix.

The procedure involves spiking a formulation blank (containing all non-analytes) with a precisely measured quantity of the pure analyte. Ideally, the spiked sample is then subjected to both sample preparation and HPLC analysis, and the obtained results are compared with the predicted values [284]. However, the presence of cannabis extracts in formulations complicates recovery experiments due to the challenge of obtaining a suitable cannabinoid-free cannabis blank matrix, leading several authors to resort to surrogate matrices. In the reviewed papers, for the cannabis inflorescence matrix, Hall et al. [162] (entry SV02) used chamomile as a surrogate matrix, reporting it as an inexpensive floral material without cannabinoids that shares similar phytochemicals (terpenes and flavonoids) with cannabis. Correia et al. [172] (entry SV34) used hops from *Humulus lupulus*, a member of the Cannabaceae family like the cannabis plant, and Pires et al. [208] (entry SV38) used flowers from *Urtica dioica* to mimic cannabis flowers and herbal tea samples to perform spiking experiments before and after extraction.

A few studies employed matrices with residual levels of cannabinoids as blank matrices. Leersnijder et al. [174,175] (entry SV17) used fiber-type herbal cannabis, with negligible cannabinoids, as a blank matrix for recovery testing. Chan [164] (entry S41) used a sample matrix obtained by extracting only a small amount of cannabis samples, with subsequent dilutions before the spiking experiments if the analyte concentration was still elevated. Other authors used cannabis plant waste material obtained after extraction procedures as the blank matrix for validation studies [151,169,180] (entries SV03, SV32, and SV37). In these cases, during spike recovery experiments, if necessary, residual background cannabinoids were subtracted from the results obtained, as in the standard addition method.

Several oils were employed as surrogates for cannabis oil products: coconut oil [218] (entry SV26), MCT oil [243] (entry SV08), olive oil [162,181,244] (entries SV02, SV11, and SV13), sunflower oil [181] (entry SV13), and sunflower and coconut oil (60:40) [237] (entry SV28). For formulated products with cannabis extracts/cannabinoids, the spiking experiments were performed in cannabis-free formulations comparable to those examined [186,216,246] (entries SV25, SV29, and SV50). In addition, Melchert et al. [121] (entry SV45) used honey (rapeseed flower) as a surrogate matrix for formulated products, and Carona et al. [82] (entry SV09) used human plasma and mouse matrices without the investigated analytes as blank matrices for validation studies.

- Standard addition method.

Given the inherent presence of cannabinoids within the plant matrix, a spiking methodology using standard addition was commonly applied. In these cases, spiked and unspiked samples are analyzed, and the cannabinoid recovery corresponds to the sum of the added and naturally occurring amounts (entries SV05-07, SV14, SV16, SV27, SV30, SV31, SV35, SV36, and SV40). Chaiwangrach et al. [192] (entry SV16) used enriched cannabinoid mixtures prepared from cannabis samples for accuracy studies instead of pure standards.

When reported, accuracy was calculated using Equation (2), where “CD” is the concentration determined, and “CA” is the known concentration. For standard addition methodology, recovery was determined using Equation (3) where “CS” is the concentration determined for the standard added plus the endogenous amount of the cannabinoid (spiked sample), “CU” is the determined endogenous amount in the sample (unspiked sample), and “CA” is the known concentration of the standard solution added [99,103,111,133].

A smaller subset of studies reported accuracy as bias % (entries SV08, SV09, SV25, SV34, SV37, and SV50), calculated using the Equation (4) [169,243] or as mean relative error % (entry SV38). Furthermore, some studies evaluated accuracy/trueness using a total error approach, with relative bias, accuracy profiles, and β-expectation tolerance intervals (entries SV17 and SV18) [173,174,175]:

Accuracy% = C_D_/C_A_ ∗ 100 (2)

Accuracy% = (C_S_ − C_U_)/C_A_ ∗ 100 (3)

Bias% = (C_D_ − C_A_)/C_A_ ∗ 100 (4)

As observed with precision, the AOAC SMPR^®^ 2019.003 guideline recommends limits as a function of cannabinoid concentration (% on dry weight basis): 0.05–0.5%: 85–118%; >0.5–5%: 90–111%; 5–35% (just for CBD and CBDA): 95–105% for recovery experiments using the whole procedure (limits used by entry SV30). Some papers used AOAC appendix F limits for recovery: 0.1%: 90–107% (entry SV29) and 0.01%: 80–110% (entry SV28). ICH M10 establishes that accuracy should be within ±15% of the nominal concentration at any level of the linear range, except at the LOQ, which allows variation within ±20% (limits used by entries SV09 and SV38). Some authors established limits of 80–120% (entries SV07, SV12, SV37, and SV47), 90–110% (entry SV10), 80–125% (entry SV13) for recovery and ≤20% (entry SV25) or ≤15% (entry SV08, and SV34) for % bias or error. Song et al. [184] (entry 21) used 80–120% for quality control at low concentration and 85–115% for medium and high concentration controls. The widest interval deemed acceptable by the authors was 70–120% (entry SV26), as described in SANTE guideline as acceptable during initial validation stages.

### 5.4. Lower Range Limits

When the analysis requires the analytical procedure to operate close to its lower range, establishing a limit of detection (LOD) or detection limit (DL), and especially a limit of quantification (LOQ) or quantitation limit (QL), is paramount. LOD represents the lowest concentration of an analyte that can be reliably detected, though it remains too small to be measured with certainty under the specified experimental conditions. Conversely, LOQ is the lowest concentration of an analyte that can be quantitatively measured with a previously defined level of acceptable precision and accuracy [284]. They can be estimated using different methods:

- Based on signal-to-noise ratio (S/N).

The S/N is determined by comparing signals from samples containing low concentrations of the analyte to signals from blank samples. Alternatively, the peak-to-peak noise amplitude can be measured around the analyte retention time and compared with the analyte signal height. A S/N of at least three and ten is considered suitable for estimating LOD and LOQ, respectively [218,288]. Twenty-four papers reported these S/N ratios to assess LOD and LOQ. In one case, LOD was also established as S/N ≥ 2 [218] (entry SV26).

- Based on visual evaluation/Based on accuracy and precision at lower range limits.

The limits are found through analysis of known low concentration samples, identifying the lowest level at which the analyte can be reliably detected or quantitated. In these cases, LOQ can be directly validated by accuracy and precision measurements [164,172,208,244] (entries SV11, SV34, SV38, and SV41). In fact, as mentioned before, the ICH M10 guideline recommends that one of the concentrations used during precision and accuracy validation should be the LOQ [289]. Carvalho et al. [151] (entry SV32) established the LOD as the lowest concentration that yielded a signal greater than noise with RSD ≤ 10%, while adopting LOQ as the lowest concentration of the calibration curve. Likewise, Song et al. [168,182] (entries SV19 and SV23) also established LOQ as the lowest concentration of the linear range. Meanwhile, Chan [164] (entry SV41) reported LOD as the lowest concentration tested even though lower concentrations were likely detected, and Barhdadi et al. [173] (entry SV18) simply established LOQ as the lowest validated concentration level. In addition, entries SV34 and SV41 also evaluated the S/N of the signals obtained in these experiments. Although some papers did not directly specify the method used to determine LOQ, they used this concentration for precision and accuracy experiments (e.g., [82] entry SV09).

- Based on standard deviation of a linear response and a slope.

The lower limits can also be estimated by applying Equations (5) and (6), where “σ” represents the standard deviation of the response and “S” the slope of the calibration curve. In the analyzed papers, when specified, “σ” was calculated as follows: (i) standard deviation of the blank (entries SV05 and SV50); (ii) standard deviation of the lowest linear concentration (entry SV02); standard deviation of the lowest response compound that yielded the most conservative value (for LOD) (entry SV25); (iii) standard deviation of the slope (entry SV12); (iv) standard deviation of y-intercepts (entry SV28); and (v) residual standard deviation of the regression line (entries SV06, SV07, and SV36). Furthermore, in other works, the type of standard deviation “σ” was not specified (entries SV10, SV14, SV29, SV31, SV33, SV40, and SV44). ICH Q2 describes “σ” as the standard deviation of (i), (iv), and (v) and recommends that when the estimation is based on a calibration curve, it should be constructed with samples with analyte concentrations that fall within the lower limits range. When LOD and LOQ are obtained by estimation from analytical curves, ICH also recommends further validation by analyzing an appropriate number of samples that are known to be at or near the LOD and LOQ [288]. In some studies, experiments to evaluate the S/N ratio (entries SV10, SV12, SV25, and SV31) or to evaluate accuracy and precision (entries SV28, SV33, and SV50) were also performed to verify the estimated values:

LOD = 3.3*σ*/*S* (5)

LOQ = 10*σ*/*S* (6)

### 5.5. General Matrix Interference Considerations

When working with a new matrix, two questions must be addressed: (i) How effectively can the analytes be extracted from the sample matrix? (ii) Do the co-extracted components of the matrix interfere with the detection and quantification of the analytes?

As discussed in Section 4.1, careful selection of HPLC conditions is essential to ensure method specificity and achieve good resolution between the cannabinoids of interest, as well as between them and other matrix interferences. Additionally, general strategies to mitigate matrix effects during analysis can be employed, such as adjusting the retention time of the analytes to avoid the solvent front, where polar and non-retained compounds elute, and the end of the chromatographic gradient, where a higher concentration of organic solvent leads to the elution of the strongly retained nonpolar compounds [303,304].

Furthermore, understanding the impact of the sample matrix on analyte recovery is crucial for developing extraction methodologies. This knowledge will help elucidate how incorporating clean-up steps—if needed to mitigate matrix effects during analysis—will influence overall efficiency. These studies are particularly important when multiple analyte quantifications are targeted, as extraction may impact each analyte differently. For example, Dawson et al. [305] studied the impact of changes in chocolate matrices on cannabinoid extraction efficiency and, consequently, quantification using the same extraction protocol. They reported that the extent of interference from the chocolate matrix on cannabinoid analysis is influenced by both the quantity of chocolate present and the chemical structure of the cannabinoid analyte. Increased amounts of chocolate in the sample correlate with decreased percent recovery of the analyte, and this effect is much more pronounced as cannabinoid polarity decreases.

When analyzing various oil samples, it is essential to consider the distinct properties of different carrier oils, especially if they are also used as solvents during cannabinoid extraction from the cannabis plant, as these factors can influence extraction efficiency and matrix complexity [256,306]. Given that several papers reviewed employed surrogate oils for the validation of these preparations, it is crucial to conduct proper matrix validation studies to ensure that the selected surrogate matrix accurately represents all the oil preparations analyzed. These studies become increasingly important as the matrix complexity increases, such as in cannabis edibles.

Due to the potential interference of the matrix, certain performance parameters should be determined when validating a new analytical procedure for analyte quantification: the matrix effect (ME), the recovery (RE) of the extraction procedure, and the overall process efficiency (PE). These can be calculated using Equations (7)–(9), where A represents the peak response of the analyte in the solvent standard, B represents the peak response of the analyte in the matrix spiked after extraction, and C represents the peak response of the analyte in the matrix spiked pre-extraction [307,308]. The ME calculated in this manner is known as absolute ME. To evaluate MEs across various matrices, it is important to assess relative ME by measuring the peak response of the analyte in different matrix lots that have been spiked after extraction, with the results expressed as RSD (%). Additionally, assessing relative MEs is especially important when employing matrix-matched calibration or background subtraction calibration techniques to mitigate matrix interference [309,310]:

ME (%) = B/A ∗ 100(7)

RE (%) = C/B ∗ 100(8)

PE (%) = C/A ∗ 100 (9)

## 6. Green Analytical Chemistry Considerations

The 12 principles of green analytical chemistry proposed by Gałuszka et al. [311] emphasize the importance of minimizing environmental impact and enhancing analytical efficiency in scientific practices while guaranteeing operator safety. Key practices should include minimizing sample treatment and sample sizes. Researchers should adopt HPLC techniques that promote faster and more efficient cannabinoid separation while minimizing waste production. For example, regarding column optimization, researchers should consider reducing the size (diameter and length) of the columns, using core-shell particles, employing columns with smaller particle sizes, and, if possible, adopting more polar reversed-phase columns instead of C18 [312,313].

In the discussed reversed-phase HPLC analytical protocols, ACN and MeOH were used as the preferred organic modifiers in all referenced papers. However, their toxic and flammable nature presents significant hazards. ACN can be metabolized in the liver into toxic compounds such as hydrogen cyanide and formaldehyde, and chronic exposure to it can lead to adverse health effects. In addition, even low-level exposure to MeOH, whether through inhalation or skin contact, can harm the nervous system, liver, and kidneys [313]. Therefore, it is of primary importance to seek alternative organic solvents for mobile phase to promote a greener LC separation of cannabinoids.

Regarding the extraction of cannabinoids from various matrices, the most commonly used technique in the reviewed papers is UAE. This method is considered a green extraction technique due to its enhanced efficiency and reduced solvent consumption. Additionally, UAE shortens extraction time, lowers analysis costs, and minimizes the potential degradation of cannabinoids. However, the use of UAE should be paired with green solvents, such as EtOH, which is commonly used in most protocols involving plant material matrices, along with MeOH. Researchers should aim to avoid solvents like hexane, chloroform, acetone, and petroleum ether due to their toxicity and environmental impact. The increasing application of DESs in the extraction of natural products, particularly cannabinoids, shows promise for developing more sustainable methodologies due to their biodegradability, non-toxicity, and recyclability.

To assist researchers in evaluating the environmental impact of their analytical methods, several tools are available in the literature that can be utilized [314,315,316,317,318,319,320]. For example, the Analytical GREEnness calculator translates each of the 12 principles of green analytical chemistry into scores ranging from 0 to 1. Values close to 1, represented by a dark green color, indicate that the evaluated procedure is more environmentally friendly. Additionally, the width of each segment in the resulting pictogram reflects the importance of its corresponding principle [318].

## 7. Materials and Methods

**Figure 4:**
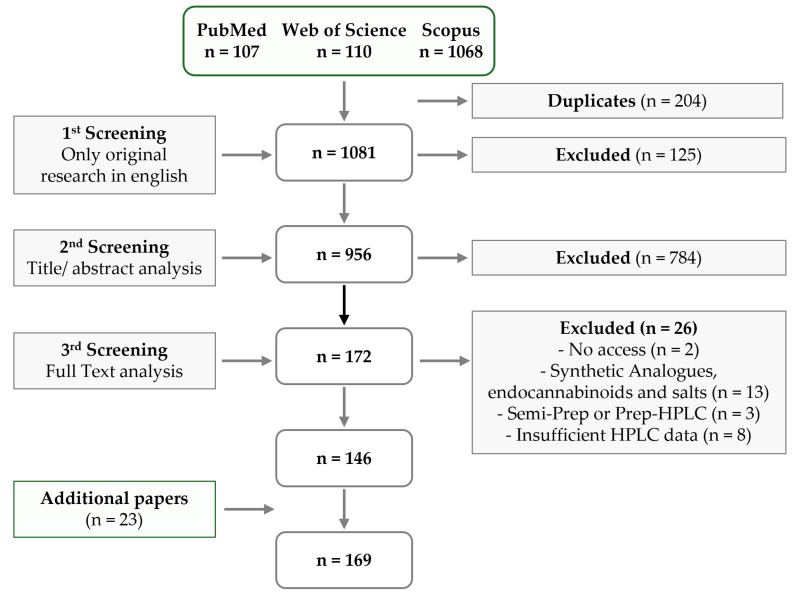
Flow diagram illustrating the review methodology, including the database searches, the number of abstracts analyzed, and the full texts assessed. HPLC: High-Performance Liquid Chromatography; Prep: Preparative.

The literature search was performed up to 6 January 2025, across three online databases: PubMed, Web of Science, and Scopus, to include all indexed papers published from 2022 to 2024. The search terms were categorized into three groups (1st group: “Cannabis”, “THC”, “CBD”, “Hemp”; 2nd group: “HPLC”, “LC”, “UPLC”, UHPLC”, “Chromatography”; 3rd group: “UV”, “DAD”, “PDA”, “Diode”, “Photodiode”, “Ultraviolet”). The search utilized Boolean operators to ensure results included at least one term from each group. When permitted by the search engine, the Boolean operator “Asterisk*” was applied before and after specific terms, such as “*cannabi*” and “*LC”, to capture additional variations of the keywords. For PubMed and Web of Science, the search was performed to include “All Fields” of the database, while in Scopus it was confined to the “Title”, “Abstract”, and “Keywords”. A second, less restrictive search in Scopus (only with terms from the first two groups) was also performed (Figure 4). After removing duplicates, a total of 1081 studies were identified. The initial screening involved excluding papers not written in English and eliminating reviews, book chapters, conference papers, and grey literature. This process resulted in the selection of 956 entries for title and abstract analysis, ultimately narrowing the list down to 172 papers for full evaluation. A study was considered eligible if it was original experimental research that included analytical HPLC-UV analysis of phytocannabinoids, resulting in 146 papers. Upon reviewing the selected papers and relevant review articles, an additional 23 papers were considered eligible to this review, leading to a total of 169 papers (included in the HPLC-UV section, Table 1, Table 2 and Tables S2 and S3).

For the extraction section, a total of 148 manuscripts were analyzed and organized in Table S1 according to the matrices studied and the details of the protocol and extraction techniques employed. Twenty-one manuscripts were excluded from this analysis because important details regarding the extraction method were missing, a dilute-and-shoot protocol was used without actual extraction of cannabinoids from samples, or standards were used instead. Concerning the method-validation section, only papers that reported at least two of the three validation parameters discussed (precision, accuracy, and lower limits) were included in Table S4 (57 papers).

## 8. Conclusions and Future Directions

The growing market for medicinal cannabis and cannabis-based products requires a corresponding enhancement in rigorous quality control, standardization, and innovation to meet regulatory standards and consumer expectations. Reliable quantification of cannabinoids in these diverse cannabis-related products depends on key processes, such as extraction methodologies, sample preparation, and thorough analytical method validation.

In the pharmaceutical industry, the effects of matrix interference on analyte extraction and detection are well-documented, resulting in highly accurate and precise analyses. However, similar research in cannabis analytical testing remains limited, and specific molecular interactions that could impact accuracy are still largely unexplored. Matrix interference in these products is complex, arising from variations in chemovars, differences in cannabinoid extraction procedures, and the effects of food additives and drug formulations on cannabinoid quantification. Furthermore, introducing clean-up steps during extractions can affect extraction efficiency and have varying impacts on structurally distinct cannabinoids. To effectively standardize cannabis analytical methods, scientific efforts must be grounded in comprehensive studies of both the analytes and the matrices involved. Advancements in cannabis testing are essential for the industry to progress beyond the black market and ensure long-term stability.

In the reviewed papers, the most prominent extraction technologies used for cannabinoids have been SFE, UAE, and DES. UAE is the preferred technique across a diverse range of matrices and has been successfully applied to the extraction of cannabinoids from complex sources such as formulated products, plasma, and tissue samples. While DESs show promise for future green extractions, their applicability have thus far been limited to cannabis plants and oil matrices.

Analytical chromatographic separations are still primarily performed using C18 reversed-phase columns. However, in recent years, researchers have begun investigating a variety of stationary phases, including normal phases with NH_2_ adsorbents and polysaccharide-based chiral stationary phases. The analysis of chiral cannabinoids is becoming increasingly important, as different enantiomers and diastereomers may have distinct pharmacological effects.

One of the most significant challenges in the detection and quantification of cannabinoids is the standardization of analytical methods and their associated validation parameters across laboratories to ensure consistent and reliable results on a global scale. To achieve this, the scientific community needs to actively participate in this conversation by reporting in published papers how analytical method validation was performed, specifying how each parameter was calculated, and detailing the protocols involved, including the preparation of quality controls and the determination of matrix effects, analyte recovery, and overall process efficiency. The present review highlights the lack of consistency in the presented validation parameters.

Guidelines should be established for each cannabis-related product to adequately address questions regarding sample preparation, analytical methods, and validation parameters necessary for establishing robust standardized methods, while also investigating the potential for using green techniques, or more environmentally friendly methods.

## Figures and Tables

**Table 1:** Summary of LC parameters used in gradient chromatographic runs.

Entry	Method *^a^*	Mobile Phase *^b^*			Column	Flow Rate (mL/min) *^c^*	Run Time (min) *^d^*	Injection Vol. (µL)	Temp. (°C)	UV Detection (nm) *^e^*	Cannabinoids Analyzed *^f^*	**Ref.**
		Aqueous Phase (A)	Organic Phase (B)	Gradient % B								
1	HPLC-UV/Vis		ACN	10–90	Agilent C18 (150 × 46 mm, 5 μm)	NR	20	10	35	NR	5: CBD, CBDA, CBN, Δ^9^-THC, THCA	[80]
2	HPLC-VWD		ACN	65–75	Restek Raptor ARC-18 (150 × 4.6 mm, 5 μm) with a guard col	1	15 *^d^*	NR	NR	228	6: CBC, CBD, CBG, CBN, Δ^8^-THC, Δ^9^-THC	[81]
3	HPLC-DAD		ACN	70–80	InfinityLab Poroshell 120 C18 (100 × 4.6 mm, 2.7 μm) with a guard col	1	16	20	40	220: CBD, Δ^9^-THC 283: CBC, CBN	4: CBC, CBD, CBG, CBN	[82]
4	HPLC-DAD	0.01% FA	ACN-0.01% FA	65–100	Accucore C18 (100 × 2.1 mm, 2.6 μm)	0.3	12 *^d^*	1	50	230	4: CBD, CBDA, Δ^9^-THC, THCA	[83,84]
5	UHPLC-UV	0.05% FA	ACN-0.05% FA	70–100	Waters Cortecs UPLC C18 (100 × 2.1 mm, 1.6 μm)	0.3	12.5	10	35	228	17: CBC, CBCA, CBD, CBDA, CBDV, CBDVA, CBG, CBGA, CBN, CBNA, CBL, CBLA, Δ^8^-THC, Δ^9^-THC, THCA, THCV, THCVA	[85]
6	HPLC-DAD	0.075% FA	ACN-0.5% FA	70–100	Excel Super C18 (250 × 4.6 mm, 3 μm)	1	14 *^d^*	10	40	210	12: CBC, CBD, CBDA, CBDV, CBDVA, CBG, CBGA, CBN, CBNA, Δ^9^-THC, THCA, THCV	[86]
7	HPLC-DAD	0.1% FA	ACN	65–95	Luna C18 (150 × 4.6 mm, 5 μm) with a guard col	1	28	20	25	210: CBD, Δ^9^-THC 220: CBDA, THCA, CBN	5: CBD, CBDA, CBN, Δ^9^-THC, THCA	[87]
8	HPLC-DAD	0.1% FA	ACN	70–95	InfinityLab Poroshell EC-C18 (150 × 3.0 mm, 2.7 μm) with a guard column	0.5	25	5	35	228	7: CBD, CBDA, CBG, CBGA, CBN, Δ^9^-THC, THCA	[88,89]
9	HPLC-DAD	0.1% FA	ACN-0.1% FA	2–100	Eclipse XDB-C18 (150 × 4.6 mm, 3.5 μm)	0.6	51	10	NR	280	4: CBD, CBDA, CBEA, DHC	[90,91]
10	UHPLC-DAD	0.1% FA	ACN-0.1% FA	5–100	Ascentis Express RP Amide (100 × 2.1 mm, 2.7 μm)	0.4	65	NR	NR	220/269/306: CBDA 220/268/305: CBGA 220/271/304: THCA	3: CBDA, CBGA, THCA *^f^*	[92,93]
11	UHPLC-DAD	0.1% FA	ACN-0.1% FA	5–100	Phenomenex XB-C18 (50 × 2.1 mm, 1.7 μm)	NR	18 *^d^*	NR	NR	210, 273	7: CBC, CBD, CBDV, CBG, CBN, cis-Δ^9^-THC, Δ^9^-THC *^f^*	[94]
12	HPLC-UV/Vis	0.1% FA	ACN-0.1% FA	25–100	µ-Bondapak C18 (300 × 3.9 mm)	2	45	10	NR	225	14: CBC, CBCA, CBD, CBDA, CBDV, CBDVA, CBG, CBGA, CBN, Δ^8^-THC, ∆^9^-THC, THCA, THCV, THCVA	[95]
13	HPLC-DAD	0.1% FA	ACN-0.1% FA	40–90	Zorbax Eclipse XDB C18 (150 × 4.6 mm, 5 μm)	0.7	35	10–15	35	NR	4: CBD, CBDA, CBN, ∆^9^-THC	[96]
14	HPLC-UV *^f^*	0.1% FA	ACN-0.1% FA	60–90	Ascentis Express C18 (150 × 3.0 mm, 2.7 μm)	0.4	40 or 45 *^d^*	3	NR	210: NC 220: AC	7: CBD, CBDA, CBG, CBGA, CBN, Δ^9^-THC, THCA	[97,98]
15	UHPLC-DAD	0.1% FA	ACN-0.1% FA	60–90	Fortis SpeedCore C18-PFP (100 mm × 2.1 mm, 2.6 μm)	0.4	11	3	30	210: 6 NC 225: AC, CBC, CBN	12: CBC, CBD, CBDA, CBCT, CBDV, CBDVA, CBE, CBGA, CBN, Δ^9^-*cis*-THC, Δ^9^-THC, THCA	[99,100]
16	HPLC-DAD	0.1% FA	ACN-0.1% FA	65–95	Poroshell 120 SB-C18, (150 × 4.6 mm, 2.7 μm)	0.5	18	2	NR	214	9: CBC, CBD, CBDA, CBG, CBGA, CBN, Δ^9^-THC, THCA, THCV	[101,102]
17	HPLC-DAD	0.1% FA	ACN-0.1% FA	65–100	Zorbax Eclipse Plus C18 (50 × 2.1 mm, 1.8 μm)	0.4	7.5	2	40	210: CBD, Δ^9^-THC 221: CBDA, THCA	4: CBD, CBDA, Δ^9^-THC, THCA	[103,104]
18	UHPLC-DAD	0.1% FA	ACN-0.1% FA	68–95	InfinityLab Poroshell 120 EC-C18 (150 × 2.1 mm, 2.7 μm) with a guard col	0.5	18	1	30	214	11: CBC, CBD, CBDA, CBDV, CBG, CBGA, CBN, Δ^8^-THC, Δ^9^-THC, THCA, THCV	[105,106]
19	HPLC-DAD	0.1% FA	ACN-0.1% FA	70–77	Phenomenex Luna C18(2) (150 × 4.6 mm, 3 μm) with a guard col	1.2	22.2	10	NR	220	11: CBC, CBCA, CBD, CBDA, CBG, CBGA, CBN, Δ^8^-THC, Δ^9^-THC, THCA, THCV	[107]
20	HPLC-DAD	0.1% FA	ACN-0.1% FA	70–90	Poroshell 120 EC-C18 (150 × 4.6 mm, 2.7 μm)	0.4	60	10	25	220	5: CBD, CBDA, CBN, Δ^9^-THC, THCA	[108]
21	HPLC-DAD	0.1% FA	ACN-0.1% FA	70–90	Supersil ODS2 (250 × 4.6 mm, 5 μm)	1	30	10	35	220	5: CBD, CBDA, CBN, Δ^9^-THC, THCA	[109]
22	UHPLC-UV	0.1% FA	ACN-0.1% FA	70–100	Waters Acquity UPLC C18 (150 × 2.1 mm, 1.7 μm)	0.4	14	NR	30	NR	6: CBD, CBDA, CBGA, CBN, Δ^9^-THC, THCA	[110]
23	HPLC-DAD	0.1% FA	ACN-0.1% FA	70–100	Phenomenex Luna Omega C18 (150 × 2.1 mm × 1.6 μm)	0.4	8	5	40	214, 280 ***^e^***	16: CBC, CBCA, CBD, CBDA, CBDV CBDVA, CBG, CBGA, CBL, CBN, CBNA, Δ^8^-THC, Δ^9^-THC, THCA, THCV, THCVA	[111]
24	HPLC-DAD	0.1% FA	ACN-0.1% FA	70–100	Waters SunFire C18 (150 × 3.0 mm, 3.5 μm) with a guard col	NR	29 *^d^*	2	NR	225	3: CBD, CBG, Δ^9^-THC *^f^*	[112]
25	HPLC-UV/Vis	0.1% FA	ACN-0.1% FA	72–90	Zorbax XDB-C18 (150 mm × 4.6 mm, 3.5 μm)	1.5	12 *^d^*	10	30	228	13: CBC, CBD, CBDA, CBDV, CBDVA, CBG, CBGA, CBL, CBN, Δ^8^-THC, Δ^9^-THC, THCA, THCV *^f^*	[113]
26	HPLC-DAD	0.1% FA	ACN-0.1% FA	80–100	Ultimate LP-C18 (150 × 4.6 mm, 2.5 μm)	1	28	10	NR	228	6: CBD, CBDA, CBG, CBN, Δ^9^-THC, THCA *^f^*	[114]
27	HPLC-DAD	1 mM FA (pH 3.53)	ACN	70–99	Hypersil C18 (150 × 4.6 mm, 3 μm) with a guard col	1	18	10	25	NR	14: CBC, CBCA, CBD, CBDA, CBDV, CBDVA, CBG, CBGA, CBN, Δ^8^-THC, Δ^9^-THC, THCA, THCV, THCVA	[115,116]
28	HPLC-DAD	AF 2 mM–0.1% FA	ACN-0.1% FA	70–90	Ascentis Express C18 (150 × 3.0 mm, 2.7 μm)	0.3	20	6	25	210: NC 220: AC	12: CBC, CBCA, CBCV, CBD, CBDA, CBDV, CBGA, CBGV, CBN, Δ^8^-THC, Δ^9^-THC, THCA *^f^*	[117]
29	HPLC-VWD	AF 2 mM–0.1% FA	ACN-0.1% FA	70–90	Ascentis Express C18 (150 × 3.0 mm, 2.7 μm)	0.2	35	3	NR	210	9: CBC, CBCV, CBD, CBDB, CBDV, CBG, CBGV, Δ^8^-THC, Δ^9^-THC	[118]
30	UHPLC-DAD	AF 5 mM–0.1% FA	ACN-0.1% FA	67–95	Ascentis Express C18 (150 × 3.0 mm, 2.7 μm)	1	8 *^d^*	5	40	228	16: CBC, CBCA, CBD, CBDA, CBDV, CBDVA, CBG, CBGA, CBL, CBN, CBNA, Δ^8^-THC, Δ^9^-THC, THCA, THCV, THCVA	[119]
31	UHPLC-UV	AF 5 mM–0.1% FA	ACN-0.1% FA	70–98	Ascenti Express C18 (150 × 2.1 mm, 2 μm)	0.4	8	25	30	228	17: CBC, CBCA, CBD, CBDA, CBDV, CBDVA, CBG, CBGA, CBL, CBLA, CBN, CBNA, Δ^8^-THC, Δ^9^-THC, THCA, THCV, THCVA	[120]
32	HPLC-DAD	AF 5 mM–0.1% FA	ACN-0.1% FA	75–95	Luna C18(2) (150 × 2 mm, 3 μm) with a guard col	0.25	35	5	RT	220	10: CBC, CBD, CBDA, CBDV, CBG, CBGA, CBN, Δ^9^-THC, THCA, THCVA	[121]
33	HPLC-DAD	AF 10 mM (pH 3.6)	ACN-0.1% FA	70–100	InfinityLab Poroshell 120 EC-C18, (100 × 2.1 mm, 1.9 μm)	0.8	11	1	40	NR	16: CBC, CBCA, CBD, CBDA, CBDV, CBDVA, CBG, CBGA, CBL, CBLA, CBN, CBNA, Δ^9^-THC, THCA, THCV, THCVA	[122]
34	HPLC-DAD	AF 20 mM (pH 3.2)	ACN	60–95	XBridge C18 (150 × 4.6 mm, 3.5 μm)	1	NR	25	25	240	3: CBD, CBN, Δ^9^-THC	[123,124]
35	HPLC-DAD	AF 20 mM (pH 3.6)	ACN-0.1% FA	NR	InfinityLab Poroshell 120 EC-C18 (150 × 4.6 mm, 2.7 μm) with a guard col	1	15 *^d^*	5	40	220	6: CBD, CBDA, CBN, Δ^8^-THC, Δ^9^-THC, THCA	[125,126]
36	UHPLC-DAD	AF 50 mM, 10% ACN (pH 3.75)	ACN-0.1% FA	55–90	Cortecs UPLC Waters C18 (100 × 2.1 mm, 1.6 μm) with a guard col	0.3	10	3	30	270	10: CBC, CBD, CBDA, CBDV, CBG, CBGA, CBN, Δ^8^-THC, Δ^9^-THC, THCA	[127]
37	HPLC-UV	0.085% PA	ACN-0.085% PA	70–95	NexLeaf CBX Potency RP C18 (150 × 4.6 mm, 2.7 μm) with a guard col	1	10	5	35	220	9: CBC, CBD, CBDA, CBDV, CBG CBGA, CBN, Δ^9^-THC, THCA	[128]
38	HPLC-DAD	0.085% PA	ACN-0.085% PA	70–95	NexLeaf CBX for Potency C18 (150 × 4.6 mm, 2.7 μm) with a guard col	1.6	10	5	40	220	11: CBC, CBD, CBDA, CBDV, CBG, CBGA, CBN, Δ^8^-THC, Δ^9^-THC, THCA, THCV *^f^*	[129,130,131]
39	HPLC-UV	0.085% PA	ACN-0.085% PA	70–95	NexLeaf CBX for Potency C18 (150 × 4.6 mm, 2.7 μm) with a guard col	1.6	10	NR	35	220	5: CBD, CBDA, CBN, Δ^9^-THC, THCA	[132]
40	HPLC-MWD	0.085% PA	ACN-0.085% PA	75–95	Phenomenex Kinetex XB-C18 (250 × 4.6 mm × 5 μm) with a guard col	1.6	25	NR	35	220	11: CBC, CBD, CBDA, CBDV, CBG, CBGA, CBN, Δ^8^-THC, Δ^9^-THC, THCA, THCV	[133]
41	HPLC-DAD	0.1% PA	ACN-0.1% PA	55–60	Restek Raptor C18 (150 × 4.6 mm, 2.7 μm)	1.5	27	5	45	220	8: CBD, CBDV, CBG, CBN, Δ^8^-THC, Δ^9^-THC, THCA	[134]
42	HPLC-DAD	0.1% PA	ACN-0.1% PA	60–100	InfinityLab Poroshell 120 EC-C18 (50 × 3.0 mm, 2.7 μm)	NR	19	1	NR	208, 220, 230, and 240	26: CBC, CBCO, CBCV, CBCVA, CBD, CBDA, CBDA-ME, CBDP, CBDV, CBE, CBG, CBGA, CBGQA, CBGV, CBGVA, CBL, CBN, CBT, Δ^8^-THC, Δ^9^-THC, THCA, THCB, THCH, THCP, THCV, THCVA	[135]
43	HPLC-DAD	PA 8.64 g/L	ACN	64–82	InfinityLab Poroshell 120 EC-C18 (150 × 3 mm, 2.7 μm)	0.7	30	10	40	225: NC 306: AC	5: CBD, CBDA, CBN, Δ^9^-THC, THCA	[136,137]
44	HPLC-DAD	PA 8.6 g/L	ACN	64–82	Purospher RP-18 (250 × 4.6 mm, 5 μm) with a guard col	1	25	10	40	225, 306	3: CBD, CBN, Δ^9^-THC *^f^*	[138,139]
45	UHPLC-DAD	PA 85% (pH 2.2)	ACN	68–90	Raptor ARC-18 (150 × 2.1 mm, 2.7 μm)	0.4	13	1	25	228: NC 306: AC	8: CBC, CBD, CBDA, CBDV, CBG, CBGA, Δ^9^-THC, THCA	[140]
46	HPLC-DAD	0.05% AcOH (pH 4.40)	ACN	56–75	Phenomenex Kinetex C18 (150 × 4.6 mm, 2.6 μm) with a guard col	0.8	35	5	25	220	12: CBC, CBD, CBDA, CBDV, CBDVA, CBG, CBGA, CBN, Δ^8^-THC, Δ^9^-THC, THCA, THCV	[141]
47	UHPLC-TUV	0.1% AcOH	ACN	53–95	Cortecs RP C18 (150 × 2.1 mm, 1.6 μm)	0.4	35	2	40	230	3: CBD, CBN, Δ^9^-THC	[142]
48	HPLC-DAD	0.1% FA	MeOH	60–90	Phenomenex Kinetex C18 (100 mm × 2.1 mm, 5.0 μm)	0.5	7	5	14	220	6: CBD, CBDA, CBN, Δ^8^-THC, Δ^9^-THC, THCA	[143]
49	HPLC-DAD	0.1% FA	MeOH-0.05% FA	60–95	Dr Maisch ReproSil XR 120 (50 × 3.5 mm, 3 μm)	1	10	1	40	228	7: CBC, CBD, CBDA, CBG, CBN, Δ^9^-THC, THCA	[144]
50	HPLC-DAD	0.1% FA	MeOH-0.05% FA	60–95	InfinityLab Poroshell 120 EC-C18 (150 × 4.6 mm, 4 μm)	1	77	35	50	210, 230	11: CBC, CBCA, CBD, CBDA, CBDVA, CBG, CBGA, CBN, Δ^9^-THC, THCA, THCVA	[145]
51	HPLC-VWD	0.1% FA	MeOH-0.05% FA	60–95	InfinityLab Poroshell 120 EC-C18, (50 × 3 mm, 2.7 μm)	1	11	5	50	230	5: CBD, CBDA, CBN, Δ^9^-THC, THCA	[146]
52	HPLC-DAD	0.1% FA	MeOH-0.05% FA	60–100	InfinityLab Poroshell 120 EC-C18 (50 × 3.0 mm, 2.7 μm)	NR	12.5	5	NR	220	3: CBD, CBN, Δ^9^-THC	[147]
53	HPLC-VWD	0.1% FA	MeOH-0.05% FA	75–95	InfinityLab Poroshell 120 EC-C18, (150 × 4.6 mm)	1	12.5	5	50	230	3: CBD, CBN, Δ^9^-THC	[148]
54	HPLC-DAD	0.1% FA	MeOH-0.1% FA	62–80	Accucore aQ C18 Polar Endcapped (100 × 2.1 mm, 2.6 μm)	0.45	27	2	50	228	13: CBC, CBCA, CBD, CBDA, CBDV, CBDVA, CBG, CBGA, CBN, Δ^9^-THC, THCA, THCV, THCVA	[149]
55	HPLC-UV	0.1% FA	MeOH	67–95	Accucore aQ C18 Polar Endcapped (100 × 2.1 mm, 2.6 μm)	0.45	23	NR	NR	210	7: CBD, CBDA, CBG, CBGA, CBN, Δ^9^-THC, THCA	[150]
56	HPLC-DAD	50 mM AF (pH 5.19)	MeOH	68–95	C-18 (250 × 4.6 mm, 5.0 μm)	1	30	NR	30	240	5: CBD, CBDA, CBN, Δ^9^-THC, THCA	[151]
57	UHPLC-DAD	0.07% PA	MeOH-0.07% PA	65–95	Shim-pack XR-ODSII C18 (2.2 μm)	1	45	10	50	NR	11: CBC, CBD, CBDA, CBDV, CBG, CBGA, CBN, Δ^8^-THC, Δ^9^-THC, THCA, THCV	[152,153]
58	HPLC-DAD	10 mM AA (pH 5.2)	MeOH	72–92	Phenomenex Kinetex C18 (100 × 4.6 mm, 2.6 μm)	0.7	28	10	30	220	8: CBD, CBDA, CBG, CBGA, CBN, Δ^8^-THC, Δ^9^-THC, THCA	[154]
59	HPLC-DAD	25 mM AA	MeOH	75–95	Thermo Hypersil BDS C18 (150 × 4.6 mm, 5 μm)	1	25	NR	NR	205	5: CBD, CBDA, CBN, Δ^9^-THC, THCA	[155]
60	HPLC-DAD	0.1%TFA	MeOH-0.1% TFA	79–100	Waters Cortecs C18 (150 × 4.6 mm, 2.7 μm	0.7	21 *^d^*	NR	54	226	14: CBC, CBCA, CBD, CBDA, CBDV, CBDVA, CBG, CBGA, CBN, Δ^8^-THC, Δ^9^-THC, THCA, THCV, THCVA	[156]
61	HPLC-DAD	0.015% FA	MeOH:ACN (75:25)	74.5–80.5 *^b^*	Restek Raptor ARC-18 (150 × 2.1 mm, 2.7 μ) with a guard col	0.3, 0.5 ***^c^***	32	3	30	230: 8 NC; 262: CBCA, CBNA; 271: 6 AC; 284: CBC, CBN	18: CBC, CBCA, CBD, CBDA, CBDV, CBDVA, CBG, CBGA, CBL, CBLA, CBN, CBNA, CBT, Δ^8^-THC, Δ^9^-THC, THCA, THCV, THCVA *^f^*	[157]
62	HPLC-DAD	0.1% FA	B: ACN-0.1% FA, C: MeOH ***^a^***	55%B,0%C–0%B,100%C	3 columns: Shim-Pak C18 (75 × 3 mm, 2.2 μm), and 2 Phenomenex Synergy C18 (100 × 3 mm, 2.5 μm) with a guard col	0.3–0.41 ***^c^***	88	5–30	40	absorbance maxima of the analyzed CBs ***^e^***	17: CBC, CBCA, CBD, CBDA, CBDV, CBDVA, CBG, CBGA, CBL, CBLA, CBN, CBNA, Δ^8^-THC, Δ^9^-THC, THCA, THCV, THCVA	[158]
63	HPLC-DAD	0.1% FA	B: ACN-0.1% FA, C: MeOH *^a^*	70%B,0%C– 0%B,100%C	Agilent XDB (250 × 4.6 mm, 5.0 μm)	NR	33 *^d^*	NR	NR	200–400	8: CBC, CBD, CBDA, CBG, CBGA, CBN, Δ^9^-THC, THCA	[159]
64	HPLC-DAD	0.1% PA	B: ACN C: MeOH *^a^*	38%B,33%C–53%B,47%C	Phenomenex C18 (150 × 4.6 mm, 5 μm)	1.2	26.5	10	30	220	6: CBD, CBDA, CBN, CBNA, Δ^9^-THC, THCA	[160,161]
65	HPLC-DAD	AF 20 mM 0.1% FA	B: ACN, C: MeOH 10 mM AF, 0.05% FA *^a^*	50%B,0%C–45%B,45%C	Phenomenex Luna C18(2) (150 × 4.6 mm, 5 μm) with a guard col	2.5	34	10	40	232	10: CBC, CBD, CBDA, CBDV, CBG, CBGA, CBN, Δ^8^-THC, Δ^9^-THC, THCA	[162]

AA: ammonium acetate; AC: acidic cannabinoids; AcOH: acetic acid; ACN: acetonitrile; AF: ammonium formate; Col: column; DAD: diode array detector; FA: formic acid; HPLC: high-performance liquid chromatography; MeOH: methanol; MWD: multiple wavelengths detector; NC: neutral cannabinoids; NR: not reported; PA: phosphoric acid; RT: room temperature; TFA: trifluoroacetic acid; TUV: tunable ultraviolet; UHPLC: ultra high-performance liquid chromatography; UV: ultraviolet; Vis: visible; VWD: variable wavelength detector. Cannabinoids Abbreviations: CBD: cannabidiol; CBDA: cannabidiolic acid; CBDA-ME: cannabidiolic acid methyl ester; CBDP: cannabidiphorol; CBE: cannabielsoin; CBEA: cannabielsoic acid; CBDV: cannabidivarin; CBC: cannabichromene; CBCA: cannabichromenic acid; CBCO: cannabichromeorcin; CBCV: cannabichromevarin; CBCVA: cannabichromevarinic acid; CBG: cannabigerol; CBGA: cannabigerolic acid; CBGV: cannabigerovarin; CBGVA: cannabigerovarinic acid; CBL: cannabicyclol; CBLA: cannabicyclolic acid; CBN: cannabinol; CBT: cannabicitran; Δ^8^-THC: Δ^8^-tetrahydrocannabinol; Δ^9^-THC: Δ^9^-tetrahydrocannabinol; THCA: Δ^9^-tetrahydrocannabinolic acid; THCB: Δ^9^-tetrahydrocannabutol; THCH: Δ^9^-tetrahydrocannabihexol; THCP: Δ^9^-tetrahydrocannabiphorol; THCV: Δ^9^-tetrahydrocannabivarin; THCVA: Δ^9^-tetrahydrocannabivarinic acid. *^a^* Type of liquid chromatography apparatus and UV detector used; when not specified, the UV term is used; Entry 14: HPLC-VWD [98] HPLC-DAD [97]. *^b^* Entry 1–3: Only water was used as aqueous phase; Entry 61: Method used a four-step isocratic mobile phase; Entry 62–65: Method used a ternary solvent system, with two separate organic phases (B and C). *^c^* Entry 61: 0–7 min: F = 0.3 mL/min, 17.5–30 min: F = 0.5 mL/min; Entry 62: 0–17 min: 0.3 mL/min, 19–30 min: 0.35 mL/min, 32–46 min: 0.4 mL/min; 70 min: 0.41 mL/min. *^d^* When reported, run time includes column equilibration; Entry 2, 35: As indicated in the published chromatograms; Entry 4, 6, 11, 24, 25, 30, 60, and 63: No column equilibration was reported; Entry 14: Run time: 45 min [98] or 40 min [97]. *^e^* When reported, the wavelengths described correspond to those selected for quantification. Entry 23: Only CBDA and THCA were quantified by UV at 280 nm; Entry 62: DAD detection (nm): CBC: 230, 279; CBCA: 199, 254; CBD: 205, 274; CBDA: 221, 268; CBDV: 205, 273; CBDVA: 221, 268; CBG: 205, 273; CBGA: 220, 268; CBL: 210, 278; CBLA: 227, 273; CBN: 215, 284; CBNA: 262, 326; Δ^8^-THC: 207, 279; Δ^9^-THC: 209, 279; THCA: 220, 271; THCV: 205, 278; THCVA: 220, 269. *^f^* Additional cannabinoids were assigned by MS: Entry 10, 25, 26, 44, 61; Entry 9: Cannabinoids were identified by MS and semi-quantified by UV; Entry 28: The developed method enabled the separation of 12 out of 14 cannabinoids. The additional two (CBG and CBNR) were assigned by MS. Entry 38: An additional 9 cannabinoid were examined in relation to the separation of Δ^8^-THC, Δ^9^-THC, and THCA [130].

**Table 2:** Summary of LC parameters used in isocratic chromatographic runs.

Entry	Method *^a^*	Mobile Phase *^b^*			Column	Flow Rate (mL/min) *^c^*	Run Time (min) *^d^*	Injection Vol. (µL)	Temp. (°C) *^e^*	UV Detection (nm) *^f^*	Cannabinoids Analyzed *^g^*	**Ref.**
		Aqueous Phase (A)	Organic Phase (B)	Ratio A:B								
66	HPLC-DAD		ACN	20:80	Chromolith RP18 (150 × 4.6 mm, 5 μm)	1	45	NR	RT	254	5: CBD, CBDV, CBN, Δ^9^-THC, THCV	[163]
67	HPLC-DAD		ACN	20:80	Kinetex C18 (150 × 4.6 mm, 5 μm)	1	9	25	35	210	4: CBD, CBN, Δ^9^-THC, THCA	[164]
68	HPLC-UV		ACN	20:80	Kromasil C8 (150 × 4.6 mm, 5 μm)	1	15	20	NR	272	3: CBN, Δ^9^-THC, THCA	[165]
69	HPLC-DAD		ACN	50:50	C18 (250 × 4.6 mm, 5 μm)	1	40	20	NR	228	5: CBC, CBD, CBG, CBN, Δ^9^-THC	[166,167]
70	UHPLC-DAD	0.028% FA (pH 3.6)	ACN	27:73	Luna Omega Polar C18 (150 × 2.1 mm, 1.6 μm) with a guard col	0.3	15	3	30	223, 230, 251, 269, 285 *^f^*	15: CBC, CBCA, CBD, CBDA, CBDV, CBDVA, CBG, CBGA, CBLA, CBN, Δ^8^-THC, Δ^9^-THC, THCA, THCV, THCVA	[168]
71	HPLC-UV	0.1% FA	ACN	16:84	Zobax Eclipse Plus C18	NR	26	5	NR	280	12: CBC, CBD, CBDA, CBDV, CBG, CBGA, CBL, CBN, Δ^8^-THC, Δ^9^-THC, THCA, THCV	[169]
72	HPLC-VWD	0.1% FA	ACN	20:80	Zorbax Eclipse XDB C18 (250 × 3 mm, 5 μm)	0.5	25	NR	30	220	4: CBD, CBDA, Δ^9^-THC, THCA	[170]
73	HPLC-DAD	0.1% FA	ACN	25:75	Mightysil RP-8GP (150 × 2 mm, 5 μm)	0.2	16	5	40	210: CBD, Δ^9^-THC 284: CBDA, CBN, THCA	5: CBD, CBDA, CBN, Δ^9^-THC, THCA	[171]
74	HPLC-DAD	0.1% FA	ACN	25:75	Phenomenex Kinetex C18 (150 × 2.1 mm, 2.6 μm) with guard col	0.5	30	10	20	230	6: CBD, CBDA, CBN, Δ^8^-THC, Δ^9^-THC, THCA	[172]
75	UHPLC-DAD	0.1% FA	ACN	41:59	Cortecs UPLC Shield RP18 (100 × 2.1 mm, 1.6 μm)	0.5	13	2	35	269	4: CBD, CBDA, Δ^9^-THC, THCA	[173]
76	UHPLC-DAD	0.1% FA	ACN	48:52	Cortecs UPLC Shield RP18 (100 × 2.1 mm, 1.6 μm)	0.7	18	5	35	228	4: CBD, CBDA, Δ^9^-THC, THCA	[174,175]
77	UHPLC-DAD	0.1% FA	ACN	49–51	Hypersyl Gold RP (150 × 2.1 mm, 1.9 μm)	0.45	25	5	35	280	5: CBD, CBDA, CBN, Δ^9^-THC, THCA	[176]
78	HPLC-DAD	0.1% FA	ACN-0.1% FA	17.5:82.5	two HALO C18 (100 × 4.6 mm, 2.7 μm) (200 mm total length)	1	12	20	NR	222: CBN 230: CBC 210: other 4 NC	6: CBC, CBD, CBG, CBN, Δ^8^-THC, Δ^9^-THC	[177]
79	HPLC-DAD	0.1% FA	ACN-0.1% FA	23:77	Phenomenex Luna C18(2) (150 × 4.6 mm, 3 μm)	1.2	20	10	NR	220	4: CBD, CBDA, Δ^9^-THC, THCA	[178]
80	HPLC-DAD	0.1% FA	ACN-0.1% FA	25:75	InfinityLab Poroshell 120 EC-18 (150 × 3.0 mm, 2.7 μm)	0.6	12	10	30	228	12: CBC, CBD, CBDA, CBDV, CBG, CBGA, CBL, CBN, Δ^8^-THC, Δ^9^-THC, THCA, THCV	[179]
81	HPLC-DAD	0.1% FA	ACN-0.1% FA	25:75	Phenomenex Luna C18(2) (250 × 4.6 mm, 3 μm) with a guard col	1, 1.2 *^c^*	30	10	40	220	15: CBC, CBCA, CBD, CBDA, CBDV, CBDVA, CBG, CBGA, CBL, CBN, Δ^8^-THC, Δ^9^-THC, THCA, THCV, THCVA	[180]
82	HPLC-UV	0.1% FA	ACN-0.1% FA	30:70	Phenomenex Aeris peptide XB-C18 (250 × 2.1 mm, 2.6 μm)	0.35	20	10	35	235	5: CBD, CBDA, CBN, Δ^9^-THC, THCA	[181]
83	UHPLC-DAD	5 mM FA	ACN	42:58	Zorbax Eclipse Plus C18 (100 × 2.1 mm, 1.8 μm)	0.5	25	8	NR	215	5: CBD, Δ^(4)8^-iso-THC Δ^8^-iso-THC, Δ^8^-THC, Δ^9^-THC	[216]
84	HPLC-DAD	AF 0.5 mM 0.02% FA (pH 3.0)	ACN	25:75	Raptor ARC-18 (150 × 2.1 mm, 2.7 μm) with a guard col	0.4	20	4	30	223, 230, 251, 261, 269, 285 *^f^*	20: CBC, CBCA, CBCV, CBD, CBDA, CBDV, CBDVA, CBG, CBGA, CBL, CBLA, CBN, CBNA, CBT, Δ^8^-THC, Δ^9^-THC, Δ^8^-THCA, THCA, THCV, THCVA	[182]
85	HPLC-VWD	AF 0.5 mM– 0.1% FA	ACN- AF 0.5 mM 0.1% FA	25:75	Restek Raptor ARC18 (150 × 4.6 mm, 2.7 μm)	1.5	9	NR	NR	220	16: CBC, CBCA, CBD, CBDA, CBDV CBDVA, CBG, CBGA, CBL CBN, CBNA, Δ^8^-THC, Δ^9^-THC THCA, THCV, THCVA	[183]
86	HPLC-DAD	AF 2 mM– 0.011% FA (pH 3.6)	ACN	27:73	Luna Omega Polar C18 (150 × 2.1 mm, 1.6 μm) with guard col	0.3	18	3	30	223, 230, 251, 269, 285 *^f^*	15: CBC, CBCA, CBD, CBDA, CBDV, CBDVA, CBG, CBGA, CBLA, CBN, Δ^8^-THC, Δ^9^-THC, THCA, THCV, THCVA	[184]
87	HPLC-UV	AF 5 mM–0.1% FA	ACN-0.1% FA	20:80	ES Industries Harmony Secure RP18 (150 × 4.6 mm, 3.5 μm) with a guard col	1	10	20	40	210, 228	3: CBD, CBN, Δ^9^-THC	[185]
88	HPLC-UV	AF 5 mM–0.1% FA	ACN-0.1% FA	20:80	Harmony Secure RP18 (150 × 4.6 mm, 3.5 μm)	1	15	50	40	210: CBD, Δ^9^-THC, CBN 228: THCA, CBDA	5: CBD, CBDA, CBN, Δ^9^-THC, THCA	[186]
89	HPLC-UV	AF 5 mM–0.1% FA	ACN-0.1% FA	20:80	Shimadzu Nexcol C18 (50 × 3.0 mm, 5 μm)	0.3	8	5	40	228	3: CBD, CBN, Δ^9^-THC	[185]
90	UHPLC-DAD	AF 5 mM–0.1% FA	ACN-0.1% FA	25:75	Raptor ARC-18 (100 × 3.0 mm, 1.8 μm) with a guard col	1	6	2	40	228	15: CBC, CBCA, CBD, CBDA, CBDV, CBDVA, CBG, CBGA, CBL, CBN, Δ^8^-THC, Δ^9^-THC, THCA, THCV, THCVA	[187]
91	HPLC-DAD	AF 10 mM–0.1% FA	ACN-0.1% FA	30:70	Inertsil ODS-HL (150 × 2.1 mm, 3 μm)	0.3	35	NR	40	220	13: CBC, CBD, CBDA, CBDV CBG, CBGA, CBL, CBN, Δ^8^-THC, Δ^9^-THC, THCA, THCV, THCVA	[188]
92	HPLC-DAD	AF 20 mM (pH 3.6)	ACN	25:75	Phenomenex Kinetex C18 (150 × 4.6 mm, 2.6 μm) with a guard col	0.8	15	5	40	220	10: CBC, CBD, CBDA, CBG, CBGA, CBN, Δ^8^-THC, Δ^9^-THC, THCA, THCV	[189,190,191,192]
93	HPLC-DAD	AF 20 mM–FA (pH 2.9)	ACN-0.1% FA	30:70	Phenomenex Kinetex XB-C18 (150 × 2.1 mm, 1.7 μm)	0.3	16	2	NR	228	16: CBC, CBCA, CBD, CBDA, CBDVA, CBG, CBGA, CBL, CBN, CBNA, Δ^8^-THC, Δ^9^-THC, THCA, THCA-C4, THCV, THCVA	[193,194,195,196,197,198,199]
94	HPLC-DAD	AF 0.1 M– 0.1% FA	ACN-AF 0.1 M, 0.1% FA	22.5:77.5	Phenomenex Luna C18 (2) (250 × 3 mm, 3 μm)	0.55	21	8	37	275	17: CBC, CBCA, CBD, CBDA, CBDV, CBDVA, CBG, CBGA, CBL, CBLA, CBN, CBNA, Δ^8^-THC, Δ^9^-THC, THCA, THCV, THCVA	[200,201,202]
95	HPLC-UV	formate buffer	ACN	20:80	CTInstruments C18, (150 × 4.6 mm, 5 μm)	1.2	13.2	70	30	220	4: CBDA, CBD, Δ^9^-THC, THCA	[203]
96	HPLC-DAD	0.1% TFA	ACN	25:75	Cortecs C18 (150 × 4.6 mm, 2.7 μm)	1	15	NR	NR	228	8: CBC, CBD, CBDA, CBDV, CBDVA, CBL, Δ^9^-THC, THCA	[204]
97	UHPLC-DAD	0.1% TFA	ACN	41:59	Cortecs Shield RP18 (150 × 4.6 mm, 2.7 μm)	2	50	10	35	228	8: CBC, CBD, CBDA, CBG, CBGA, CBN, Δ^9^-THC, THCA	[205,206,207]
98	HPLC-DAD	0.1% TFA	ACN	41:59	Cortecs Shield RP18 (150 × 4.6 mm, 2.7 μm)	1	50	20	35	228	9: CBCA, CBD, CBDA, CBG, CBGA, CBN, Δ^8^-THC, Δ^9^-THC THCA	[208]
99	HPLC-DAD	0.1% TFA	ACN	41:59	Reliant C18 (250 × 4.6 mm, 5 μm)	1	20	20	35	235	10: CBC, CBD, CBDA, CBDV, CBG, CBN, Δ^8^-THC, Δ^9^-THC, THCA THCV	[209]
100	HPLC-VWD		MeOH	13:87	MultoHigh 100 RP18 (125 × 4.6 mm, 3 μm)	0.2	40	10	35	212	3: CBD, CBDV, CBN	[210]
101	HPLC-UV		MeOH	17:83	ACE 3 C18-PFP (150 × 3.0 mm, 3 μm) with guard col	0.4	20	5	25	222	3: CBD, CBN, Δ^9^-THC ***^g^***	[211,212]
102	HPLC-DAD	0.1% TFA	MeOH	15:85	Raptor ARC-18 (150 × 2.1 mm, 2.7 μm) with guard col	0.3	10	3	30	230, 269 *^f^*	18: CBC, CBCA, CBD, CBDA, CBDV, CBDVA, CBG, CBGA, CBL, CBLA, CBN, CBNA, CBT, Δ^8^-THC, Δ^9^-THC, THCA, THCV, THCVA	[213]
103	HPLC-DAD	AcOH 0.25%	ACN:MeOH (15:1)	20:80	Phenomenex Luna C18(2) (150 × 4.6 mm, 5.0 μm)	1	30	10	30 or 35 *^e^*	228	7: CBD, CBDA, CBN, Δ^8^-THC, Δ^9^-THC, THCA, THCV	[214,215]
104	HPLC-DAD	1% ACN in hexane		NA	Nucleosil Ag(I) phase (100 × 4.6 mm, 3 μm)	1	40	5	NR	215	5: CBD, Δ^(4)8^-iso-THC Δ^8^-iso-THC, Δ^8^-THC, Δ^9^-THC	[216]

AC: acidic cannabinoids; AcOH: acetic acid; ACN: acetonitrile; AF: ammonium formate; Col: column; DAD: diode array detector; FA: formic acid; HPLC: high-performance liquid chromatography; MeOH: methanol; NA: non-applicable; NC: neutral cannabinoids; NR: not reported;TFA: trifluoroacetic acid; UHPLC: ultra high-performance liquid chromatography; RT: room temperature; UV: ultraviolet; VWD: variable wavelength detector. *Cannabinoids Abbreviations*: CBD: cannabidiol; CBDA: cannabidiolic acid; CBDV: cannabidivarin; CBC: cannabichromene; CBCA: cannabichromenic acid; CBCV: cannabichromevarin; CBG: cannabigerol; CBGA: cannabigerolic acid; CBL: cannabicyclol; CBLA: cannabicyclolic acid; CBN: cannabinol; CBT: cannabicitran; Δ^(4)8^-iso-THC: Δ^4(8)^-iso-tetrahydrocannabinol; Δ^8^-iso-THC: Δ^8^-iso-tetrahydrocannabinol; Δ^8^-THC: Δ^8^-tetrahydrocannabinol; Δ^9^-THC: Δ^9^-tetrahydrocannabinol; THCA: Δ^9^-tetrahydrocannabinolic acid; THCV: Δ^9^-tetrahydrocannabivarin; THCVA: Δ^9^-tetrahydrocannabivarinic acid. *^a^* Type of liquid chromatography apparatus and UV detector used; when not specified, the UV term is used. *^b^* Entry 66–69, 100, and 101: Only water was used as aqueous phase (A). *^c^* Entry 81: 0–10 min: F = 1 mg/mL, 10.1–30 min: F = 1.2 mg/mL. *^d^* When not clearly stated in the paper, run time is indicated as presented in the published chromatograms. *^e^* Entry 103: 30 °C [214], 35 °C [215]. *^f^* When reported, the wavelengths described correspond to those selected for quantification. Entry 70: THCVA was determined at 269 nm and oven temperature of 45 °C. Entry 70, 84, 86: DAD wavelength (nm) used for specific cannabinoids alongside 230: 223 (CBT), 251 (CBCA), 261 (CBNA), 269 (the other AC), 285 (CBN, CBC). Entry 102: THCA: 269 nm. *^g^* Entry 100: Additional cannabinoids were assigned by MS.

## Data Availability

Not applicable.

## Supplementary Materials

The following supporting information can be downloaded at https://www.mdpi.com/article/10.3390/ph18060786/s1, Table S1: Extraction methods for the analysis of cannabinoids in different matrices; Table S2: Summary of LC parameters used in gradient chromatographic runs for detection of 1 or 2 cannabinoids; Table S3: Summary of LC parameters used in isocratic chromatographic runs for detection of 1 or 2 cannabinoids; Table S4: Summary of validation parameters: Linearity, Precision, Accuracy, LOD, and LOQ.

## Abbreviations

The following abbreviations are used in this manuscript:

AC	Acidic cannabinoids
ACN	Acetonitrile
AOAC	Association of Official Analytical Collaboration
C18	Octadecyl
C8	Octyl
CB	Cannabinoid receptor
CBC	Cannabichromene
CBCA	Cannabichromenic acid
CBCO	Cannabichromeorcin
CBCT	Cannabicitran
CBCV	Cannabichromevarin
CBCVA	Cannabichromivarinic acid
CBD	Cannabidiol
CBDA	Cannabidiolic acid
CBDA-ME	Cannabidiolic acid methyl ester
CBDP	Cannabidiphorol
CBDV	Cannabidivarin
CBDVA	Cannabidivarinic acid
CBE	Cannabielsoin
CBEA	Cannabielsoic acid
CBG	Cannabigerol
CBGA	Cannabigerolic acid
CBGQA	Cannabigerol quinone acid
CBGV	Cannabigerovarin
CBGVA	Cannabigerovarinic acid
CBL	Cannabicyclol
CBLA	Cannabicyclolic acid
CBN	Cannabinol
CBNA	Cannabinolic acid
CBT	Cannabitriol
CV	Coefficient of variation
CINV	Chemotherapy-induced nausea and vomiting
DAD	Diode array detector
DES	Deep eutectic solvent
DM	Dynamic maceration
DoE	Design of Experiments
DSLME	Dispersive solid–liquid microextraction
Δ^4(8)^-iso-THC	Δ^4(8)^-iso-tetrahydrocannabinol
Δ^8^-iso-THC	Δ^8^-iso-tetrahydrocannabinol
Δ^8^-THC	∆^8^-tetrahydrocannabinol
Δ^9^-THC	∆^9^-tetrahydrocannabinol
Δ^8^-THCA	∆^8^-tetrahydrocannabinolic acid
DHC	Dihydrocannabinol
ES	Eutectic solvent
EtOH	Ethanol
FA	Formic acid
GC	Gas chromatography
HBA	Hydrogen bond acceptor
HBD	Hydrogen bond donor
HPLC	High-performance liquid chromatography
ICH	International Council of Harmonisation
LC	Liquid chromatography
LOD	Limit of detection
LOQ	Limit of quantification
MeOH	Methanol
MS	Mass spectrometry
MTBE	Methyl *tert*-butyl ether
MWD	Multiple wavelength detector
NC	Neutral cannabinoids
NR	Not reported
PFP	Pentafluorophenyl
QC	Quality Control
RP	Reversed-phase
RSD	Relative standard deviation
RT	Room temperature
scCO_2_	Supercritical carbon dioxide
SE	Soxhlet extraction
SFE	Supercritical fluid extraction
SLE	Solid–liquid extraction
S/N	Signal to noise ratio
SPE	Solid phase extraction
THCA	∆^9^-tetrahydrocannabinolic acid
THCB	∆^9^-tetrahydrocannabutol
THCH	∆^9^-tetrahydrocannabihexol
THCP	∆^9^-tetrahydrocannabiphorol
THCV	∆^9^-Tetrahydrocannabivarin
THCVA	∆^9^-Tetrahydrocannabivarinic acid
TUV	Tunable ultraviolet
UAE	Ultrasound-assisted extraction
US	Ultrasound
US-LLE	Ultrasound-assisted liquid–liquid extraction
US-SLE	Ultrasound-assisted solid–liquid extraction
UV	Ultraviolet
VWD	Variable wavelength detector
