# Supplementary material for: Recent HPLC-UV Approaches for Cannabinoid Analysis: From Extraction to Method Validation and Quantification Compliance
Source: Pharmaceuticals (Basel). 2025 May 24;18(6):786. doi: 10.3390/ph18060786 (PMC12196272; doi:10.3390/ph18060786)
Supplement: Supplementary file 1 [file pharmaceuticals-18-00786-s001.zip › pharmaceuticals-3647500-supplementary.pdf]

Review

Recent HPLC-UV Approaches for Cannabinoid Analysis: From Extraction to Method Validation and Quantification Compliance

Eduarda M P Silva [1,2], Antonella Vitiello [3], Agnese Miro [3], and Carlos J A Ribeiro [1,2,4*]

[1] Associate Laboratory i4HB - Institute for Health and Bioeconomy, University Institute of Health Sciences - CESPU, 4585-116 Gandra, Portugal; eduarda.silva@iucs.cespu.pt
[2] UCIBIO - Applied Molecular Biosciences Unit, Translational Toxicology Research Laboratory, University Institute of Health Sciences (1H-TOXRUN, IUCS-CESPU), 4585-116 Gandra, Portugal.
[3] Department of Pharmacy, University of Naples Federico II, 80131 Naples, Italy; antonella.vitiello3@unina.it (A.V.); agnese.miro@unina.it (A.M.)
[4] Avextra Portugal SA, 7570-003 Grândola, Portugal.
* Correspondence: carlos.ribeiro@avextra.com

Table S1 - Extraction methods for the analysis of cannabinoids in different matrices

**Entry**	Sample type / quantity (g) [a]	Extraction solvent / volume (mL)	Extraction method and protocol	Extraction recovery (%)	HPLC Entry [g]	Ref.
			**Plant material**			
SE1	Fresh / 0.5 and dried / 0.2 inflorescences	EtOH / 4	SLE with shaking in the dark (500 rpm x 15 min), supernatant (1 mL) centrifugation (12,000 rpm x 4 min)	NR	**93**	[1]
SE2	Fresh / 0.5 and dried / 0.1 inflorescences	EtOH / 4	SLE with shaking in the dark (500 rpm x 15 min), supernatant (1 mL) centrifugation (12,000 rpm x 4 min)	NR	**93**	[2,3]
SE3	Inflorescences / 0.5	EtOH / 15	SLE with shaking (15 min), centrifugation (5,000 rpm x 1 min) (repeated 3x)	NR	**43**	[4]
SE4	Inflorescences / 0.5	EtOH / 15	SLE with shaking (15 min), centrifugation (5,000 rpm x 1 min) (repeated 3x)	NR	**43**	[5]
SE 5	Inflorescences 130 / 0.1	EtOH / 4	SLE with shaking in the dark (500 rpm x 15 min), supernatant (1 mL) centrifugation (12,000 rpm x 4 min)	NR	**93**	[6,7]
SE6	Fresh inflorescences / 0.1	EtOH / 4	SLE with shaking in the dark (500 rpm x 15 min), supernatant (1 mL) centrifugation (12,000 rpm x 4 min)	NR	**93**	[8]
SE7	Inflorescences / 1-2	EtOH / 50	SLE with vigorous shaking (30 min) and centrifugation (4,000 rpm x 5 min)	NR	**85**	[9]
SE8	Inflorescences / 0.5	EtOH / 10	SLE in a tube drive system (two glass beads 6 mm diameter). Agitation and grinding (6,000 rpm x 4 min), mixture kept at RT (9 min) and centrifugation (10,000 rpm x 3 min)	NR	**18**	[10]
SE9	Inflorescences / 0.5	EtOH / 20	Vortexing (5 min), horizontal shaking (250 rpm x 10 min) and centrifugation (4,000 rpm x 5 min)	NR	**96**	[11]
SE10	Inflorescences / 0.3	EtOH / 10	DM (RT, 1 h) under constant stirring (300 rpm) (repeated 3x)	NR	**94**	[12]
SE11	Inflorescences / 0.25	EtOH / 10	DM (RT, 15 min) using stirring. Repeated 2x using 10 and 5 mL of extraction solvent	NR	**28**	[13]
SE12	Inflorescences / 0.25	EtOH / 10	DM (RT, 15 min). Repeated 2x using 10 and 5 mL of extraction solvent	NR	**14**	[14]
SE13	Inflorescences / 50	EtOH / 300	DM (RT, 15 min) using stirring. Repeated 3x with additional 200 mL of extraction solvent	NR	**14**	[15]
SE14	Inflorescences / 1	EtOH / 8	US-SLE (5 min) with EtOH (repeated 3x)	CBD: 98.8-101.1; Δ^9-THC: 98.8-102.5	**S20**	[16]
SE15	Inflorescences / 0.1	EtOH / 10	US-SLE (50 °C, 30 min)	NR	**49**	[17]
SE16	Inflorescences (trichomes) / 0.2	EtOH / 10	US-SLE (25 ºC, 15 min)	NR	**S10**	[18]
SE17	Inflorescences (trichomes) / 0.1	EtOH / 20	US-SLE (RT, 30 min), storage in dark room (18 h), vortexing and laid to rest for 10 min before filtration	NR	**54**	[19]
SE18	Inflorescences / NR	EtOH / NR	US-SLE (30 min, ratio of 1:10 cannabis:EtOH, w/v) and centrifugation (RT, 10,000*g* x 15 min)	NR	**5**	[20]
SE19	Inflorescences / 0.5	EtOH / 10	US-SLE (15 min)	NR	**16**	[21]
SE20	Inflorescences / 1	EtOH / 20	US-SLE (10 min). Extracts cleanup with a mixture of activated carbon (C_{18}) and Mg_2SO_4. Sonication (10 min) and centrifugation (5,000 rpm x 10).	NR	**59**	[22]
SE21	Inflorescences / 100	EtOH / 1000	Reflux (80 ºC, 6 h)	NR	**37**	[23]

Entry	Sample type / quantity (g) [a]	Extraction solvent / volume (mL)	Extraction method and protocol	Extraction recovery (%)	HPLC Entry [g]	Ref.
**Plant material**						
SE22	Inflorescences / 1) 60; 2) 30	1) EtOH / 360<br>2) BUT or DME / 500	1) Maceration (48 h)<br>2) Extraction in a Dexso extractor using a gas flow	NR	**6**	[24]
SE23	1) Inflorescences / 50<br>2) Spent inflorescences / 7.5	1) H_2O / 500<br>2) Hex / 75	1) Hydrodistillation (3 h)<br>2) US-SLE (30 min) of the residue obtained after hydrodistillation (repeated 3x)	NR	**25**	[25]
SE24	Inflorescences / 0.5	MeOH / 5	Vortexing (10 s), shaking (50 rpm x 5 min) and centrifugation (1,500 rpm x 1 min) (repeated 2x)	NR	**38**	[26]
SE25	Inflorescences / NR	MeOH / NR	DM with vortexing (15 min)	NR	**90**	[27]
SE26	Inflorescences / 0.1	MeOH / 10	Horizontal shaking (200 oscillations/min x 90 min) in centrifuge tube light protected and centrifugation (3,000*g* x 5 min)	Method LRRFM[d]: Δ^9-THC: 98.38-100.76; THCA: 96.07-101.29<br>Method RRF[d]: Δ^9-THC: 97.55-100.05; THCA: 91.37-99.76	**76**	[28]
SE27	Inflorescences / 0.01	MeOH / 1	US-SLE (5 min) after vortexing (30 s) and centrifugation (13,000 rpm x 5 min). For recovery determinations, 100 μL of IS (100 μg/mL) was added before extraction	CBDA: 71.2-91.8; THCA-A: 68.6-101.1<br>Low THC strain<br>THCA-A: 99.8-115.5	**23**	[29]
SE28	Inflorescences / 0.02	MeOH / 4	US-SLE (10 min) at RT (25 ± 5 ºC), centrifugation (3,000 rpm x 5 min), supernatant transfer and residue reextraction	CBD: 91.3-97.4	**46**	[30]
SE29	Inflorescences / 0.05	MeOH / 5	US-SLE (15 min) and centrifugation (6,000 rpm x 10 min)	NR	**45**	[31]
SE30	Inflorescences / 0.06	MeOH / 10	US-SLE (10 min)	THC: 94.29-103.44	**67**	[32]
SE31	Inflorescences / 0.2	MeOH / 20	US-SLE (10 min), vortexing, stored for stabilization in cold (-20 ºC, 1 h). Centrifugation (4,200 rpm x 5 min)	NR	**31**	[33]
SE32	Inflorescences / 0.25	MeOH / 25	US-SLE (30 min), supernatant (2 mL) centrifugation (13,000 rpm x 10 min). For recovery experiences extraction was performed with 30 μg/mL of ACBD in MeOH	Δ^9-THC: 97.0-99.3	**102**	[34]
SE33	Inflorescences / 0.25	MeOH / 25	US-SLE (30 min), supernatant (2 mL) centrifugation (13,000 rpm x 10 min). For recovery experiences extraction was performed with 30 μg/mL of ACBD in MeOH	ACBD: 84.9-100.8	**61**	[35]
SE34	Inflorescences / 0.1	MeOH / NR	Sonication (10 min) for homogenization and addition of a ACBD solution in MeOH (75 μg/mL) to afford a final sample concentration of 25 mg/mL. US-SLE (20 min) and centrifugation (13,000 rpm x 10 min)	ACBD: 90-108	**86**	[36]
SE35	Inflorescences / 0.1	MeOH / 25	US-SLE (40 min) and centrifugation (3,000 rpm x 5 min)	NR	**22**	[37]
SE36	Inflorescences / 0.05	ACN / 5	US-SLE (20 min) in a sealed glass test tube, vortexing and centrifugation (6,000 rpm x 2 min)	NR	**101**	[38]

Entry	Sample type / quantity (g) [a]	Extraction solvent / volume (mL)	Extraction method and protocol	Extraction recovery (%)	HPLC Entry [g]	Ref.
**Plant material**						
SE37	Inflorescences / 0.204	EtOH or MeOH or ACN or *n*-Hex or IPA or EtOAc or or combination with different polarity index (n= 14)/ 20	US-SLE (40 ºC, 15 min) after vortexing (10 s), sample cooling to RT and centrifugation (1930*g* x 5 min)	NR	**15**	[39,40]
SE38	Inflorescences / 0.2	MeOH:CH_3Cl (9:1, v/v) / 20	US-SLE (30 min), centrifugation (6,000 rpm x 10 min), supernatant transfer and residue reextraction (10 mL)	CBD: 94.4, 91.1; CBDA: 97.5, 93.8; CBN: 87.2, 89.0; Δ^9-THC: 94.0, 95.0; THCA: 90.0, 106.6	**7**	[41]
SE39	Inflorescences / 0.5	MeOH:$CHCl_3$ (9:1, v/v) / 20	DM (RT, 30 min) and sonication (15 min)	NR	**27**	[42]
SE40	Inflorescences / 1	MeOH:$CHCl_3$ (9:1, v/v) / NR	DM (20 min) and filtration	NR	**36**	[43]
SE41	Inflorescences / 0.05	MeOH:$CHCl_3$ (9:1, v/v) / 2	US-SLE (40 min) and centrifugation (10 ºC, 10,000 rpm x 15 min)	NR	**S32**	[44]
SE42	Inflorescences / 0.1	MeOH:$CHCl_3$ (9:1, v/v) / 5	US-SLE (15 min, RT) (repeated 2x)	CBD: 98; CBDA: 115; CBN: 98; CBC: 98; CBG: 97; Δ^9-THC: 94; THCA: 99	**40**	[45]
SE43	Inflorescences / 1	MeOH:$CHCl_3$ (9:1, v/v) / 10	SLE (1 h) with an IS (4-androstene-3,17-dione) solution at 1 mg/mL in extraction solvent	NR	**79**	[46]
SE44	Inflorescences / 0.05 and cannabis-derived extracellular vesicles / 0.005	MeOH:$CHCl_3$ (9:1, v/v) / 1.5	US-SLE (40 min) after vortexing (5 min). Vortexing was used every 20 min. Centrifugation (10,000 rpm x 15 min)	NR	**S32**	[47]
SE45	Inflorescences / 0.05, 0.1, and 0.2	MeOH:*n*-Hex (9:1, v/v) / 10	1) DM with stirring (10 min), centrifugation (2,007*g*) and collection of supernatants. Reextraction 2x (10 and 5 mL)<br>2) US-SLE (10 min), centrifugation (2,007*g*) and collection of supernatants. Reextraction 2x (10 and 5 mL)	CBD[b]: 94.77-99.45; CBDA[b]: 88.32-94.71; CBN[b]: 113.21-116.85; Δ^9-THC[b]: 109.76-119.28; THCA[b]: 76.92-82.07 (used for method validation)	**56**	[48]
SE46	Inflorescences / 5	Olive Oil Ph. Eur. / 50	DM of grinded sample homogenised in olive oil using a Tolotto Gear® extraction (TGE) method or a TGE preceded by a pre-extraction procedure	NR	**51**	[49]
SE47	Inflorescences / 2	Refined olive Oil Ph. Eur. / 20	1) DM (100 ºC, 40 min) using stirring of grinded sample homogenised in olive oil<br>2) US-SLE (ratio of 1:10 cannabis:oil, 20 min) equipped with a 2 mm sonotrode (200 W, 26 kHz)	NR	**39**	[50]

Entry	Sample type / quantity (g) [a]	Extraction solvent / volume (mL)	Extraction method and protocol	Extraction recovery (%)	HPLC Entry [g]	Ref.
			**Plant material**			
SE48	Inflorescences / 5	scCO_2 / 100-300	SFE using extraction vessel (5 mL) with compacted sample, heating (30-50 ºC) without CO_2 and gradual continuous flow of fluid until reaching a pressure of 3,000-5,000 psi. At 100-300 mL of scCO_2 and stabilisation, the extract was collected	NR	**97**	[51]
SE49	Inflorescences / 100	scCO_2 / 250	SFE with a mass flow rate of scCO_2 of 1.4 kg/h at 15 bar and 25 ºC for 30 min	NR	**50**	[52]
SE50	Inflorescences / 1) 3.7-5.1; 2) 5	1) scCO_2 / 100-300<br>2) EtOH / 50	1) SFE with extraction vessel (25 mL) with sample loaded with CO_2 and maintained in static conditions (15 min). Continuous flow of CO_2 to an average value of 0.50 L/min (NTP). EtOH was used as co-solvent in some experiments (10 wt% to CO_2 mass). Extraction finishing at 100 L of CO_2 at NTP<br>2) Manual stirring at RT (40 s)	(CBD+CBDA): 95.4±1.4<br>(THC+THCA): 97.2±0.5	**27**	[53]
SE51	Inflorescences 1) 1; 2) NR	1) scCO_2 / NR<br>2) EtOH / NR	1) SFE with extraction vessel (100 mL) with scCO_2 for 180 min under a pressure of 250 bar at 45 ºC (repeated 2x). Reconstitution of solvent-free extract in MeOH (2 mL), sonication (15 min) and centrifugation (10,000 rpm x 10 min)<br>2) Ratio of 1:10 (w/w) for extraction. Sonication (15 min) and stirring (magnetic stirrer) in a dark room (4 ºC, 24 h). Evaporation using N_2, reconstitution in MeOH (2 mL) and centrifugation (10,00 rpm x 15 min)	NR	**57**	[54]
SE52	Inflorescences / 1) and 2) 5; 3) 10; 4) 2	1) hot and 2) cold H_2O / 100<br>3) EtOH / H_2O (20, 40, and 80% v/v)<br>4) EtOH / 10	1) The extractant was added hot. Heating and boiling (3 min)<br>2) Shaking (rt, 100 rpm x 24 h)<br>3) DM by stirring (RT, 100 rpm x 3 days)<br>4) US-SLE (40 kHz x 30 min)	NR	**4**	[55]
SE53	Inflorescences and leaves / 6.5	scCO_2 / 250	SFE with scCO_2 under a pressure of 2,000 and 6,000 psi and heating at 50 ºC	NR	**97**	[56]
SE54	Inflorescences and leaves / 0.3	EtOH / 10	DM with constant stirring (RT, 300 rpm x 1 h) (repeated 3x)	NR	**94**	[57]
SE55	Inflorescences and leaves / 1) 10; 2) depending on solvent-to-solid ratio	1) EtOH /200 and EtOAc / 200<br>2) EtOH / 750	1) Soxhlet extractions (2 cycles) under vacuum: 1st cycle with EtOH at reflux (38 ºC, 57.4 kPa, 6 h), removal of extract and 2nd cycle with EtOAc at reflux (31 ºC, 57.4 kPa, 6 h)<br>2) Cryogenic extraction using a rotary tumbler operating at different temperatures (-80-20 ºC, 32 rpm x 30 min)	NR	**77**	[58]
SE56	Inflorescences or leaves / 0.04	MeOH (80%) / 2	Vortexing (30 s) and US-SLE (5 min) (repeated 3x)	NR	**S22**	[59]
SE57	Inflorescences and leaves / 0.05	MeOH / 10	US-SLE (30 ºC, 20 min) with a 100 µg/mL solution of IS (4-androstene-3,17-dione) in MeOH (10 mL)	91.6-111.7	**81**	[60]
SE58	Inflorescences and leaves / 0.1	ACN / 1	US-SLE (35 ºC, 30 min) and centrifugation (10,000 rpm x 10 min)	NR	**103**	[61]

Entry	Sample type / quantity (g) [a]	Extraction solvent / volume (mL)	Extraction method and protocol	Extraction recovery (%)	HPLC Entry [g]	Ref.
			**Plant material**			
SE59	Inflorescences and leaves / 0.1	ACN / 1	US-SLE (30 min)	NR	**103**	[62]
SE60	Inflorescences and leaves / 1	pentane / 5	US-SLE (RT, 30 min)	NR	**13**	[63]
SE61	Inflorescences and leaves / 0.1-0.2	H_2O, MeOH or H_2O:MeOH/ 1 or 15	Mixture of sample and extractant was left standing for 15 min, centrifugation (3,000 rpm x 10 s)	NR	**95**	[64]
SE62	Inflorescences, leaves, stems / 0.15	EtOH / 5	DM (60 min) (repeated 3x)	NR	**94**	[65]
SE63	Inflorescences, leaves, stems / 0.5	MeOH:$CHCl_3$ (9:1, v/v) / 5	US-SLE (15 min) and vortexing at the beginning and every 5 min. Centrifugation (4,500 rpm x 15 min)	NR	**55**	[66]
SE64	Inflorescences / 0.4	ACN:MeOH (4:1, v/v) / 25	US-SLE (RT, 30 min, low power)	NR	**65**	[67]
SE65	1) Inflorescences / a) 0.02 b) 100<br>2) Cannabis crude extract / 0.01	1a) MeOH (80%) / 5<br>1b) EtOH / 500<br>2) MeOH / 1	1a) After decarboxylation: US-SLE (25 ºC, 15 min)<br>1b) DM with shaking in an orbital shaker (24 h) to obtain the crude extract<br>2) After decarboxylation: vortexing and dilution	NR	**35**	[68]
SE66	1) Aerial parts (including leaves and stems) / 0.1<br>2) Inflorescences / 0.02	1) H_2O 30% (w/w) KBr / 2 and ES (100 µL)<br>2) EtOH / 40 µL; H_2O 30% (w/w) KBr / 2 and ES (100 µL)	1) DSLME. Vortexing (30 s) and sonication (40 KHz, 25 ºC, 10 min). Vortexing (30 s) and centrifugation (2,200*g* x 5 min). The supernatant was transferred and centrifuged (2,200*g* x 5 min). ES-rich phase (upper layer) was collected<br>2) same as 1) but sample was mixed with EtOH prior to the addition of the KBr aqueous solution and ES	NR	**S04,S05**	[69]
SE67	Inflorescences, stalks / 1) 15; 2) 1; 3) 25	1) EtOH / 400<br>2) EtOH / 40<br>3) $scCO_2$ / NR	1) Soxhlet extraction (78 ºC, 8 h) of sample in a thimble filter<br>2) DM (RT, 15 min) under magnetic stirring (repeated 2x using 40 and 20 mL of extractant)<br>3) SFE (40 ºC, 360 bar), 30 min in static mode interposed between dynamic phases of 15 min (total extraction time: 140 min)	NR	**S06**	[70]
SE68	Inflorescences, EtOH and CO_2 crude extract, distillate, distillation mother liquors, distil-lation bottoms / 0.1	H_2O / 10	DM with vortexing	NR	**42**	[71]
SE69	1) Inflorescences / 20	1) H_2O / 6000	1) Sample allowed to soak (30 min). Hydrodistillation (5 h) to obtain essential oil (EO).	NR	**63**	[72]

Entry	Sample type / quantity (g) [a]	Extraction solvent / volume (mL)	Extraction method and protocol	Extraction recovery (%)	HPLC Entry [g]	Ref.
**Plant material**						
SE69 (cont.)	2) Inflorescences, lyophilized extracts and deterpenated material / 0.2	2) MeOH:H_2O (70:30, v/v) / 25	1a) Aqueous residue was collected and kept at -20 ºC after filtration. Freeze-dried (-54 ºC, 0.05 mbar) to obtain lyophilized extracts. 1b) Plant biomass (deterpenated material) was dried at 60 ºC for 24 h.2) US-SLE (15 min) and centrifugation	NR	**63**	[72]
SE70	Inflorescences, cannabis samples / 0.1	MeOH / NR[e)]	US-SLE (5 min) and vortexing (repeated 4x). Centrifugation (13,000 rpm, 10 min) of supernatant (2 mL)	ACBD: 93.6-106.1	**84**	**[73]**
SE71	Cannabis samples (aerial parts) / 0.1	MeOH / 10	SLE by shaking horizontally (200 oscillations/min, 90 min) light protected and centrifugation (3000*g* x 5 min)	NR	**76**	[74]
SE72	Seeds /0.6	Acetone/H_2O[d)] (1:1, v/v)	Defatted seeds mixture with solvent, vortexing (5 min), US-SLE (45 min) in a cold and dark room, centrifugation (4,800 rpm, 10 min) (repeated 2x)	NR	**9**	[75,76]
SE73	Seeds and biomass / 0.5	MeOH / 3.5 and *n*-Hex / 3.5	Shaking (800 motion/min, 30-40 min) of samples in MeOH spiked with IS (3,5-dimethylphenol 2.8 mg/1 mL MeOH) and centrifugation (20 ºC, 13 000 rpm x 3 min) (repeated 2x). Addition of *n*-Hex to residue, sonication (30-40 min, 20 ºC) and centrifugation (20 ºC, 13,000 rpm x 3 min) (repeated 2x)	NR	**62**	[77]
SE74	Leaves / 25	EtOH (70%) / 250	Reflux (80 ºC, 2 h) (repeated 2x) and evaporation under vacuum (rotovap). Further extractions were performed in the residue using petroleum ether, EtOAc, and *n*-BuOH	NR	**S08,S26**	[78]
SE75	Leaves / NR	EtOH / NR	Reflux (80 ºC) (repeated 2x). The ratio of dried leaves to solvent was 1:15 by weight	NR	**92**	[79]
SE76	Leaves / 1	MeOH, EtOH, IPA, (1:1, v/v) of MeOH:EtOH, EtOH:IPA, MeOH:IPA	1) DM with shaking (RT, 300 rpm x 24 h)<br>2) US-SLE (40 ºC, 1 h)	NR	**97**	[80]
SE77	Leaves / 20	H_2O / NR	Blended sample was infused in boiling H_2O and allowed to stand for 2 h	NR	**S03**	[81]
SE78	Leaves / 4,000	DCM:MeOH (1:1, v/v) / NR	Extraction at RT, 72 h (method NR)	NR	**66**	[82]
SE79	Leaves and stems / 0.1	MeOH or Acetone / 5	US-SLE (10 min) (repeated 3x)	NR	**10**	[83]
SE80	Leaves and vegetative shoots / 0.15	ACN / 1	US-SLE (1 h), incubation at 55 ºC (1 h) and centrifugation (13,000*g* x 10 min)	NR	**12**	[84]
SE81	Cannabis samples (inflorescences, leaf, stem, and root) / 1	MeOH:$CHCl_3$ (9:1, v/v) / 10	US-SLE (30 min) and centrifugation (5,000 rpm x 5 min)	NR	**8**	[85]
SE82	Cannabis samples / 0.5	MeOH / 20	Vortexing (10 s) and shaking (50 rpm x 30 min), centrifugation (1,000 rpm x 5 min) (repeated 2x)	NR	**38**	[86]

Entry	Sample type / quantity (g) [a]	Extraction solvent / volume (mL)	Extraction method and protocol	Extraction recovery (%)	HPLC Entry [g]	Ref.
			**Plant material**			
SE83	Cannabis samples / 0.1	MeOH / 0.3	Vortexing (3 min)	NR	**26**	[87]
SE84	Cannabis sample / 0.05	MeOH / 1.5	US-SLE (15 min) and centrifugation (12100*g* x 7 min) (repeated 4x)	NR	**32**	[88]
SE85	Cannabis samples / NR	EtOH / NR	US-SLE (15 min)	NR	**16**	[89]
SE86	Cannabis samples / 258.6	Petroleum ether / 1000	US-SLE (3 h) (repeated 3x). Filtrate crude is further extracted using an acid-base extraction	NR	**92**	[90]
SE87	Cannabis samples / 50	EtOH / 200 or 250 g (75% m/m)	Stirring (2 h)	NR	**24**	[91]
SE88	Cannabis samples / 50	Hex or EtOAc / 1000	Maceration (RT, 72 h)	NR	**68**	[92]
SE89	Cannabis samples / 1	EtOH:Hex (1:1, v/v) / 10	US-SLE (probe nº 6, 7 min) in ice bath and centrifugation (400 rpm x 20 min)	CBD: 83.43-112.94; CBDA: 77.88-101.52; CBN: NR; CBC: 77.89-97.77; CBG: 81.42-121.41; CBGA: 80.00-108.62; CBDV: 84.38-113.51; CBL: NR; Δ^8-THC: 77.42-110.99; Δ^9-THC: 73.78-88.67; THCA: 75.89-93.06; THCV: 83.12-112.93	**71**	[93]
SE90	Cannabis samples / 5	1) EtOH / 100<br>2) MeOH:$CHCl_3$ (9:1, v/v) / 100	Turbo-extraction (25 ºC, 4,000 rpm x 2 min) (repeated 2x)	CBD: 91.2-106.7	**48**	[94]
SE91	Cannabis samples / 0.05	EtOH:ACN (1:1, v/v) / 10	Samples spiked with IS (phemprocoumon, 50 µg/mL). US-SLE of powder (1 min, 40 kHz, 180 W) using the solvent extraction	98.8-105.6[c]	**74**	[95]
SE92	Cannabis samples / 0.1	MeOH:$CHCl_3$ (9:1, v/v) / 1	Vortexing (10 s) and US-SLE (15 min with shaking every 5 min) and centrifugation	NR	**58**	[96]
SE93	Cannabis samples / 0.05	MeOH:$CHCl_3$ (9:1, v/v) / 10	US-SLE (30 ºC, 20 min) with IS solution (4-androstene-3,17-dione, 100 µg/mL)	NR	**19**	[97]
SE94	Cannabis samples / 0.1	H_2O / 2 and ES (100 µL)	DSLME. Vortexing (30 s) and sonication (40 KHz, 25 ºC, 10 min). Vortexing (30 s) and centrifugation (4,000 x 5 min). The ES-rich phase (upper layer) was transferred and centrifuged (4,000 rpm x 5 min)	NR	**10**	[98]

**Entry**	Sample type / quantity (g) [a]	Extraction solvent / volume (mL)	Extraction method and protocol	Extraction recovery (%)	HPLC Entry [g]	Ref.
			**Plant material**			
SE95	Cannabis samples / 1) and 2) 5; 3) 60	1) EtOH, petroleum ether (light and heavy), hexane<br>2) vegetable oils (virgin coconut, sacha inchi, perilla seed, sesame seed, olive and rice bran)<br>3) scCO_2 / NR	1) and 2) Maceration (24 h, powder to solvent/oils ratio 1:10) and filtration<br>3) SFE (225 bar, 55 ºC, 4 h)	NR	**8**	[99]
SE96	Cannabis samples/ 0.5	EtOH / 10	Agitation and grinding in a tube dry system of sample and extractant mixture (3,500 rpm x 4 min). Mixture left to stand at RT for 9 min and centrifugation (3,900 rpm x 3 min)	NR	**18**	[100]
SE97	Callus cell (calli dried powder) / 0.3	MeOH / 12	US-SLE (37 ºC, 20 min) and centrifugation	NR	**69**	[101]
			**Oils and resins**			
SE98	Oils / 0.02	*n*-Hex:H_2O (1:1, v/v) / 2	US-LLE. Partitioning with a mixture of extraction solvent spiked and IS (benzo[*ghi*]perylene acetone solution, 1,120 mg/L), vortexing, sonication (15 min) and centrifugation (1,157*g* x 15 min)	NR	**20**	[102]
SE99	Oils / 1) 10; 2) 20	1) MeOH or EtOH or MeOH:H_2O or EtOH:H_2O (8:2, v/v) / 10<br>2) MeOH / 20	1) Classical LLE by shaking (RT, 1.5 h). Repeated 2x<br>2) SPE. Washing of the column with MeOH (5 mL) and *n*-Hex (5 mL). Oil and *n*-Hex (20 mL) mixture was loaded into column. Washing 3x with *n*-Hex (2.5 mL) and extraction with MeOH	NR	**S02**	[103]
SE100	Oils / 1 mL	ACN / 100	Sonication (15 min)	CBD: 1.57-149.18	**17**	[104]
SE101	Oils / 5x10^{-6}	ACN:MeOH (4:1, v/v) / 25	Oil dilution in CH_2Cl_2 (1 mL) and extraction solvent (total of 25 mL). US-LLE (10 min, low power)	NR	**65**	[67]
SE102	Oils, resins / 1	EtOH / 100 (resin) and 20 (oil)	Vortexing (10 min) and centrifugation (5,000 rpm x 10). Extracts cleanup with a mixture of activated carbon (C_{18}) and Mg_2SO_4. Sonication (10 min) and centrifugation (5,000 rpm x 10)	NR	**59**	[22]
SE103	Seed oil / 0.5	MeOH / 25	Vortex (10 s) and sonication (15 min)	NR	**35**	[105]
SE104	Seed oil / 100 μL	IPA / 0.4	Sonication (10 min)	NR	**30**	[106]
SE105	CBD full-spectrum oils / 1	EtOH / 20	Stirring (30 ºC, 120 min) and centrifugation (4,000 rpm x 10 min)	NR	**100**	[107]
SE106	CBD oils / 1.455	*n*-Hex:EtOAc:ACN:MeOH (8:2:8:2, v/v) / NR	LLE by partitioning in a biphasic solvent system	> 90	**11**	[108]
SE107	CBD oils (internet purchase) / 300 μL	MeOH / 10	Sonication (RT, 10 min)	NR	**S11**	[109]

Entry	Sample type / quantity (g) [a]	Extraction solvent / volume (mL)	Extraction method and protocol	Extraction recovery (%)	HPLC Entry [g]	Ref.
			**Oils and resins**			
SE108	Commercial CBD oil / 0.1	H_2O 30% (w/w) KBr / 1 and ES (100 µL)	DSLME. Vortexing (30 s) and sonication (40 KHz, 25 ºC, 10 min). Vortexing (30 s) and centrifugation (2,200g x 5 min). ES-rich phase was collected and diluted with EtOH	NR	**S04,S05**	[69]
SE109	1) Oil / 80 µL<br>2) Resins / 0.001	1) DCM;ACN:MeOH (2:2.2:5.8, v/v) / 10<br>2) MeOH / 10	Sonication (RT, 15 min)	CBD: 99; CBDA: 97; CBN: 99; CBC: 100; CBG: 80; Δ⁹-THC: 96; THCA: 103 (for resins)	**40**	[45]
SE110	Resins / 0.05	MeOH / 5	Sonication (15 min)	NR	**1**	[110]
SE111	Resins / 0.5	EtOH / 10	SLE by agitation and grinding in a tube dry system of sample and extractant mixture (3,500 rpm x 4 min). Mixture left to stand at RT for 9 min and centrifugation (3,900 rpm x 3 min)	NR	**18**	[100]
SE112	Resins / 0.25	MeOH:$CHCl_3$ (9:1, v/v) / 10	Vortexing (1 min), US-SLE (15 min) and centrifugation (3,000 rpm x 5 min)	NR	**99**	[111]
SE113	Resins / 0.05	MeOH:$CHCl_3$ (8:2, v/v) / 10	Mixture with extraction solvent, vortexing (1 min). US-SLE (15 min), mechanical agitation (30 min) and centrifugation (1,370*g* x 1 min)	NR	**64**	[112]
SE114	Resins / 600	scCO_2 / NR	SFE with extraction vessel (5 L, 18 MPa, 40 ºC) and collection of extract after 1 h	NR	**101**	[113]
SE115	Resins (hemp concentrate) / 0.1	MeOH / NR[f]	US-SLE (20 min) and centrifugation (13,000 rpm, 10 min)	ACBD: 94.8-120.5	**70**	[114]
SE116	1) Concentrates (resins, rosins, distillates) / NR<br>2) Infused products / NR	EtOAc and 10% FA in dH_2O / NR	1) DM by homogenization in a Geno/Grinder (1350 cycles/s, 15 min) and centrifugation<br>2) DM by vortexing (2,000 rpm x 15 min) and centrifugation	NR	**90**	[27]
			**Food products**			
SE1117	Infused ice cream/ 3	$CHCl_3$/H_2O (1:3, v/v) / 4	Vortexing (25 s), UAE (20 min), and centrifugation (6,000 rpm x 5 min) (repeated 3x)	CBD: 86.8-92.2; Δ⁹-THC: 84.0-94.0	**S20**	[16]
SE118	CBD edible products	MeOH / NR or another suitable organic solvents /NR	NR	NR	**33**	[115]
SE119	Proteic flour / 15	*n*-Hex / 400	4) Soxhlet extraction. NR	NR	**S06**	[70]
SE120	Flour, flour by-product (coarse residue of the sieving process) / 1	*n*-Hex / 1 and MeOH:H_2O (8:2, v/v) / 1	Sample was homogenized with *n*-Hex and second solvent added. Agitation (5 min), US-SLE (5 min) and centrifugation (3,000 rpm x 10 min). The MeOH/H_2O phase was collected and the residue reextracted 2x more. The methanolic aqueous phase were combined and washed with *n*-Hex (1 mL)	NR	**30**	[106]

Entry	Sample type / quantity (g) [a]	Extraction solvent / volume (mL)	Extraction method and protocol	Extraction recovery (%)	HPLC Entry [g]	Ref.
SE121	1) Water-based cannabis products / 0.5<br>2) Oil-based products / 0.125	1) MeOH / 25<br>2) *n*-Hex / 10	1) Centrifugation (15 min) of sample in MeOH and US-LLE (15 min)<br>2) Centrifugation (15 min) of sample in *n*-Hex and US-LLE (15 min). Solvent removal by pressurized air (vent hood) and residue reconstitution in MeOH (10 mL), sonication (15 min) and centrifugation (15 min)	CBD: 95-101; Δ^9-THC: 93	**88**	[116]
SE122	Tea products / 0.2	1) ACN / 50 and 100<br>2) H_2O / NR	1) Teabags content US-SLE (10 min)<br>2) Teabags added to boiling deionized H_2O and left to infuse (5 min)	NR	**17**	[117]
SE123	CBD infused pomegranate juice/NR	MeOH / NR	10-fold dilution of juice (CBD concentration of 3.5 and 7 mg/L) in solvent, vortexing and centrifugation (4 ºC, 1,700*g* x 15 min)	NR	**34**	[118]
SE124	1) Solid products / 0.08-0.09<br>2) Aqueous products / NR	1) EtOAc / 1<br>2) EtOAc / NR	1) US-SLE (15 min) in ice bath, centrifugation (12,000 rpm x 3 min)<br>2) Extraction with an equal volume of solvent	NR	**53**	[119]
SE125	THC in extra virgin olive oil / 0.3 mL	MeOH / 0.6	Extractant with IS (2 drops, ibuprofen 30 μg/mL in MeOH) added to sample, vortexing (30 s), US-LLE (20 min) and centrifugation (6,000 rpm x 15 min).	NR	**S29**	[120]
SE126	1) Liquid samples (oily samples, e-liquids, gels, honey) / NR<br>2) Gummy bears, candies / NR<br>3) Cookies, chewing gum, candy bars / 0.05<br>4) Tea beverages / NR	1) EtOAc, ACN, ACN:H_2O (1:1, v/v), MeOH:H_2O (1:1, v/v) / NR<br>2) MeOH:H_2O (1:1, v/v) / NR<br>3) ACN:FA (98:2, v/v) / 1.5<br>4) H_2O / 200	1) Dissolution and homogenization<br>2) US-SLE<br>3) US-SLE (15 min) and centrifugation (12100*g* x 7 min) (repeated 4x)<br>4) Infusing of one bag of tea (2x) in boiling for H_2O (5 min)	NR	**32**	[88]
SE127	Δ^8-THC gummies / 1	MTBE:H_2O (1:1, v/v) / 200	US-LLE in an ice bath (40 min). Phase separation (10 min) and analysis of MTBE phase after filtration	NR	**83**	[121]
SE128	1) Chocolate, honey and gummies / NR<br>2) Drinks (e.g. CBD shots) / 10 mL<br>3) CBD-sachets coffee, capsules / 0.5	1) H_2O / 50 and ACN / 50<br>2) and 3) ACN / 100	1) US-SLE. Dissolution in H_2O and sonication (15 min), addition of ACN and sonication (15 min)<br>2) US-LLE (15 min)<br>3) US-SLE (45 min)	CBD: 4.22-87.15	**17**	[104]

Entry	Sample type / quantity (g) [a]	Extraction solvent / volume (mL)	Extraction method and protocol	Extraction recovery (%)	HPLC Entry [g]	Ref.
			**Food products**			
SE129	1) Aqueous tinctures, oils, e-liquids / NR<br>2) Drinks / NR	1) IPA:MeOH (1:1, m/m) or IPA / NR<br>2) MeOH / NR	1) dilution with extractant<br>2) dilution and US-LLE	NR	**S23**	[122]
SE130	Chocolate, cookie, cookie dough, and gummy solid / 0.1	ACN plus FA (2%) / 1	Crushing (5 min) of sample mixture with cold extractant using steel beads and centrifugation (13,400 x 5 min). Vortexing of supernatant (2 mL) mixed with H_2O (0.5 mL) and filtration through Captiva EMR-Lipid cartridge	NR	**91**	[123]
SE131	1) Liquid edibles (sparkling water, tea, SCD probiotics + CBD, water soluble CBG oil, CBNight water soluble CBN oil, water soluble full spectrum hemp oil, or terpene rich hemp oil tincture / 0.1<br>2) CBD gummies, CBD hard candy, and CBD coffee / 1<br>3) CBD brownies, CBD cookies, CBD krispy cereals, marshmallow treats, CBD popcorn, cat food kitty nugs, CBD horse treats, soft CBD dog treats / 1<br>4) CBD tablets / 0.1	1) and 4) MeOH / NR<br>2) MeOH:H_2O (95:5, v/v) / NR<br>3) MeOH / 4	1) and 4) Sonication (10 min) for homogenization and addition of a ACBD solution in MeOH (75 µg/mL) to afford a final sample concentration of 25 mg/mL. US-SLE (20 min) and centrifugation (13,000 rpm x 10 min)<br>2) Preparation of a H_2O-dispersion solution by shaking (3,000 rpm x 5 min) of sample (1 g) in H_2O (4 mL). Addition of H_2O-dispersion solution (200 µL) to a mixture of MeOH (1.8 mL) and ACBD solution in MeOH (2 mL, 75 µg/mL). Sonication (20 min) and centrifugation (13,000 rpm x 10 min)<br>3) Shaking (3,000 rpm x 5 min) of sample (1 g) in ACBD solution in MeOH (4 mL, 0.25 mg/mL) and sonication (20 min). Repeated 2x and centrifugation (13,000 rpm x 10 min)	ACBD: 90-108	**86**	[36]

Entry	Sample type / quantity (g) [a]	Extraction solvent / volume (mL)	Extraction method and protocol	Extraction recovery (%)	HPLC Entry [g]	Ref.
			**Pharmaceutical e other formulated products**			
SE132	Commercial veterinary CBD oil / 100 μL	MeOH / 10	US-LLE (30 ºC, 5 min) with MeOH (5 mL) and dilution to the final volume	98-102	**80**	[124]
SE133	Suk-SaiYas pills / 0.05	MeOH / 5	US-SLE (30 min)	NR	**S01**	[125]
SE134	Commercial Suk-Saiyasna herbal remedy / 30	CH_2Cl_2 / 300	US-SLE (30 min) (repeated 4x)	CBD: and Δ^9-THC: 99.9	**S16**	[126]
SE135	Herbal and cosmetic creams / 0.5	ACN / 20	Agitation (15 min) of homogenous powdered sample mixture with ACN and IS (1 mg/mL), centrifugation (2,579*g* x 15 min, 4 ºC)	CBD: 69.10-98.82; CDBA: 73.46-92.80; CBN: 61.37-96.76; CBCA: 67.10-86.35; CBG: 75.50-99.47; CBGA: 64.39-95.35; Δ^8-THC: 62.54-89.85; Δ^9-THC: 74.15-90.90; THCA: 76.85-86.48	**98**	[127]
SE136	CBD topical products	MeOH / NR or another suitable organic solvents /NR	NR	NR	**33**	[115]
SE137	Water-based CBD lotions / 0.0001	MeOH / 25	Shaking (15 s) of sample dissolved in MeOH, centrifugation (15 min) and US-LLE (RT, 15 min)	NR	**87,89**	[128]
SE138	Ointment cannabis leaf-based / 0.15	IPA / 5	US-SLE (15 min), kept at -20 ºC for 30 min and centrifugation (4 ºC, 10 min)	93.7-104	**92**	[129]
SE139	CBG emulgel (oil-in-gel emulsion) in 1) tapes and 2) membranes /	1) MeOH / 1) 10; 2) 30	US-LLE (RT, 1 h)	NR	**S12**	[130]
SE140	CBD adhesive bandages	MeOH and EtOAc / 25	US-SLE (15 min) of bandages small pieces	NR	**32**	[88]

Entry	Sample type / quantity (g) [a]	Extraction solvent / volume (mL)	Extraction method and protocol	Extraction recovery (%)	HPLC Entry [g]	Ref.
			**Pharmaceutical and other formulated products**			
SE141	Topicals (e.g. face mask, CBD-lip balm) / 0.5	ACN / 100	US-SLE (45 min)	CBD: 1.54-48.15	**17**	[104]
SE142	CBD inhalable powders / 0.001-0.002	PBS (pH 7.4) plus 0.5% sodium lauryl sulfate / 350	Stirring (37 ºC, 100 rpm, withdraw of aliquots at predetermine times, max 90 min) and centrifugation (37 ºC, 4255*g* x 10 min)	CBD: 103.2	**S34**	[131]
SE143	DPCC-CBD inhalable powder / 0.005	PBS (pH 7.4) / 50	Stirring (37 ºC, 100 rpm, 32 h) and centrifugation (37 ºC, 4255*g* x 10 min)	CBD: 100-110	**S34**	[132]
SE144	Tablets (oil-based products) / 0.005	ACN / 35	Dissolution with ACN of grinded and weighed sample (five tablets minimum). US-SLE (15 min) with intermitted shaking	CBD: 105.2; Δ^9-THC: 100.2	**S15**	[133]
SE145	CBD sublingual oil formulations / 0.5	MeOH:$CHCl_3$ (9:1) / 10	US-LLE (30 min) and allowed to stand frozen for 90 min before filtration	NR	**S31**	[134]
SE146	CBD nanoemulsion formulations / NR	ACN / NR	After digestion assay: disruption of nanoemulsion with ACN (ratio of 1:10) in separated mixed micelles and sediment phases. Centrifugation (1,613*g* x 5 min)	NR	**S27**	[135]
SE147	CBD nanoemulsion formulation / 0.001	MeOH / 1	US-LLE	NR	**S19**	[136]
SE148	CBD-loaded organosilica in 1) Cotton swabs, tapes / NR 2) Skin samples / NR	EtOH / NR	1) Shaking in a rotary mixer (RT, 12 h) of samples in EtOH 2) US-SLE (1 h)	NR	**S24**	[137]
SE149	CBD-loaded NP / 300 µL	EtOH / 300 µL	After gastric- and intestine-like release tests, the CBD nanoparticle suspension aliquots obtained at different time points were extracted with EtOH	NR	**S25**	[138]
SE150	CBD-loaded NP / 0.015	DCM	Mixture of nanoparticles in extractant solvent	NR	**S14**	[139]
SE151	Cannabis oil nanoemulsion / 0.05	MeOH / 10	US-SLE (15 min) and centrifugation (4,500*g* x 5 min)	NR	**47**	[140]
SE152	CBD-loaded nanostructured lipid carriers / NR	MeOH / NR	US-SLE (15 min)	NR	**S30**	[141]
SE153	CBD/HP-β-CD inclusion complex / NR	ACN:NH_4HCO_2 (75:25, v/v, 20 mM, pH 3.6) / NR	Dissolution in mobile phase and US-LLE (RT, 40 min)	NR	**92**	[142]

Entry	Sample type / quantity (g) [a]	Extraction solvent / volume (mL)	Extraction method and protocol	Extraction recovery (%)	HPLC Entry [g]	Ref.
**Pharmaceutical and other formulated products**						
SE154	e-Cigarettes semiliquid / 0.025	MeOH / 10	US-LLE (20 min, 30 ºC)	91.6-111.7	**81**	[60]
SE155	CBD e-cigarettes liquid / NR	ACN:H_2O (1:1, v/v) / NR	Dilute-and-shoot approach (1:50)	NR	**75**	[143]
SE156	Cigarette material / 0.1	MeOH / NR[e)]	US-SLE (5 min) and vortexing (repeated 4x). Centrifugation (13,000 rpm, 10 min) of supernatant (2 mL)	ACBD: 93.6-106.1	**84**	[73]
SE157	Smoking product/ 0.1	MeOH / 10	SLE by shaking horizontally (200 oscillations/min, 90 min) light protected and centrifugation (3000*g* x 5 min)	NR	**76**	[74]
**Biological materials**						
SE158	Human and mouse plasma, mouse lung and liver / 100 µL mouse brain tissue / 200 µL	EtOAc / 500 µL	Addition of IS to sample, Simvastatin (50 µg/mL in MeOH) for human plasma and Perampanel (60 µg/mL in MeOH) for mouse matrices. Protein precipitation with IPA (150 µL) and vortex (60 s). LLE with vortexing-mixing (60 s), centrifugation (12,100*g* x 3 min) (repeated 2x)	CBD: 80.12-91.71; CBN: 86.81-88.63; CBC: 86.60-94.99; CBG: 77.31-101.89	**3**	[144]
SE159	Human plasma / 1 mL	NR	An aliquot of each sample (1 mL) from the dinding assay was added to an micropartition system (MW cutoff 30,0000). Centrifugation (2,000 rpm x 30 min) and unbonded CBD determination in ultrafiltrate assayed	NR	**S33**	[145]
SE160	1) CBD nanosuspension in rat skin / NR<br>2) Rat plasma / 100 µL	1) MeOH / 3<br>2) MeOH:ACN (1:1, v/v) / 0.2	1) Agitation in a shaker (24 h), sonication (30 min) (repeated 2x)<br>2) Sample mixture with IS (10 µL of Δ⁹-THC-D3, 1 µg/mL), vortex (1 min), addition of extractant. Vortexing (1 min) and centrifug ation (10,000 rpm x 10 min)	NR	**S17**	[146]
SE161	1) CBD-polymer coated NP in agarose patch / NR<br>2) CBD-polymer coated NP in rat brain tissue / NR	1) EtOH / 2<br>2) Hex / 3	1) Sonication (30 min) of sample and EtOH (1 mL). Aspiration of solvent (700 µL) and repeat procedure after addition of EtOH (1 mL)<br>2) Protein precipitation followed by LLE. Vortex (5 min) and centrifugation (4,000*g* x 10 min)	NR	**S18**	[147]
SE162	Zebrafish larvae tissue / 12 specimens	MeOH + 0.1% FA / 200 µL	SLE by bead beating (4-8 ºC, 3 min, 1 mm glass beads), centrifugation of homogenate (18,000*g* x 30 min)	NR	**78**	[148]

a) Quantities reported in grams (g) or otherwise clearly stated; b) Cannabinoid extract in MeOH prepared by SFE was used as secondary reference standards; c) No specification to which cannabinoids the values are referred too; d) Linear Relative Response Factors (LRRFM) and Relative Response Factors (RRF) models used to quantify low concentrations of Δ⁹-THC and THCA in cannabis flowers using CBD as reference; e) mixture of solvent that resulted in the highest content of phenolic compounds; f) The sample is mixed with a 75 µg/mL solution of ACBD (abnormal CBD) in MeOH to a final concentration of 25 mg/mL; g) The entry assigned in this column corresponds to the analytical method entry presented in Table 1, Table 2, Table S2, and Table S3. *Abbreviations:* ACN, acetonitrile; BUT, butane; DCM, dichloromethane; DSLME, dispersive solid-liquid microextraction; DM, dynamic maceration; DME, dimethyl ether; EtOAc, ethyl acetate; EtOH, ethanol; ES, eutectic solvent; FA, formic acid; *n*-Hex, *n*-hexane;

HP-β-CD, 2-hydroxypropyl-β-cyclodextrin; IPA, isopropanol; IS, internal standard; LLE, liquid-liquid extraction; MeOH, methanol; MTBE, Methyl *tert*-butyl ether; NP, nanoparticles; NR, not reported; NTP, normal temperature pressure; PBS, phosphate buffer saline; RT, room temperature; $scCO_2$, supercritical carbon dioxide; SFE, supercritical fluid extraction; SLE, Solid-liquid extraction; SPE, solid phase extraction; SRS, secondary reference standards; UAE, ultrasound-assisted extraction; US-SLE, ultrasound-assisted solid-liquid extraction; US-LLE, ultrasound-assisted liquid-liquid extraction.

Cannabinoids Abbreviations: ACBD, abnormal cannabidiol; CBD, cannabidiol; CBDA, cannabidiolic acid; CBDA-ME, cannabidiolic acid methyl ester; CBDP, cannabidiphorol; CBE, cannabielsoin; CBEA, cannabielsoic acid; CBDV, cannabidivarin; CBC, cannabichromene; CBCA, cannabichromenic acid; CBCO, cannabichromeorcin; CBCV, cannabichromevarin; CBCVA, cannabichromevarinic acid; CBG, cannabigerol; CBGA, cannabigerolic acid; CBGAQ, cannabigerol quinone acid; CBGV, cannabigerovarin; CBGVA cannabigerovarinic acid; CBL, cannabicyclol; CBLA, cannabicyclolic acid; CBN, cannabinol; CBNR, cannabinerol; CBT, cannabicitran; DCBD, dihydrocannabinol; 7-OH-CBD, 7-OH-cannabidiol; Δ^8-THC, Δ^8-tetrahydrocannabinol; Δ^9-THC, Δ^9-tetrahydrocannabinol; THCA, Δ^9-tetrahydrocannabinolic acid; THCB, Δ^9-tetrahydrocannabutol; THCH, Δ^9-tetrahydrocannabihexol; THCP, Δ^9-tetrahydrocannabiphorol; THCV, Δ^9-tetrahydrocannabivarin; THCVA, Δ^9-tetrahydrocannabivarinic acid.

Table S2. Summary of LC parameters used in gradient chromatographic runs for detection of 1 or 2 cannabinoids

Entry	Method [a]	Mobile Phase [b]			Column	Flow Rate (mL/min)	Run Time (min) [c]	Injection Vol. (μL) [d]	Temp. (ºC)	UV Detection (nm) [e]	Cannabinoids Analyzed [f]	Ref.
		Aqueous Phase (A)	Organic Phase (B)	Gradient % B								
SH01	HPLC – DAD		ACN	73.5 – 90	Zorbax Eclipse Plus C18 (100 x 4.6 mm, 3.5 μm)	0.5	32	10	30	220	2: CBD, Δ9-THC	[125]
SH02	UHPLC – DAD	0.05% FA	ACN	NR	Acquity UPLC HSS T3 (100 x 2.1 mm, 1.8 μm)	0.45	NR	10	40	NR	2: CBD, Δ9-THC	[149]
SH03	HPLC – DAD	0.1% FA	ACN	5 – 50	Phenomenex Luna C18 (2) (150 × 4.6 mm, 5 μm)	1	41 [d]	20	30	254	1-CBD	[81]
SH04	UHPLC – DAD	0.1% FA	ACN - 0.1%FA	15 – 86	Ascentis Express C18 (150 × 2.1 mm, 2.7 μm)	0.25	67	5	30	270	2: CBD, CBDA	[69]
SH05	UHPLC – DAD	0.1% FA	ACN - 0.1%FA	30 – 86	Ascentis Express C18 (150 × 2.1 mm, 2.7 μm)	0.25	56	3 or 5 [d]	30	270	2: CBD, CBDA	[69]
SH06	HPLC – DAD	0.1% FA	ACN - 0.1%FA	60 – 90	Ascentis C18 (150 × 2 mm) with a guard column	0.1	45	NR	32	210, 220, 235, 275	2: CBD, CBDA	[70]
SH07	HPLC – DAD	0.4% FA	ACN-0.4% FA	50 – 95	Zorbax RP C18 (150 x 4.6 mm, 5 μm)	1	10	20	25	210	2: CBD, CBG	[150]
SH08	HPLC – DAD	0.1% AcOH	ACN – 0.1% AcOH	5 – 95	Waters Sunfire C18 (250 × 4.6 mm, 5 μm)	0.8	60 [d]	20	25	220	2: CBDA, CBDVA	[78]
SH09	HPLC – DAD	2% AcOH	ACN	3 – 95	Zorbax Eclipse Plus C18 (250 x 4.6, 5 μm)	1	90	10	NR	280, 320, 360	1: Δ9-THC	[103,151]
SH10	HPLC – DAD	0.1%TFA – 2%ACN	ACN - 0.1%TFA – 2%H_2O	43 – 56	Phenomenex Kinetex C18 (100 × 4.6 mm, 1.7 μm)	2.0	50 [d]	NR	40	220, 280	2: CBD, CBDA	[18]
SH11	UHPLC – DAD	50 nmM AF 10% MeOH	MeOH – 10% H_20 – 50mM AF	75 – 100	Kinetex Polar C18 (100 × 3 mm, 2.6 μm)	0.5	24	3	NR	210	1: CBD	[109]
SH12	HPLC – UV	20 mM $Na_3(PO4)_2$ (pH 3.0)	MeOH	70 – 90	Syncronis aQ C18 (150 × 4.6 mm, 5 μm)	1	18.5	20	NR	220	2: CBD, CBG	[130]

AcOH: acetic acid; ACN: acetonitrile; AF: ammonium formate; DAD: Diode Array Detector, FA: formic acid, HPLC: high-performance liquid chromatography, MeOH: methanol; NR: not reported; TFA: trifluoroacetic acid; UHPLC: ultra high-performance liquid chromatography; UV: ultraviolet.
Cannabinoids Abbreviations: CBD: cannabidiol; CBDA: cannabidiolic acid; CBDVA: cannabidivarinic acid; CBG: cannabigerol; Δ9-THC: Δ9-tetrahydrocannabinol.
[a] Type of liquid chromatography apparatus and UV detector used; when not specified, the UV term is used. Entries S04 and S05: Qualitative analysis by UHPLC-PDA-ESI-MS/MS. [b] Entry S01: Only water was used as aqueous phase. [c] When reported, run time includes column equilibration; Entries S3, S08, and S10: No column equilibration was reported in the description of the gradient. [d] Entry S05: CBD oil: 5 μL; Inflorescence: 3 μL. [e] Wavelengths used for cannabinoid detection and/ or quantification. [f] Entry S12: Both CBG and CBD have the same retention time.

Table S3: Summary of LC parameters used in isocratic chromatographic runs for detection of 1 or 2 cannabinoids

Entry	Method [a]	Mobile Phase [b]			Column	Flow Rate (mL/min)	Run Time (min)	Injection Vol. (µL) [c]	Temp. (ºC)	UV Detection (nm) [d]	Cannabinoids Analyzed	Ref.
		Aqueous Phase (A)	Organic Phase (B)	Gradient % B								
SH13	HPLC – DAD		ACN	20:80	RP-8 (250 x 4mm, 5 µm) with a guard column	1	8	10	30	220, 240	2: CBD, Δ⁹-THC	[152,153]
SH14	HPLC – DAD		ACN	25:75	Restek, C18 (150 × 4.6 mm, 5 µm)	1	10	NR	NR	205	1: CBD	[139]
SH15	HPLC – UV/Vis		ACN	25:75	SOLAS C18 (150 × 4.6 mm, 5 µm)	1.5	20	10	25	214	2: CBD, Δ⁹-THC	[154]
SH16	HPLC – DAD		ACN	25:75	C18 (250 × 4.6 mm, 5 µm)	1	30	10	NR	208	2: CBD, Δ⁹-THC	[126]
SH17	HPLC – UV		ACN	30:70	YMC C18 (250 × 4.6 mm, 5 µm)	1	NR	NR	30	220	1: CBD	[146]
SH18	HPLC – DAD		ACN	38:62	ACE C18-PFP (150 × 4.6 mm, 3 µm) with a guard column	1	30	40	55	220	1: CBD	[147]
SH19	HPLC – DAD		ACN	55:45	Princeton Sphere Ultima C-18 (250 × 4.6 mm, 5 µm)	1	15	20	30	210	1: CBD	[136]
SH20	HPLC – DAD	0.1% FA	ACN – 0.1% FA	20:80	Purospher STAR RP-18 (120 × 4.6 mm, 5 µm)	1	10	NR	25	208: CBD<br>280: Δ⁹-THC	2: CBD, Δ⁹-THC	[16]
SH21	HPLC – DAD	0.1% FA	ACN – 0.1% FA	20:80	Zorbax SB-C18 (250 × 4.6 mm, 5 µm)	1	20	10	35	220	1- CBD	[155]
SH22	HPLC – DAD	0.1% FA	ACN – 0.1% FA	40:60	Cortecs shield RP18 (150 x 4.6, 2.7 µm) with a guard column	1.6	25	10 or 50 [c]	30	223	1: THCA	[59]
SH23	HPLC – UV	AF 5 mM – 0.1% FA	ACN – 0.1% FA	25:75	Raptor ARC-18 (150 × 4.6 mm, 2.7 µm)	1.5	10	5	30	228	2: CBD, CBDA	[122]
SH24	HPLC – UV	0.1% TFA	ACN	25:75	Grace Davison C18 (250 × 4.6 mm; 5 µm)	0.8	20	20	NR	210	1: CBD	[137]
SH25	HPLC – DAD	0.1% AcOH	ACN – 0.1% AcOH	15:85	Kinetex C18 (150 x 2.1 mm × 2.9 µm) with a guard column	0.25	5	1-4	30	NR	1: CBD	[138]
SH26	HPLC – DAD	0.1% AcOH	ACN – 0.1% AcOH	40:60	Waters Sunfire C18 (250 × 4.6 mm, 5 µm)	0.8	40	20	25	220	2: CBDA, CBDVA	[78]
SH27	HPLC – DAD	0.2% AcOH	ACN	15:85	Infinity Lab Poroshell 120 EC-C18 (250 x 4.6 mm, 2.7 µm) with a guard column	0.6	NR	5	25	280	1: CBD	[135]
SH28	HPLC – UV	0.5% AcOH	ACN	30:70	ACE C18-AR	2	20	10	25	220	1: Δ⁹-THC	[156]
SH29	HPLC – UV/Vis	Phosphate buffer (pH 2.5)	ACN	30:70	ACE RP C18 (250 × 4.6 mm; 5 µm)	2	13.5	20	40	220	1: Δ⁹-THC	[120]

Entry	Method [a]	Mobile Phase [b]			Column	Flow Rate (mL/min)	Run Time (min)	Injection Vol. (µL)[c]	Temp. (ºC)	UV Detection (nm) [d]	Cannabinoids Analyzed	Ref.
		Aqueous Phase (A)	Organic Phase (B)	Gradient % B								
SH30	HPLC – UV		MeOH	15:85	Dikma Platisil ODS (250 × 4.6 mm, 5 µm)	1.2	10	20	NR	220	1-CBD	[141]
SH31	HPLC – DAD		MeOH	17.5:82.5	Shimadzu shim-pack GIST C-18 (150 x 4.6 mm, 5 µm)	1	15	10	30	210, 228	1: CBD	[134]
SH32	HPLC – DAD		MeOH	20:80	RP-C18 with a guard column	1	55	20	30	220	2: CBD, Δ9-THC	[44,47]
SH33	HPLC – UV	Phosphate buffer (0.1% PA, pH 2.16)	MeOH	10:90	Phenomenex C18 (250 × 4.6 mm, 5 µm) with a guard column	0.9	NR	40	30	220	1: CBD	[145]
SH34	HPLC – UV/Vis		MeOH:THF (93.4:6.6)	24:76	Zobrax SB-C18 (150 x 4.60 mm, 5 µm)	1	NR	NR	NR	228	1: CBD	[131,132]

AcOH: acetic acid; ACN: acetonitrile; AF: ammonium formate; DAD: Diode Array Detector, FA: formic acid, HPLC: high-performance liquid chromatography, MeOH: methanol; NR: not reported; TFA: trifluoroacetic acid; THF: Tetrahydrofuran; UHPLC: ultra high-performance liquid chromatography; UV: ultraviolet; Vis: visible.
Cannabinoids Abbreviations: CBD: cannabidiol; CBDA, cannabidiolic acid: CBDVA: cannabidivarinic acid; CBG: cannabigerol; Δ^9-THC: Δ^9-tetrahydrocannabinol; THCA: Δ^9-tetrahydrocannabinolic acid.
[a] Type of liquid chromatography apparatus and UV detector used; when not specified, the UV term is used. [b] Entry S13–19, S30–32, and S34: Only water was used as aqueous phase. [c] Entry S22: standards: 10 µL; Samples: 50 µL. [d] Wavelengths used for cannabinoid detection and/ or quantification.

Table S4. Summary of validation parameters: Linearity, Precision, Accuracy, LOD and LOQ.

Entry	CB	R^2	L. range (µg/mL)	Accuracy % [a]		LOD [b] (µg/mL)	LOQ [b] (µg/mL)	Precision (RSD%) [c]					Guidelines	HPLC entry [d]	Ref
SV01				*Spiking placebo solutions*				*Repeatability (6 samples, n = 2)*	*Intermediate (IP) (6 samples, n = 2)*	*Inter-Analyst (IA)*					
	CBD	0.9999	5 – 15	100 – 105		NR	NR	0.23	0.14	0.26			ICH Q2 (R1)	S15	[133]
	Δ^9-THC	0.9999	0.5 – 1.5	98 – 100		NR	NR	0.43	0.64	0.94					
SV02				*Spiking surrogate matrices (SM) (1-3 conc)*		*Based on SD of a linear response and a slope*		*Instr. P*	*Method P*		*Method IP (IA)*				
				For Inflorescences (chamomile)	*For Oil (olive oil)*	*Inflorescence (µg/g)*		*Solv*	*Infl*	*Oil*	*Infl SM*	*Oil SM*			
	CBC	0.99999	3.1 – 250	100.8	NR	20	60	0.32	1.41	NR	1.23	NR	ICH Q2 (R1)	65	[67]
	CBD	0.99998	3.0 – 240	95.3 – 102.4	98.2 – 103.1	35	107	1.49	1.15	1.27	0.67	1.60			
	CBDA	0.99998	2.8 – 230	102.4	101.9	41	123	0.10	1.24	NR	3.01	NR			
	CBDV	0.99998	3.1 – 240	103.1	NR	39	118	0.44	NR	NR	NR	NR			
	CBG	0.99996	2.9 – 230	95.4	NR	57	172	2.00	1.61	NR	1.76	NR			
	CBGA	0.99998	3.0 – 240	97.9 – 109.3	NR	31	94	0.68	1.76	NR	1.70	NR			
	CBN	0.99999	2.8 – 220	102.9	NR	55	167	0.38	3.58	NR	4.58	NR			
	Δ^8-THC	0.99998	3.1 – 250	90.1	NR	48	144	1.14	NR	NR	NR	NR			
	Δ^9-THC	0.99993	3.0 – 240	100 – 105.1	95.4 – 98.2	78	238	1.21	1.23	1.32	2.34	1.28			
	THCA	1.00000	3.0 – 240	96.9	100.7	32	96	0.68	2.09	NR	3.97	NR			
SV03				*Spiking placebo matrix – cannabis extraction waste (CEW) (3 conc)*		*Based on S/N*		*Method P Samples (n = 3)*	*Spiking CEW (3 conc.)*						
				Intra-day Day1	*Inter-day*	*S/N ≥ 3*	*S/N ≥ 10*		*Intra-day (n = 6)*	*Inter-day (3 days)*					
	CBC	0.9997	5 – 75	90 – 94	90.57 – 94.22	1.1	3.3	0.58 – 10.63	0.53 – 7.86	2.12			ICH Q2 (R1)	81	[60]
	CBCA	0.9996	5 – 75	81 – 93	83.15 – 92.33	1.6	4.8	5.00 – 13.63	2.21 – 9.84	5.26					
	CBD	0.9998	5 – 75	95 – 102	95.94 – 101.04	0.3	0.9	1.48 – 8.34	0.58 – 1.74	2.72					
	CBDA	0.9997	5 – 75	87 – 100	91.42 – 100.67	0.3	0.9	1.13 – 6.70	0.73 – 2.06	4.81					
	CBDV	0.9997	5 – 75	95 – 101	95.60 – 99.17	1.3	3.9	4.61 – 5.46	0.35 – 1.41	1.96					
	CBDVA	0.9999	5 – 75	86 – 97	90.10 – 97.67	1.3	3.9	4.13 – 8.45	0.44 – 1.36	4.05					
	CBG	0.9995	5 – 75	95 – 100	95.53 – 98.03	0.4	1.2	2.63 – 14.58	1.37 – 2.21	1.32					
	CBGA	0.9996	5 – 75	89 – 101	90.84 – 99.22	1.6	4.8	1.13 – 11.92	1.53 – 2.84	4.44					
	CBL	0.9998	5 – 75	93 – 98	94.28 – 96.28	0.7	2.1	NR	0.71 – 2.55	1.11					
	CBN	0.9998	5 – 75	93 – 97	93.24 – 97.37	0.5	1.5	1.43 – 14.46	0.43 – 1.25	2.31					
	Δ^8-THC	0.9994	5 – 75	93 – 96	91.11 – 95.02	1.1	3.3	0.82 – 13.78	1.52 – 4.15	2.27					
	Δ^9-THC	0.9997	5 – 75	94 – 99	94.81 – 96.89	0.8	2.4	1.34 – 8.61	0.92 – 2.32	1.16					
	THCA	0.9995	5 – 75	85 – 93	88.83 – 91.96	1.5	4.5	0.75 – 9.17	0.84 – 7.71	1.80					
	THCV	0.9998	5 – 75	95 – 101	95.68 – 98.56	0.2	0.6	1.86 – 8.97	0.65 – 1.60	1.63					
	THCVA	0.9909	5 – 75	84 – 95	88.66 – 94.41	1.5	4.5	NR	0.53 – 7.86	3.18					

Entry	CB	R^2	L. range (µg/mL)	Accuracy % [a]		LOD [b] (µg/mL)	LOQ [b] (µg/mL)	Precision (RSD%) [c]		Guidelines	HPLC entry [d]	Ref
**SV04**				*(1 conc; MeOH solutions)*		*Based on S/N* *S/N ≥ 3*	*S/N ≥ 10*	*Intra-day (1 Std solution, n= 3)*	*Inter-day (1 Std solution, 3 days)*			
	CBC	0.998	2.5 – 50	91.9		1.6	5.2	0.1 – 5.8	6.0	ICH Q2 (R2)	29	[157]
	CBD	0.999	51.5 – 206	104.0 (synthetic) 106.2 (natural)		0.4	1.3	4.8 – 6.5	6.1			
	CBDB	0.998	2.5–50	94.0		0.6	2.1	5.2 – 6.3	6.0			
	CBDV	0.998	2.5–50	93.9		0.3	1.0	4.5 – 9.3	7.4			
	Δ^9-THC	0.999	2.5–50	82.9		0.3	0.8	5 – 5.4	5.6			
	Δ^8-THC	0.996	2.5–50	96.6		1.0	3.4	1.5 – 7	6.2			
**SV05**				*Standard addition method (3 conc)*		*Based on SD of a linear response and a slope*		*Intra-day (3 conc, n= 3)*	*Inter-day (3 conc, 3 days)*			
	CBD	0.9951	10 – 60	97.84 – 99.34		0.483622	1.465521	0.24 – 0.54	0.19 – 0.47	ICH	S19	[136]
**SV06**				*Standard addition method* *Inflorescences*	*Resins*	*Based on standard deviation of a linear response and a slope*		*Intra-day (3 Std solutions; n = 3)*	*Inter-day (3 Std solutions; 3 days)*			
	CBC	0.9997	5 – 100	98	100	1.04	3.15	2.44	1.71	ICH Q2 (R2)	40	[45]
	CBD	0.9999	5 – 150	98	99	1.76	5.33	3.14	2.62			
	CBDA	0.9999	5 – 100	115	97	1.96	5.96	2.34	1.16			
	CBG	0.9998	5 – 100	97	80	1.00	3.03	2.68	1.61			
	CBN	0.9994	5 – 150	98	99	3.66	11.09	3.32	2.53			
	Δ^9-THC	0.9998	5 – 100	94	96	0.91	2.78	4.82	3.15			
	THCA	0.9998	5 – 100	99	103	1.82	5.59	4.27	1.84			
**SV07**				*Standard addition method (before extraction; 3 conc)* *Hemp pool*	*Seized pool*	*Based on SD of a linear response and a slope*		*Intra-day (3 Std solutions; n = 6)*	*Inter-day (3 Std solutions; 3 days)*			
	CBC	0.9999	0.5 – 50	83 – 87.7	86.9 – 89.5	0.03	0.09	0.05 – 0.25	0.36 – 1.16	ICH Q2 (R1)	15	[39]
	CBCT	0.9996	0.5 – 50	NR	NR	0.05	0.17	0.26 – 0.85	0.96 – 1.74			
	CBD	0.991	0.5 – 100	81.3 – 84.8	95.0 – 103.9	0.03	0.09	0.06 – 0.16	1.34 – 1.84			
	CBDA	0.9992	0.5 – 100	105.0 – 114.8	104.0 – 114.1	0.04	0.13	0.10 – 0.17	0.18 – 0.56			
	CBDV	0.9995	0.5 – 50	96.5 – 98.8	105.9 – 118.4	0.04	0.12	0.19 – 0.39	1.06 – 1.85			
	CBDVA	0.9997	0.5 – 50	102.2 – 104.3	93.4 – 94.6	0.04	0.14	0.07 – 0.99	0.21 – 2.04			
	CBE	0.9996	0.5 – 50	104.9 – 106.8	96.5 – 101.9	0.05	0.16	0.24 – 1.31	1.17 – 1.62			
	CBN	0.9998	0.5 – 50	112.5 – 116.3	107.8 – 110.2	0.02	0.06	0.11 – 0.31	0.57 – 1.30			
	CBGA	0.9999	0.5 – 50	100.8 – 108.2	96.6 –96.8	0.03	0.10	0.09 – 0.18	0.20 – 1.39			
	Δ^9-THC	0.996	0.5 – 100	105.9 – 116.2	108.3 – 117.3	0.04	0.14	0.09 – 0.42	1.12 – 1.86			
	Δ^9-*cis*-THC	0.9995	0.5 – 50	97.4 – 103.0	96.9 – 106.0	0.06	0.17	0.16 – 0.64	1.07 – 1.74			
	THCA	0.9997	0.5 – 100	100.7 – 103.1	110.2 – 111.3	0.04	0.11	0.07 – 0.21	0.22 – 0.29			
**SV08**			*% in oil*	*Spiking blank matrix (MCT oil) (before dilution, 3 conc),* % Bias		*% in oil*		*Intra-day (Std solutions; n = 3)*	*Inter-day (Std solutions; 3 days)*			
	Δ^9-THC	0.9957	0.03125 – 0.5	-5.0016 – 1.8606		0.01	0.03125	8.35 – 13.68	3.80 – 12.50	ICH Q2 (R1)	S28	[156]

Entry	CB	R^2	L. range (µg/mL)	Accuracy % [a]		LOD [b] (µg/mL)	LOQ [b] (µg/mL)	Precision (RSD%) [c]		Guidelines	HPLC entry [d]	Ref
SV09				*Intra-day (4 QC in human plasma; n = 5, % Bias)*	*Inter-day (4 QC in human plasma; % Bias)*			*Intraday (4 QC in human plasma; n = 5)*	*Interday (4 QC in human plasma)*			
	CBC	0.9958	0.1 – 4	-13.72 – -0.77	-2.28 – 0.29	NR	0.1	5.66 – 12.81	5.72 – 11.13	ICH M10	3	[144]
	CBD	0.9963	0.05 – 4	-18.30 – -2.61	-11.61 – 1.03	NR	0.05	2.16 – 10.12	6.51 – 15.93			
	CBG	0.9873	0.15 – 4	-14.11 – 7.62	-4.17 – 7.55	NR	0.15	2.52 – 8.21	5.58 – 13.25			
	CBN	0.9951	0.05 – 4	-11.57 – -3.49	-2.10 – 4.69	NR	0.05	1.69 – 11.09	8.84 – 11.75			
SV10				*Spiking experiment (enrichment oil samples, 3 conc)*		*Based on SD of a linear response and a slope S/N was also checked*		*Intra-day (Std solutions; n = 18)*	*Inter-day (Std solutions; 3 days)*			
	CBC	0.99940	0.1 – 85	99.51		0.10	0.50	5.12	4.64	ICH Q2 (R1)	80	[124]
	CBD	0.99951	0.1 – 85	99.43		0.05	0.50	5.41	4.96			
	CBDA	0.99937	0.1 – 85	99.75		0.05	0.50	3.76	3.31			
	CBDV	0.99947	0.1 – 85	98.32		0.05	0.50	4.67	4.13			
	CBG	0.99945	0.1 – 85	98.67		0.05	0.60	4.89	4.61			
	CBGA	0.99939	0.1 – 85	98.15		0.05	0.50	3.81	2.97			
	CBL	0.99959	0.1 – 85	97.24		0.10	0.61	5.30	4.74			
	CBN	0.99954	0.1 – 85	99.37		0.05	0.50	2.59	2.65			
	Δ^8-THC	0.99951	0.1 – 85	100.85		0.10	0.60	3.81	3.58			
	Δ^9-THC	0.99951	0.1 – 85	98.56		0.10	0.50	3.05	3.17			
	THCA	0.99940	0.1 – 85	101.47		0.13	0.59	5.42	5.04			
	THCV	0.99973	0.1 – 85	101.24		0.05	0.50	4.43	3.78			
SV11				*Spiking experiment (Extra virgin olive oil - EVOO, 3 conc)*		*Based on visual evaluation*		*Intra-day (Std in EVOO; 6 conc; n = 3)*	*Inter-day (Std in EVOO; 6 conc; 3 days)*			
	Δ^9-THC	0.9998	0.039 – 5.0	92.308 – 100		0.019	0.039	0.580 – 4.487	0.949 – 6.897	ICH Q2	S29	[120]
SV12				*Spiking experiment* *Intra-day (Std sol; 3 conc; n = 6)*	*Inter-day (Std sol; 3 conc; 3 days)*	*Based on SD of a linear response and a slope S/N was also checked*		*Intra-day (Std sol; 3 conc; n = 6)*	*Inter-day (Std sol; 3 conc; 3 days)*			
	CBD	0.9988	0.10 – 100	100.7 – 101.4	100.3 – 102.2	0.19	0.57	0.987 – 1.003	0.995 – 1.008	ICH Q2	S07	[150]
	CBG	0.9993	0.10 – 100	100.2 – 101.3	100.2 – 102.7	0.17	0.51	0.989 – 1.004	0.992 – 1.015			

Entry	CB	R^2	L. range (µg/mL)	*Intra-day/ Inter-day SM (1 conc)* Olive oil	*Intra-day/ Inter-day SM (1 conc)* Sunflower	*Standard addition* Samples	*Based on S/N* LOD $S/N \geq 3$	*Based on S/N* LOQ $S/N \geq 10$	*Intra-day (1 conc; n = 3)* Olive oil	*Intra-day (1 conc; n = 3)* Sunflower	*Inter-day (1 conc, 2 days)* Olive oil	*Inter-day (1 conc, 2 days)* Sunflower	Guidelines	HPLC entry	Ref
SV13	CBD	0.9973 – 0.9995	0.3 – 40	102.2/ 97.0	110.9/ 107.8	121	0.03	0.05	0.3	1.4	8.1	4.2	ICH Q2 (R2)	82	[158]
	CBN	0.9973 – 0.9995	0.3 – 40	100/ 94.6	108.7/ 105.7	105	0.03	0.05	0.3	0.4	7.4	4.8			
	Δ^9-THC	0.9976 – 0.9995	0.3 – 40	100/ 94.6	106.1/ 103.3	100	0.03	0.05	1.7	1.2	7.9	4.6			

Entry	CB	R^2	L. range (µg/mL)	Accuracy % [a]	LOD [b] (µg/mL)	LOQ [b] (µg/mL)	Precision (RSD%) [c]		Guidelines	HPLC entry [d]	Ref
SV14				*Standard addition method*	*Based on SD of a linear response and a slope*		*Intra-day (sample; n = 6)*	*Inter-day (sample; 6 days)*			
	CBC	0.9996	5 – 100	94.11	1.1	3.4	NR	NR	ICH Q2 (R1)	19	[97,159]
	CBD	0.9990	5 – 100	90.96	1.6	4.9	0.6	2			
	CBDA	0.9988	5 – 100	90.0	6.1	18.4	0.7	6.6			
	CBG	0.9955	5 – 100	84.2	2.5	7.7	NR	NR			
	CBGA	0.9997	5 – 100	84.7	1.3	3.9	0.9	2			
	CBN	0.9948	5 – 100	93.16	0.3	1.0	NR	NR			
	Δ^8-THC	0.999	5 – 100	67.7	1.4	4.2	NR	NR			
	Δ^9-THC	0.9977	5 – 100	89.67	2.3	6.9	0.7	3.2			
	THCA	0.9998	5 – 100	91.49	1.1	3.4	1.2	1.7			
	THCV	0.9999	5 – 100	91.75	0.7	2.1	NR	NR			
SV15				*(Std sol; 4 conc)*	*Based on S/N* *S/N ≥ 3*	*S/N ≥ 10*	*Intra-day (Std sol; 4 conc; n = 5)*	*Inter-day (Std sol; 4 conc; 3 days)*			
	CBC	0.996	1 – 100	7	0.85	2.54	0.4	0.2	AOAC Appendix K	93	[1,6–8,160]
	CBCA	0.996	5 – 100	5	3.15	9.50	0.8	0.2			
	CBD	0.999	1 – 500	10	0.80	2.68	0.6	0.08			
	CBDA	0.999	1 – 1000	3	0.36	1.21	0.7	0.1			
	CBDVA	0.999	1 – 50	3	0.21	0.70	1.7	0.8			
	CBG	0.999	1 – 100	2	0.77	2.57	0.5	0.2			
	CBGA	0.999	1 – 100	2	0.46	1.55	0.6	0.2			
	CBL	0.996	1 – 100	10	0.81	2.60	0.7	0.1			
	CBN	0.997	1 – 100	5	0.43	1.43	0.6	0.04			
	Δ^8-THC	0.999	1 – 100	10	1.05	3.12	0.8	0.2			
	Δ^9-THC	0.996	1 – 500	8	1.11	3.69	1.4	1.9			
	THCA	0.999	1 – 1000	4	0.76	2.52	0.5	0.4			
	THCV	0.999	1 – 100	11	0.63	2.51	0.7	0.1			
	THCVA	0.999	1 – 50	2	0.34	1.15	2.2	0.9			
SV16				*Spiking enriched cannabinoid mixtures to ointment base*	*Based on S/N* *S/N ≥ 3*	*S/N ≥ 10*	*Intra-day (Std sol; 3 conc; n = 3)*	*Inter-day (Std sol; 3 conc; 3 days)*			
	CBC	0.9998	2.5 – 50	104	0.10	1.0	0.5 – 3.4	3.0 – 4.5	AOAC Appendix K	92	[129]
	CBD	0.9999	2.5 – 50	101.1	0.25	2.5	0.6 – 3.9	1.4 – 3.7			
	CBDA	0.9998	2.5 – 50	100.7	0.10	1.0	0.5 – 2.6	1.1 – 3.7			
	CBG	0.9999	2.5 – 50	100.3	0.25	2.5	0.2 – 3.5	1.5 – 3.8			
	CBGA	0.9999	2.5 – 50	93.7	0.10	1.0	0.6 – 2.9	1.2 – 3.9			
	CBN	0.9999	2.5 – 50	101.3	0.10	1.0	0.4 – 4.1	1.1 – 3.5			
	Δ^8-THC	0.9999	2.5 – 50	NR	0.25	2..5	1.1 – 2.9	1.7 – 3.3			
	Δ^9-THC	0.9999	2.5 – 50	103.5	0.25	2.5	0.6 – 4.2	0.8 – 3.7			
	THCA	0.9998	2.5 – 50	94.4	0.25	2.5	0.2 – 2.1	1.6 – 4.6			
	THCV	0.9999	2.5 – 50	103.1	0.25	2..5	0.5 – 2.9	0.9 – 4.0			

Entry	CB	R^2	L. range (µg/mL)	Accuracy % [a]		LOD [b] (µg/mL)	LOQ [b] (µg/mL)	Precision (RSD%) [c]		Guidelines	HPLC entry [d]	Ref
**SV17**				*Spiking experiment (Blank matrix: Fibre-type herbal cannabis material, 3 conc, 3 – 4 days)*		*Based on S/N*		*Repeatability (blank matrix; 3 conc; n = 3)*	*IP (blank matrix; 3 conc; 3 – 4 days)*			
				Relative Bias %	*β-expectation tolerance interval %*	*S/N ≥ 3 (% w / w)*	*S/N ≥ 3 (% w / w)*					
	CBD	0.99819	6.2 – 143.9	-1.02 – 3.28	-11.70 – 12.48	0.02	0.05	0.76 – 2.45	2.72 – 4.61	ISO-17025	76	[28]
	Δ⁹-THC	0.99671	5.0 – 100.9	-0.57 – 2.21	-9.04 – 7.91	0.03	0.06	1.07 – 2.68	2.01 – 3.63			
	THCA	0.99761	4.96 – 99.2	0.12 – 2.93	-3.94 – 5.45	0.01	0.03	1.23 – 2.03	1.23 – 2.03			
	CBDA	0.9979	10 – 120	-0.24 – 0.63	-13.13 – 14.39	0.02	0.2	1.15 – 3.81	2.86 – 4.08	ISO-17025	76	[74]
**SV18**				*total overall bias %*	*β-expectation tolerance interval %*	*µg/g*		*Repeatability*	*IP*			
	CBD	1	2.5 – 50	-0.27 – 2.33	-6.54 – 5.99	1	5	0.387 – 0.62	0.387 – 1.29	ISO-17025	75	[143]
	CBDA	0.9998	2.5 – 50	-2.27 – -1.09	-9.95 – 7.77	1	5	0.45 – 2.95	0.651 – 3.391			
	Δ⁹-THC	1	2.5 – 50	-2.80 – -0.18	-9.83 – 4.95	1	5	0.5 – 2.903	1.115 – 2.903			
	THCA	0.9998	2.5 – 50	-5.25 – 1.02	-10.35 – 10.19	1	5	0.403 – 1.366	0.965 – 2.533			
**SV19**				*Intra-day – Day 1 (QC in solv; 3 conc)*	*Inter-day (QC in solv; 3 days)*			*Intra-day (QC in solv; 3 conc; n = 3)*	*Inter-day (QC in solv; 3 days)*			
	CBC	≥ 0.994	0.02 – 25	97.4 – 108.3	100 – 105.5	NR	0.02	0.1 – 2.5	1.7 – 2.7	ISO-17025	84	[73]
	CBCA		0.02 – 25	90.2 – 102.3	95.2 – 105.7	NR	0.02	0.1 – 5.4	2.8 – 5.4			
	CBD		0.02 – 25	97.5 – 100.2	100.5 – 103.7	NR	0.02	0.3 – 2.7	3.3 – 5.6			
	CBDA		0.02 – 25	98.5 – 103.3	99.9 – 102.7	NR	0.02	0.1 – 3.1	0.5 – 3.6			
	CBDV		0.02 – 25	97.6 – 104.9	98.5 – 105.2	NR	0.02	0.0 – 4.9	0.8 – 2.7			
	CBDVA		0.02 – 25	100.3 – 106.2	99.4 – 103.6	NR	0.02	0.1 – 8.6	2.2 – 5.4			
	CBG		0.02 – 25	96.2 – 102.7	99.6 – 100.6	NR	0.02	0.1 – 4.6	4.7 – 8.1			
	CBGA		0.02 – 25	98.1 – 105.4	100.1 – 103.8	NR	0.02	0.1 – 3.1	1.3 – 3.6			
	CBL		0.02 – 25	89.6 – 105.6	92.2 – 105	NR	0.02	0.2 – 4.2	1.1 – 6.0			
	CBLA		0.02 – 25	90.0 – 107.0	96.7 – 104.9	NR	0.02	0.1 – 5.3	1.7 – 8.7			
	CBN		0.02 – 25	97.7 – 104.5	99.8 – 104.5	NR	0.02	0.3 – 3.3	0.5 – 4.1			
	CBNA		0.02 – 25	97.1 – 109.4	99.6 – 106	NR	0.02	0.3 – 3.8	1.0 – 5.8			
	CBT		0.02 – 25	90.9 – 104.3	99.5 – 103.5	NR	0.02	0.7 – 8.0	1.2 – 8.2			
	Δ⁸–THC		0.02 – 25	95.9 – 99.0	100.5 – 103.4	NR	0.02	0.1 – 3.8	2.4 – 6.9			
	Δ⁹–THC		0.02 – 25	96.5 – 103.4	98.1 – 102.5	NR	0.02	0.2 – 5.3	0.8 – 4.7			
	THCA		0.02 – 25	91.1 – 112.3	99 – 107	NR	0.02	0.1 – 4.9	3.0 – 9.4			
	THCV		0.02 – 25	97.7 – 104.1	100.2 – 103.8	NR	0.02	0.4 – 3.4	0.4 – 4.1			
	THCVA		0.02 – 25	98.6 – 105.0	97.1 – 104.8	NR	0.02	0.1 – 7.0	0.2 – 3.7			
**SV20**				*Intra-day – Day 1 (QC in solv; 3 conc)*	*Inter-day (QC in solv; 3 days)*			*Intra-day (QC in solv; 3 conc; n = 3)*	*Inter-day (QC in solv; 3 days)*			
	Δ⁹-THC	0.9990-0.9992	0.04 – 50	101.3 – 104.0	100.8 – 106.8	NR	0.04	0.1 – 5.7	0.1 – 3.7	ISO-17025 + ANSI/ASB Standard 036	102	[34]
	THCA	0.9978-0.9987	0.04 – 50	100.8 – 105.0	99.3 – 104.5	NR	0.04	0.1 – 6.3	0.3 – 2.3			

Entry	CB	R^2	L. range (µg/mL)	Accuracy % [a]		LOD [b] (µg/mL)	LOQ [b] (µg/mL)	Precision (RSD%) [c]		Guidelines	HPLC entry [d]	Ref
				Intra-day – Day 1 (QC in solv; 3 conc)	*Inter-day (QC in solv; 3 days)*			*Intra-day (QC in solv; 3 conc, n = 3)*	*Inter-day (QC in solv; 3 days)*			
SV21	CBC	≥ 0.9938	0.02 – 25	95.5 – 100.4	94.8 – 100.7	NR	0.02	0.2 – 3.2	0.8 – 2.6	ISO-17025	86	[36]
	CBCA		0.02 – 25	96.6 – 102.8	96.1 – 107.4	NR	0.02	0.3 – 2.7	1.1 – 3.8			
	CBD		0.02 – 25	92.8 – 112.4	90.9 – 105.2	NR	0.02	0.1 – 5.6	0.6 – 5.9			
	CBDA		0.02 – 25	94.2 – 103.1	95.9 – 101.9	NR	0.02	0.1 – 4.5	1 – 4.7			
	CBDV		0.02 – 25	98.3 – 105.1	96.1 – 102.6	NR	0.02	0.2 – 3.7	1.3 – 3.3			
	CBDVA		0.02 – 25	94.6 – 99.7	94.7 – 100.5	NR	0.02	0.2 – 7.3	0.3 – 3.3			
	CBG		0.02 – 25	97.8 –103.3	96.3 – 101.5	NR	0.02	0.1 – 5.1	1.8 – 6.2			
	CBGA		0.02 – 25	91.6 –98.4	94.6 – 99.2	NR	0.02	0.2 – 7.9	0.6 – 7.3			
	CBN		0.02 – 25	65.2 – 104.4	94.9 – 102.4	NR	0.02	0.0 – 7.2	0.7 – 3.5			
	CBT		0.02 – 25	94.6 – 108.6	94.7 – 103.4	NR	0.02	0.2 – 4.2	0.4 – 9.7			
	Δ^8-THC		0.02 – 25	95.3 – 109.9	94.6 – 108.2	NR	0.02	0.3 – 5	0.9 – 4.7			
	Δ^9-THC		0.02 – 25	95.3 –110.7	94.7 – 105.8	NR	0.02	0.1 – 6.3	0.8 – 5.4			
	THCA		0.02 – 25	95.3 – 98.7	95.3 – 98.8	NR	0.02	0.1 – 5.6	0.3 – 3.6			
	THCV		0.02 – 25	95.1 – 103.5	94.8 – 101	NR	0.02	0.1 – 4.8	0.4 – 3.1			
	THCVA		0.02 – 25	95.1 – 102.6	96.8 – 106	NR	0.02	0.2 – 4.3	2.8 – 5.4			
				Intra-day – Day 1 (QC in solv; 3 conc)	*Inter-day (QC in solv; 3 days)*	*Based on S/N* *S/N ≥ 3*	*S/N ≥ 10*	*Intra-day (QC in solv; 3 conc, n = 3)*	*Inter-day (QC in solv; 3 days)*			
SV22	CBC	≥ 0.9899	0.04 – 50	98.2 – 102.6	98.3 – 101.9	0.009	0.029	0.1 – 7.2	0.9 – 2.3	ISO-17025	61	[35]
	CBCA		0.04 – 50	94.2 – 111.6	95.7 – 110.9	0.007	0.022	0.1 – 4.5	0.5 – 4.5			
	CBDV		0.04 – 50	98.2 – 103.1	97.3 – 103.6	0.005	0.016	0.4 – 3.2	0.3 – 1.1			
	CBDVA		0.04 – 50	98.4 – 102.8	96.7 – 103.4	0.004	0.012	0.2 – 5.3	0.1 – 3.8			
	CBD		0.04 – 50	97.5 – 101.5	97.1 – 100.7	0.007	0.023	0.0 – 9.6	0.7 – 1.4			
	CBDA		0.04 – 50	99.1 – 105.5	98.7 – 106.3	0.005	0.018	0.2 – 7.5	0.3 – 1.5			
	CBG		0.04 – 50	98.5 – 105.4	98.5 – 104.6	0.006	0.022	0.1 – 6.1	0.2 – 0.7			
	CBGA		0.04 – 50	97.3 – 104.2	98.2 – 105.1	0.007	0.022	0.3 – 4.9	1 – 3.3			
	CBL		0.04 – 50	99.3 – 104.0	99.8 – 107.4	0.010	0.034	0.7 – 3.7	0.4 – 3.1			
	CBLA		0.04 – 50	96.2 – 104.7	96.2 – 105	0.012	0.039	0.1 – 8.2	0.7 – 2			
	CBN		0.04 – 50	98.3 – 105.7	98.1 – 102.8	0.004	0.015	0.0 – 4.5	0.2 – 3.2			
	CBNA		0.04 – 50	98.4 – 104.0	98.6 – 108.6	0.003	0.011	0.1 – 7.2	0.1 – 1.2			
	Δ^8-THC		0.04 – 50	100.7 – 105.0	101.6 – 104.7	0.012	0.040	0.5 – 5.1	0.7 – 2.7			
	Δ^9-THC		0.04 – 50	93.9 – 101.8	95.1 – 102.3	0.011	0.038	0.3 – 9.5	0.4 – 1.5			
	THCV		0.04 – 50	84.8 – 103.3	89.5 – 103.7	0.007	0.024	0.1 – 5.8	0.7 – 5.3			
	THCA		0.04 – 50	99.2 – 105.7	98.1 – 106.2	0.009	0.029	0.0 – 5.9	0.9 – 2.7			
	THCVA		0.04 – 50	97.9 – 105.5	98.2 – 105.4	0.007	0.025	0.7 – 4.2	0.7 – 2.5			
	CBT		0.04 – 50	95.8 – 105.0	97 – 103.6	0.010	0.033	0.1 – 2.8	0.5 – 1.6			

Entry	CB	R^2	L. range (µg/mL)	Accuracy % [a]		LOD [b] (µg/mL)	LOQ [b] (µg/mL)	Precision (RSD%) [c]		Guidelines	HPLC entry [d]	Ref
**SV23**				*Intra-day – Day 1 (QC in solv; 3 conc)*	*Inter-day (QC in solv; 3 days)*			*Intra-day (QC in solv; 3 conc, n = 3)*	*Inter-day (QC in solv; 3 days)*			
	CBC	≥ 0.9880	0.02 – 25	94.7 – 107.5	98.2 – 107.4	NR	0.02	0.2 – 4.2	0.8 – 3.6	ISO-17025	70	[114]
	CBCA		0.02 – 25	97.5 – 114.7	94.8 – 114.1	NR	0.02	0.6 – 7.8	1.7 – 3.7			
	CBD		0.02 – 25	98.6 – 101.5	98.1 – 106.1	NR	0.02	0.5 – 4.4	1.0 – 3.8			
	CBDA		0.02 – 25	93.9 –107.6	92.4 – 108.2	NR	0.02	0.2 – 2.4	0.7 – 1.8			
	CBDV		0.02 – 25	84.5 – 107.6	93.8 – 106.5	NR	0.02	0.4 – 4.2	1.0 – 8.8			
	CBDVA		0.02 – 25	93.9 – 110.4	91.7 – 108.8	NR	0.02	0.4 – 4.8	0.8 – 4.7			
	CBG		0.02 – 25	98.6 – 101.5	99.0 – 104.0	NR	0.02	0.1 – 5.4	0.9 – 2.1			
	CBGA		0.02 – 25	96.2 – 106.3	96.1 – 108.2	NR	0.02	0.7 – 10.3	0.2 – 1.7			
	CBN		0.02 – 25	93.9 – 105.4	93.9 – 106.2	NR	0.02	0.2 – 1.7	0.9 – 1.8			
	Δ^8–THC		0.02 – 25	90.5 – 108.6	92.1 – 104.5	NR	0.02	0.4 – 8.9	3.4 – 5.8			
	Δ^9–THC		0.02 – 25	97.0 – 104.7	98.2 – 105.2	NR	0.02	0.6 – 4.1	0.7 – 2.6			
	THCA		0.02 – 25	96.4 –109.8	95.1 – 108.9	NR	0.02	0.2 – 9.6	0.9 – 5.6			
	THCV		0.02 – 25	98.6 – 114.5	98.5 – 105.4	NR	0.02	0.3 – 9.8	1.3 – 14			
	THCVA		0.02 – 25	96.4 – 102.6	97.1 – 105.3	NR	0.02	0.2 – 7.9	0.7 – 2.9			
**SV24**				*(1 conc, n = 10)*				*(1 conc, n = 10)*				
	CBC	NR	NR	96		0.118	0.393	2.631		ISO–17025	33	[115]
	CBCA	NR	NR	103		0.091	0.304	3.802				
	CBD	NR	NR	98		0.156	0.519	6.809				
	CBDA	NR	NR	110		0.109	0.364	2.118				
	CBDV	NR	NR	100		0.113	0.375	4.830				
	CBDVA	NR	NR	105		0.076	0.253	3.074				
	CBG	NR	NR	88		0.099	0.331	4.798				
	CBGA	NR	NR	110		0.211	0.705	8.179				
	CBL	NR	NR	109		0.237	0.790	2.311				
	CBLA	NR	NR	97		0.197	0.658	2.168				
	CBN	NR	NR	97		0.068	0.226	2.997				
	CBNA	NR	NR	104		0.084	0.280	3.454				
	Δ^9–THC	NR	NR	106		0.163	0.545	6.598				
	THCA	NR	NR	93		0.186	0.619	8.512				
	THCV	NR	NR	85		0.241	0.803	12.154				
	THCVA	NR	NR	101		0.110	0.366	4.632				
**SV25**				*Hemp-free blank products*		*Based on SD of a linear response and a slope and*		*Precision (RSD %)*	*Product*			
				% Bias	*% Recovery*	*S/N ≥ 3 (mg/g)*	*S/N ≥ 10 (mg/g)*					
	CBD	0.997	14 – 75	3	101	0.025	0.400	NR	Shikai Lotion	ANSI/ASB standard	88	[116]
	CBD	0.997	1 – 20	-4	101	0.062	0.207	NR	Hempz Lotion			
	CBD	0.997	20 – 100	2	95	0.121	0.403	NR	Zen Renu Cream and Neu Hemp Cream			
	Δ^9-THC	0.999	1 – 20	6	93	0.005	0.016	NR				
	CBD	0.996	1 – 50	0	97	0.340	1.134	NR	Pure Ratios Salve			

Entry	CB	R^2	L. range (µg/mL)	Accuracy % [a]		LOD [b] (µg/mL)	LOQ [b] (µg/mL)	Precision (RSD%) [c]				Guidelines	HPLC entry [d]	Ref
**SV26**				*Spiking experiment (coconut oil, 3 conc)*		*Based on S/N*<br>*S/N ≥ 2 or 3*	*S/N ≥ 10*	*3 conc, n = 6*						
	CBD	0.999	0.5 – 5	87.92 – 102.21		0.10	0.5	0.67 – 5.97				SANTE	S02	[149]
	Δ^9-THC	0.998	0.5 – 5	95.85 – 106.44		0.12	0.5	1.68 – 6.54						
**SV27**				*3 conc*	*Standard Addition Method*			*Intra-day (3 conc)*		*Inter-day (3 conc)*				
	CBD	1	0.78 – 50	100.04 – 101.7	91.2 – 102.8	0.28	0.84	0.05 – 0.39		0.78 – 1.42		FDA (ORA-LAB.5.4.5)	17	[104,117]
	CBDA	0.9996	0.78 – 50	98.66 – 101.23	ND	1.26	3.82	0.08 – 0.45		0.32 – 0.96				
	Δ^9-THC	0.9996	0.78 – 50	100.47 – 101.60	ND	1.28	3.89	0.05 – 0.27		0.71 – 1.25				
	THCA	0.9987	0.78 – 50	99.03 – 100.17	ND	0.23	0.71	0.10 – 0.38		0.26 – 0.80				
**SV28**				*Spiking experiment (3 conc)*<br>*Sunflower: coconut oil (60:40) - SM*	*SM oil in ice cream*	*Based on SD of a linear response and a slope LOQ recovery checked*		*Intra-day (1 conc, n = 6)*<br>*Oil*	*Ice Cream*	*Inter-day (1 conc, 3 days)*<br>*Oil*	*Ice Cream*			
	CBD	0.9977	1 – 5	98.8 – 101.1	86.8 – 92.2	0.11	0.33	1.5	1.7	3	3.5	ICH Q2 (R2) + AOAC Appendix F	S20	[16]
	Δ^9-THC	0.9918	20 – 100	98.8 – 102.5	84 – 94	0.76	2.30	1.7	1.6	3	3.2			
**SV29**				*Spiking experiment – blank matrix (3 conc)*		*Based on SD of a linear response and a slope*		*Intra-day (QC in solv; 3 conc, n = 3)*		*Inter-day (QC in solv; 3 days)*				
	CBD	0.9997	31.25 – 250	210 nm: 98.42 – 100.96<br>228 nm: 98.25 – 101.48		5.93<br>5.77	17.96<br>17.49	0.8 – 1.3<br>0.7 – 1.4		0.6 – 1.9<br>0.6 – 1.9		ICH Q2 (R1) + AOAC Appendix F	S31	[134]
**SV30**				*Standard addition method (5 conc)*		*Based on S/N*<br>*S/N ≥ 3*	*S/N ≥ 10*	*Repeatability (1 conc, n = 6)*		*IP (1 conc; 3 days)*		ICH Q2 (R1) + AOAC SMPR 2019.003 and Appendix K	S01	[125]
	CBD	0.9998	0.25 – 10.0	92 – 107		0.03	0.10	0.25 – 0.43		1.83				
	Δ^9-THC	0.9999	0.25 – 10.0	97 – 109		0.03	0.10	0.67 – 0.99		1.22				
**SV31**				*Standard addition (3 conc) %Bias*	*% Extraction recovery (3 conc)*	*Based on SD of a linear response and a slope (confirmed by S/N)*		*Repeatability (samples, n = 6)*		*Intermediate (samples, n= 6, 2 days)*		ICH Q2 (R1) + ANVISA RDC 166/2017 + USP + FDA	46	[30]
	CBD	0.9998	10 – 100	1.2 – 4.3	91.3 – 97.4	3.6	10.8	NR		NR				
	CBDA	0.9995	10 – 100	3.7 – 7.1	NR	3.3	10.1	NR		NR				
	CBN	0.9993	10 – 100	0.0 – 4.8	NR	4.1	12.5	NR		NR				
	Δ^9-THC	0.9995	10 – 100	0.9 – 2.2	NR	4.2	12.6	4 – 4.8		5.1				
	THCA	0.9995	10 – 100	0.4 – 1.7	NR	3.4	10.2	4.3 – 4.8		4.5				
**SV32**				*Spiking experiment – cannabis extraction waste (CEW)*		*LOD based on visual evaluation (QC from CRS)*		*Intra-day (QC from SRM in solv; 3 conc)*		*IP (QC from SRM in solv; 3 conc)*				
	CBD	1.0000	2 – 110	94.77 – 99.45		0.80	2	0.40 – 3.20		1.16 – 2.66		ICH Q2 (R1) + ANVISA RDC 166/2017	56	[48]
	CBDA	0.9997	2 – 110	88.32 – 94.71		0.60	2	0.38 – 1.69		2.16 – 3.33				
	CBN	1.0000	2 – 110	113.21 – 116.85		0.50	2	0.31 – 1.93		1.79 – 3.82				
	Δ^9-THC	1.0000	2 – 110	109.76 – 119.28		0.70	2	1.44 – 3.33		2.02 – 3.86				
	THCA	0.9999	2 – 110	76.92 – 82.07		0.40	2	0.32 – 3.18		1.47 – 4.27				

Entry	CB	R^2	L. range (μg/mL)	Accuracy % [a]			LOD [b] (μg/mL)	LOQ [b] (μg/mL)	Precision (RSD%) [c]		Guidelines	HPLC entry [d]	Ref
**SV33**				*Intra-day (3 conc, n = 7)*	*Inter-day (3 conc, 3 days)*		*Based on SD of a linear response and a slope LOQ by Visual evaluation*		*Repeatability (3 conc, n = 7)*	*IP (3 conc, 3 days)*	ICH + Eurachem + SANTE 11312/ 2021 + ANVISA RDC 166/2017 + FDA	48	[94]
	CBD	0.9856	10 – 120	101.3 – 113.7	99.3 – 119.3		0.19	5	3.6 – 4.8	2.3 – 4.6			
**SV34**				*% Bias* *Intra-day (QC in solv; 3 conc, n = 5)*	*Inter-day (QC in solv; 3 conc, 3 days)*		*Based on S/N and visual evaluation*		*Intra-day (QC in solv; 3 conc, n = 5)*	*Inter-day (QC in solv; 3 conc, 3 days)*			
	CBD	[0.9978 – 0.9988]	0.5 – 25.0	-5.30 – 14.49	-13.00 – 4.00		0.125	0.5	0.32 – 8.02	1.00 – 4.90	Eurachem + UNODC	74	[95]
	CBDA		0.5 – 25.0	-9.61 – 0.99	0.20 – 5.80		0.250	0.5	0.44 – 6.96	0.90 – 6.90			
	CBN		0.5 – 25.0	-4.81 – 13.02	-13.30 – 3.10		0.125	0.5	0.02 – 4.91	0.80 – 4.40			
	Δ⁸-THC		0.5 – 25.0	-1.95 – 3.39	-3.30 – 1.10		0.125	0.5	0.01 – 12.48	0.80 – 3.60			
	Δ⁹-THC		0.5 – 25.0	-1.24 – 7.68	-6.40 – 1.10		0.125	0.5	0.53 – 12.72	1.50 – 13.00			
	THCA		0.5 – 25.0	-6.84 – 14.50	-10.10 – 5.90		0.250	0.5	0.01 – 10.45	0.90 – 8.90			
**SV35**				*Standard addition method - Hemp essential oil (EO) (3 conc)*			*Based on S/N* *S/N ≥ 3*	*S/N ≥ 10*	*Intra-day (QC in EO, 3 conc, n = 3)*	*Inter-day (QC in EO, 3 conc, 3 days)*			
	CBD	0.9995	0.5 – 100	86 – 90			0.11	0.35	1.84 – 3.30	9.80	NR	20	[102]
	CBDA	0.9999	0.5 – 100	81 – 98			0.16	0.48	0.99 – 3.60	6.32 – 10.40			
	CBN	0.9997	0.5 – 100	90 – 97			0.13	0.40	0.83 – 3.00	3.75 – 4.80			
	Δ⁹-THC	0.9997	0.5 – 100	79 – 99			0.12	0.38	0.83 – 3.00	5.22 – 9.72			
	THCA	0.9993	0.5 – 100	78 – 100			0.15	0.47	0.92 – 2.10	7.75 – 9.55			
**SV36**				*Standard addition method (2 conc)*			*Based on SD of a linear response and a slope*		*Repeatability (2 conc; n = 6)*	*IP (2 conc; 3 days)*			
	CBD	0.9999	0.15 – 25	91.1 – 94.4			0.05	0.15	0.08 – 0.23	1.86 – 3.49	Eurachem	7	[41]
	CBDA	0.9999	0.25 – 25	93.8 – 97.5			0.08	0.25	0.04 – 0.05	0.07 – 0.31			
	CBN	0.9998	0.15 – 25	87.2 – 89.0			0.05	0.15	0.19 – 1.51	1.50 – 1.72			
	Δ⁹-THC	0.9998	0.2 – 25	94.0 – 95.0			0.07	0.20	0.36 – 0.84	2.37 – 1.59			
	THCA	0.9999	0.25 – 25	90.0 – 106.6			0.07	0.25	0.10 – 0.14	0.34 – 1.18			
**SV37**				*Spiking experiments – cannabis extraction waste (CEW)* *Intra-day (% Bias)*	*Inter-day (% Bias)*	*Extraction Recovery*	*Based on S/N* *S/N ≥ 3*	*S/N ≥ 10*	*Repeatability (QC in CEW, 6 conc; n = 6)*	*IP (QC in CEW, 3 conc; 6 days)*			
	CBC	0.999	3.125 – 100	-7.63 – 4.50	-1.26 – 9.77	79 – 98	0.284	0.948	1.11 – 4.91	0.89 – 4.22	ICH Q2 (R1), AOAC, FDA, USP	71	[93]
	CBD	0.999	3.125 – 100	-0.51 – 3.66	-30.80 – 9.62	83 – 113	0.450	1.502	0.78 – 6.63	0.43 – 4.91			
	CBDA	0.999	3.125 – 100	0.89 – 2.54	-74.67 – 4.44	77 – 102	0.367	1.226	0.74 – 2.89	0.45 – 2.82			
	CBDV	0.999	3.125 – 100	-2.07 – 7.63	6.86 – 9.85	84 – 114	0.335	1.116	0.63 – 1.29	0.34 – 1.00			
	CBG	0.999	3.125 – 100	-1.72 – 4.92	-6.16 – 8.22	81 – 121	0.367	1.224	0.86 – 4.00	0.87 – 2.82			
	CBGA	0.999	3.125 – 100	1.43 – 3.27	1.99 – 8.40	80 – 109	0.367	1.226	0.42 – 0.79	0.45 – 1.04			
	CBL	0.999	3.125 – 100	0.57 – 6.23	6.13 – 9.82	NR	0.351	1.171	1.05 – 9.39	0.61 – 8.19			
	CBN	0.999	3.125 – 100	0.42 – 4.41	8.21 – 9.57	NR	0.468	1.560	0.64 – 4.41	0.33 – 7.32			
	Δ⁸-THC	0.999	3.125 – 100	-1.37 – 6.67	5.54 – 10.49	77 – 111	0.200	0.669	0.38 – 8.73	0.44 – 7.28			
	Δ⁹-THC	0.999	3.125 – 100	-4.03 – 2.49	-182.83–4.66	74 – 89	0.229	0.766	1.31 – 5.53	1.02 – 6.81			
	THCA	0.999	3.125 – 100	-13.78 – 3.41	-6.16 – 8.34	76 – 93	0.342	1.140	3.10 – 3.75	1.88 – 4.04			
	THCV	0.999	3.125 – 100	-7.89 – 5.32	8.69 – 9.28	83 – 113	0.340	1.134	0.57 – 5.92	0.58 – 5.21			

Entry	CB	R^2	L. range (µg/mL)	Accuracy % [a]			LOD [b] (µg/mL)	LOQ [b] (µg/mL)	Precision (RSD%) [c]			Guidelines	HPLC entry [d]	Ref
				% Relative error<br>*Intra-day (QC in sample; 4-5 conc; n = 6)*	*Inter-day (QC in sample; 8-11 conc; 5 days, n = 5)*	*Interm. (QC in sample; 4-5 conc; 5 days, n = 15)*	*Based on visual evaluation µg/mL or µg/mg (semi-solid or solid)*		*Intra-day (QC in sample; 4-5 conc; n = 6)*	*Inter-day (QC in sample; 8-11 conc; 5 days, n = 5)*	*Intermediate (QC in sample; 4-5 conc; 5 days, n = 15)*			
**SV38**	*Beverage / oil*											ICH M10 + FDA + USP	98	[127]
	CBCA	0.9987	0.8 – 100	-5.12 – 3.24	-5.50 – 3.66	-7.65 – 6.40		0.8	3.44 – 11.28	2.25 – 11.10	3.23 – 13.30			
	CBD	0.9990	0.4 – 100	-5.51 – 2.36	4.60 – 2.77	-10.23 – -3.85		0.4	3.00 – 9.49	1.06 – 10.74	5.52 – 12.03			
	CBDA	0.9986	0.4 – 100	-4.28 – 7.48	-6.42 – 2.26	-9.60 – 2.76		0.4	3.27 – 9.32	2.32 – 10.36	3.04 – 12.49			
	CBN	0.9984	0.1 – 100	-12.36 – 10.17	-6.18 – 3.34	-8.02 – 7.24		0.1	3.34 – 10.27	1.65 – 12.58	3.62 – 11.49			
	CBG	0.9979	0.4 – 100	-5.96 – 3.76	-6.31 – 5.48	-6.29 – 0.28		0.4	3.48 – 6.73	1.90 – 10.81	5.65 – 11.94			
	CBGA	0.9987	0.4 – 100	-4.03 – 9.88	-6.03 – 3.29	-6.15 – 2.15		0.4	3.19 – 10.49	2.42 – 10.15	3.63 – 12.00			
	Δ^8-THC	0.9989	0.4 – 100	-8.96 – 2.87	-6.54 – 4.48	-10.78 – -3.58		0.4	3.73 – 9.14	2.21 – 9.76	4.88 – 10.74			
	Δ^9-THC	0.9988	0.4 – 100	-8.90 – 3.10	-5.02 – 2.52	-7.78 – -1.28		0.4	3.75 – 9.29	0.90 – 11.19	4.81 – 14.67			
	THCA	0.9967	0.4 – 250	-12.56 – 7.52	-9.27 – 6.00	-9.00 – 3.30		0.4	3.23 – 8.15	3.73 – 17.02	1.91 – 15.51			
	Herbal samples													
	CBCA	0.9953	0.08 – 10	-5.12 – 3.24	-5.50 – 3.66	-7.65 – 6.40		0.08	3.44 – 11.28	2.25 – 11.10	3.23 – 13.30			
	CBD	0.9953	0.04 – 10	-8.75 – -2.75	-6.00 – 4.25	-14.60 – 4.04		0.04	1.65 – 11.63	1.47 – 11.43	2.13 – 11.42			
	CBDA	0.9974	0.04 – 10	-14.5 – -0.15	8.75 – 2.00	13.2 – -0.04		0.04	3.62 – 11.39	1.45 – 10.61	1.81 – 12.42			
	CBN	0.9937	0.01 – 10	-12.36 – 10.17	-6.18 – 3.34	-8.02 – 7.24		0.01	3.34 – 10.27	1.65 – 12.58	3.62 – 11.49			
	CBG	0.9953	0.04 – 10	-5.96 – 3.76	-6.31 – 5.48	-6.29 – 0.28		0.04	3.48 – 6.76	1.90 – 10.81	5.65 – 11.94			
	CBGA	0.9945	0.04 – 10	-4.03 – 9.88	-6.03 – 3.29	-6.15 – 2.15		0.04	3.41 – 10.49	2.42 – 10.15	3.63 – 12.00			
	Δ^8-THC	0.9963	0.04 – 10	-9.96 – 2.87	-6.54 – 4.48	-10.78 – -3.58		0.04	3.73 – 9.14	2.21 – 9.76	4.88 – 10.74			
	Δ^9-THC	0.9975	0.04 – 10	-8.90 – 3.10	-5.02 – 2.52	4.81 – 14.67		0.04	3.75 – 9.29	0.90 – 11.19	4.81 – 14.67			
	THCA	0.9930	0.04 – 25	-12.56 – 7.52	-9.27 – 6.00	-9.00 – 3.03		0.04	3.23 – 8.15	3.73 – 17.02	1.91 – 15.51			
	Cosmetic products													
	CBCA	0.9940	0.08 – 10	-12.00 – 7.80	0.26 – 13.33	-12.92 – 4.90		0.08	2.34 – 5.87	1.68 – 12.12	1.12 – 3.40			
	CBD	0.9943	0.04 – 10	-2.70 – 5.42	-5.55 – 7.08	-4.63 – 6.67		0.04	1.90 – 4.57	1.80 – 8.43	1.50 – 8.87			
	CBDA	0.9959	0.04 – 10	-8.33 – 9.52	-6.25 – 9.18	-5.83 – 8.67		0.04	2.62 – 7.01	1.27 – 13.16	2.15 – 3.28			
	CBN	0.9928	0.01 – 10	-12.00 – 10.13	-11.00 – 6.34	-8.67 – 6.57		0.01	1.14 – 7.92	0.77 – 10.11	2.14 – 7.29			
	CBG	0.9941	0.04 – 10	5.40 – 10.97	-5.83 – 11.67	-5.00 – 5.42		0.04	3.06 – 7.79	1.24 – 10.73	1.49 – 4.16			
	CBGA	0.9935	0.04 – 10	-5.42 – 6.00	-7.10 – 12.92	-6.67 – 4.33		0.04	2.63 – 7.39	1.81 – 6.32	1.59 – 8.56			
	Δ^8-THC	0.9960	0.04 – 10	1.00 – 6.33	-11.04 – 12.08	-8.50 – 9.00		0.04	2.48 – 7.72	3.16 – 10.20	3.27 – 7.61			
	Δ^9-THC	0.9961	0.04 – 10	-7.50 – -0.70	-13.25 – 10.96	-3.88 – 5.33		0.04	0.63 – 4.16	3.16 – 10.20	4.70 – 7.94			
	THCA	0.9939	0.04 – 25	-9.50 – 5.12	-8.33 – 8.29	-1.83 – 10.33		0.04	4.59 – 8.49	1.18 – 14.13	1.47 – 4.76			
**SV39**									*Intra-day (3 conc)*	*Inter-day (3 conc)*		NR	97	[51]
	CBC	0.9997	1 – 1000	99.69			0.11025	0.36383	1.10 – 1.21	1.14 – 1.19				
	CBD	0.9999	1 – 1000	99.49			0.11512	0.37989	1.12 – 1.13	1.15 – 1.75				
	CBG	0.9999	1 – 1000	99.67			0.1112	0.36669	1.11 – 1.15	1.06 – 1.09				
	CBN	0.9998	1 – 1000	99.53			0.12545	0.41401	1.01 – 1.44	1.15 – 1.31				
	Δ^9-THC	0.9999	1 – 100	99.79			0.13212	0.43599	1.12 – 1.39	1.01 – 1.12				

Entry	CB	R^2	L. range (µg/mL)	Accuracy % [a]		LOD [b] (µg/mL)	LOQ [b] (µg/mL)	Precision (RSD%) [c]				Guidelines	HPLC entry [d]	Ref
**SV40**				*Standard addition method (2 conc.)* *Pre-extraction % Recovery*	*Post-extraction % Matrix effect*	*Based on SD of a linear response and a slope*		*(QC in solv; 3 conc; n = 7)*						
	CBDA	0.997	1 – 100	71.2 – 91.8	85.9 – 100.8	0.10	0.24	0.58 – 1.37				Peters et al. [161]	23	[29]
	THCA	0.998	1 – 100	68.6 – 101.0	99.8 – 102.4	0.12	0.37	0.82 – 1.47						
**SV41**				*Spiking experiments – Diluted extracts (3 conc)*		*Based on S/N and visual evaluation*		*Intra-day* *QC in solv; n = 10)*	*Intra-day* *QC in samples; n = 10)*	*Inter-day* *QC in solv; 10 days)*	*Inter-day* *QC in samples; 10 days)*			
	Δ^9-THC	0.9975	5 – 50	94.29 – 103.44		0.5	1	0.16	0.08 – 0.14	0.73	1.19–2.65	NR	67	[32]
**SV42**								*Intra-day*		*Inter-day (2 days)*				
	10 CB	>0.99	0.5 – 100	> 99		≤ 0.8	< 2	< 10				NR	99	[111]
**SV43**														
	CBD	NR	NR	95 – 112		NR	NR	≤ 5.5				NR	S23	[122]
	CBDA	NR	NR	99 – 102		NR	NR	≤ 4.6						
**SV44**						*Based on SD of a linear response and a slope*		*Intra-day (n = 6)*						
	CBCA	0.99952	0.16 – 10	NR		0.039	0.118	3.42				NR	94	[57]
	CBNA	0.99998	0.16 – 10	NR		0.010	0.030	1.87						
	THCA	0.99994	0.16 – 100	NR		0.043	0.130	3.15						
**SV45**				*Spiking experiments – honey (SM)*		*Based on S/N* *S/N ≥ 3*	*S/N ≥ 10*							
	CBD	0.999	1 – 100	≥ 95%		1	5	NR				NR	32	[88]
	CBDA	0.999	1 – 100	≥ 95%		1	5	NR						
	Δ9-THC	0.999	1 – 50	≥ 95%		1	5	NR						
	THCA	0.999	1 – 50	≥ 95%		1	5	NR						
**SV46**								*Intra-day (3 conc)*		*Inter-day (3 conc)*				
	CBC	0.9988	0.2 – 50	NR		0.1	NR	0.34 – 6.80		1.08 – 1.68		NR	91	[123]
	CBD	0.9977	0.1 – 50	NR		0.02	NR	0.21 – 3.51		0.86 – 1.07				
	CBDA	0.9978	0.1 – 50	NR		0.02	NR	0.81 – 4.97		0.87 – 1.05				
	CBDV	0.9972	0.1 – 50	NR		0.02	NR	0.26 – 1.74		1.71 – 0.82				
	CBG	0.9979	0.1 – 50	NR		0.02	NR	0.22 – 4.20		0.90 – 1.13				
	CBGA	0.9983	0.1 – 50	NR		0.02	NR	0.19 – 4.17		1.02 – 1.25				
	CBL	0.9985	0.2–50	NR		0.1	NR	0.33 – 5.61		1.05 – 1.55				
	CBN	0.9981	0.1 – 50	NR		0.02	NR	0.22 – 2.91		1.03 – 1.40				
	Δ^8-THC	0.9983	0.2 – 50	NR		0.1	NR	1.80 – 3.91		1.03 – 1.71				
	Δ^9-THC	0.9982	0.2 – 50	NR		0.1	NR	1.06 – 3.28		1.03 – 1.50				
	THCA	0.9992	0.2 – 50	NR		0.1	NR	0.24 – 4.72		1.11 – 1.95				
	THCV	0.9974	0.1 – 50	NR		0.02	NR	0.28 – 1.66		0.89 – 1.14				
	THCVA	0.9978	0.1 – 50	NR		0.02	NR	0.63 – 2.83		1.05 – 1.34				

Entry	CB	R^2	L. range (µg/mL)	Accuracy % [a]	LOD [b] (µg/mL)	LOQ [b] (µg/mL)	Precision (RSD%) [c]	Guidelines	HPLC entry [d]	Ref
**SV47**				*(QC in solvent; 2 batches; 3 conc; n = 3)*	*Based on S/N* *S/N ≥ 3*	*S/N ≥ 10*	*(QC in solvent; 2 batches; 3 conc; n = 3)*			
	Δ[8]-THC	1	NR	100.58 – 112	0.27	1.98	0.21 – 1.93	NR	41	[162]
	Δ[9]-THC	1	NR	100.38 – 112.90	0.25	1.55	0.25 – 1.76			
**SV48**							*Intra-day* / *Inter-day*			
	CBD	> 0.99	1.25 – 40	90 – 110	NR	NR	< 2 / < 2	NR	S30	[141]
**SV49**							*Inter-day (n = 10)*			
	CBC	0.9999	0.1 – 10	NR	0.03	0.08	1.3	NR	78	[148]
	CBD	0.9997	0.1 – 5	NR	0.02	0.08	7.7			
	CBG	0.9999	0.1 – 10	NR	0.02	0.07	5.6			
	CBN	1	0.1 – 10	NR	0.03	0.05	3.1			
	Δ[8]-THC	0.9994	0.1 – 5	NR	0.01	0.10	8.6			
	Δ[9]-THC	0.9997	0.1 – 5	NR	0.03	0.08	5.9			
**SV50**				*Spiking experiments – blank samples (normal gummy extract; 3 conc; n = 3) % Bias*	*Based on SD of a linear response and a slope Confirmed by visual evaluation*		*(3 conc; n = 3)*			
	Ag(I) HPLC-DAD							SWGTOX	104	[121]
	CBD	0.9988	5.00 – 333	-5.0 – 4.2	0.90	NR	0.62 – 5.6			
	Δ(4)8-iso-THC	NR	NR	-12 – 7.6	NR	NR	0.68 – 5.6			
	Δ[8]-iso-THC	NR	NR	3.2 – 7.2	NR	NR	0.26 – 6.1			
	Δ[8]-THC	0.9983	5.00 – 333	-3.6 – 5.6	0.50	NR	0.86 – 6.1			
	Δ[9]-THC	0.9988	5.00 – 333	-3.4 – 8.6	0.40	NR	2.2 – 5.5			

CB: Cannabinoids; CEW: Cannabis extraction waste (obtained after exhaustive extraction of cannabis material); conc: Concentration; CRS: certified reference standards EO: Essential oil; EVOO: Extra virgin olive oil; IA: Inter-analyst; Infl: Inflorescence; IP: Intermediate precision; L. range: Linear range; Instr. P: Instrument precision; Method P: Method precision; NR: not reported, not determined or not present in the corresponding sample: QC: Quality control; RSD: Relative standard deviation; SD: Standard deviation, SM: surrogate matrix; S/N: Signal-to-noise ratio; solv: Solvent; SRM: secondary reference material; Std: Standard.

Cannabinoids Abbreviations: CBD: cannabidiol: CBDA, cannabidiolic acid: CBDV, cannabidivarin: CBC: cannabichromene; CBCA: cannabichromenic acid; CBG: cannabigerol; CBGA: cannabigerolic acid; CBL: cannabicyclol; CBLA: cannabicyclolic acid; CBN: cannabinol; CBT: cannabicitran; Δ(4)8-iso-THC: Δ4(8)-iso-tetrahydrocannabinol; Δ[8]-iso-THC: Δ[8]-iso-tetrahydrocannabinol Δ[8]-THC: Δ[8]-tetrahydrocannabinol; Δ[9]-THC: Δ[9]-tetrahydrocannabinol; THCA: Δ[9]-tetrahydrocannabinolic acid; THCV: tetrahydrocannabivarin; THCVA: tetrahydrocannabivarinic acid.

[a] Accuracy: In this column, the main type of accuracy, trueness, bias, recovery described in the analyzed papers are presented. Accuracy intervals correspond to the smallest and the biggest values obtained for any concentration and replicate for the type of accuracy mentioned. When not specified, accuracy is reported as % of recovery. [b] LOD and LOQ values are expressed as mg/mL unless stated otherwise. [c] Precision intervals described in this table correspond to the smallest and the biggest RSD values obtained for any concentration and replicate for the type of precision mentioned. [d] The entry assigned in this column corresponds to the analytical method entry presented in Table 1, Table 2, Table S2, and Table S3.

References

1. Birenboim, M.; Brikenstein, N.; Duanis-Assaf, D.; Maurer, D.; Chalupowicz, D.; Kenigsbuch, D.; Shimshoni, J.A. In Pursuit of Optimal Quality: Cultivar-Specific Drying Approaches for Medicinal Cannabis. *Plants* **2024**, *13*, 1049, doi:10.3390/plants13071049.
2. Brikenstein, N.; Birenboim, M.; Kenigsbuch, D.; Shimshoni, J.A. Optimization of Trimming Techniques for Enhancing Cannabinoid and Terpene Content in Medical Cannabis Inflorescences. *Med. Cannabis Cannabinoids* **2024**, *7*, 111–118, doi:10.1159/000539192.
3. Birenboim, M.; Chalupowicz, D.; Kenigsbuch, D.; Shimshoni, J.A. Improved Long-Term Preservation of Cannabis Inflorescence by Utilizing Integrated Pre-Harvest Hexanoic Acid Treatment and Optimal Post-Harvest Storage Conditions. *Plants* **2024**, *13*, 992, doi:10.3390/plants13070992.
4. Gjorgievska, V.S.; Karanfilova, I.C.; Trajkovska, A.; Karapandzova, M.; Petrovska, B.B.; Kulevanova, S.; Stefkov, G. Monitoring of Cannabis Cultivar Technological Maturity by Trichome Morphology Analysis and HPLC Phytocannabinoid Content. *Pharmacogn. Res.* **2023**, *15*, 94–100, doi:10.5530/097484900306.
5. Gigopulu, O.; Geskovski, N.; Stefkov, G.; Stoilkovska Gjorgievska, V.; Slaveska Spirevska, I.; Huck, C.W.; Makreski, P. A Unique Approach for In-Situ Monitoring of the THCA Decarboxylation Reaction in Solid State. *Spectrochim. Acta - A: Mol. Biomol. Spectrosc.* **2022**, *267*, 120471, doi:10.1016/j.saa.2021.120471.
6. Birenboim, M.; Kenigsbuch, D.; Shimshoni, J.A. Novel Fluorescence Spectroscopy Method Coupled with N-PLS-R and PLS-DA Models for the Quantification of Cannabinoids and the Classification of Cannabis Cultivars. *Phytochem. Anal.* **2023**, *34*, 280–288, doi:10.1002/pca.3205.
7. Birenboim, M.; Chalupowicz, D.; Maurer, D.; Barel, S.; Chen, Y.; Fallik, E.; Paz-Kagan, T.; Rapaport, T.; Sadeh, A.; Kengisbuch, D.; et al. Multivariate Classification of Cannabis Chemovars Based on Their Terpene and Cannabinoid Profiles. *Phytochemistry* **2022**, *200*, 113215, doi:10.1016/j.phytochem.2022.113215.
8. Birenboim, M.; Rinnan, Å.; Kengisbuch, D.; Shimshoni, J.A. Novel Fluorescence Spectroscopy Coupled with PARAFAC Modeling for Major Cannabinoids Quantification and Identification in Cannabis Extracts. *Chemom. Intell. Lab. Syst.* **2023**, *232*, 104717, doi:10.1016/j.chemolab.2022.104717.
9. Todd, J.; Song, H.; Van Acker, R. Does Pollination Alter the Cannabinoid Composition and Yield of Extracts from Hemp (*Cannabis Sativa* L. Cv. Finola) Flowers? *Ind. Crop. Prod.* **2022**, *183*, 114989, doi:10.1016/j.indcrop.2022.114989.
10. Roussel, J.-M.; Schelling, C.; Righezza, M.; Veuthey, J.-L. Application of Prediction Intervals to the Interpretation of the Robustness Study of a UHPLC Method for the Separation of Cannabinoids. *J. Pharm. Biomed. Anal.* **2022**, *220*, 114977, doi:10.1016/j.jpba.2022.114977.
11. Lumu, S.; Hatterman-Valenti, H.; Hammed, A.; Monono, E. Effect of Short-Term Storage on Cannabinoid Content of Dried Floral Hemp (*Cannabis Sativa* L.) Inflorescence. *J. Appl. Res. Med. Aromat. Plants* **2024**, *42*, 100567, doi:10.1016/j.jarmap.2024.100567.
12. Malikova, L.; Malik, M.; Pavlik, J.; Ulman, M.; Pechouckova, E.; Skrivan, M.; Kokoska, L.; Tlustos, P. Anti-Staphylococcal Activity of Soilless Cultivated Cannabis across the Whole Vegetation Cycle under Various Nutritional Treatments in Relation to Cannabinoid Content. *Sci. Rep.* **2024**, *14*, 4343, doi:10.1038/s41598-024-54805-3.
13. Durante, C.; Anceschi, L.; Brighenti, V.; Caroli, C.; Afezolli, C.; Marchetti, A.; Cocchi, M.; Salamone, S.; Pollastro, F.; Pellati, F. Application of Experimental Design in HPLC Method Optimisation for the Simultaneous Determination of Multiple Bioactive Cannabinoids. *J. Pharm. Biomed. Anal.* **2022**, *221*, 115037, doi:10.1016/j.jpba.2022.115037.

14. Monari, A.; Cantalù, S.; Zanfrognini, B.; Brighenti, V.; Verri, P.; Zanardi, C.; Pellati, F.; Pigani, L. An Electrochemical Approach for the Prediction of Δ9-Tetrahydrocannabinolic Acid and Total Cannabinoid Content in *Cannabis Sativa* L. *Analyst* **2023**, *148*, 4688–4697, doi:10.1039/D3AN01090B.
15. Anceschi, L.; Codeluppi, A.; Brighenti, V.; Tassinari, R.; Taglioli, V.; Marchetti, L.; Roncati, L.; Alessandrini, A.; Corsi, L.; Pellati, F. Chemical Characterization of Non-psychoactive *Cannabis Sativa* L. Extracts, in Vitro Antiproliferative Activity and Induction of Apoptosis in Chronic Myelogenous Leukaemia Cancer Cells. *Phytother. Res.* **2022**, *36*, 914–927, doi:10.1002/ptr.7357.
16. Raslan-Jaramillo, J.J.; Ríos-Gajardo, G.A.; Avello, M.A.; de Diego, M.G. Determination of Cannabinoids in *Cannabis Sativa* Oil and Infused Ice Cream by LC-DAD Method. *J. AOAC Int.* **2024**, *107*, 140–145, doi:10.1093/jaoacint/qsad122.
17. Dreger, M.; Szalata, M.; Górska-Paukszta, M.; Mańkowska, G.; Oleszak, G.; Kwiatkowska, E.; Ożarowski, M. Content of Cannabinoids in Clonally Propagated Industrial Hemp. *J. Nat. Fibers* **2023**, *20*, 2245968, doi:10.1080/15440478.2023.2245968.
18. Fućak, T.; Kreft, S.; Svedružić, Ž.M.; Tavčar, E. Mechanism and Kinetics of CBDA Decarboxylation into CBD in Hemp. *J. Plant Biochem. Biotech.* **2023**, *32*, 608–621, doi:10.1007/s13562-023-00847-z.
19. Kim, E.-S.; Park, S.-H.; Kinney, C.A.; Olejar, K.J.; Corredor-Perilla, I.C. Comparison of Decarboxylation Rates of Acidic Cannabinoids between Secretory Cavity Contents and Air-Dried Inflorescence Extracts in *Cannabis Sativa* Cv. 'Cherry Wine.' *Sci. Rep.* **2024**, *14*, 16411, doi:10.1038/s41598-024-66420-3.
20. Schadich, E.; Kaczorová, D.; Béres, T.; Džubák, P.; Hajdúch, M.; Tarkowski, P.; Ćavar Zeljković, S. Secondary Metabolite Profiles and anti-SARS-CoV-2 Activity of Ethanolic Extracts from Nine Genotypes of *Cannabis Sativa* L. *Arch. Pharm.* **2024**, *358*, e2400607, doi:10.1002/ardp.202400607.
21. Moreno-Chamba, B.; Salazar-Bermeo, J.; Hosseinian, F.; Martin-Bermudo, F.; Aguado, M.; De La Torre, R.; Martínez-Madrid, M.C.; Valero, M.; Martí, N.; Saura, D. Aromatic and Cannabinoid Profiles of Cannabis Inflorescences and Seed Oils: A Comprehensive Approach for Variety Characterization. *Ind. Crop. Prod.* **2024**, *210*, 118143, doi:10.1016/j.indcrop.2024.118143.
22. Sedan, D.; Vaccarini, C.; Demetrio, P.; Morante, M.; Montiel, R.; Saurí, A.; Andrinolo, D. Cannabinoid Content in Cannabis Flowers and Homemade Cannabis-Based Products Used for Therapeutic Purposes in Argentina. *Cannabis Cannabinoid Res.* **2023**, *8*, 197–206, doi:10.1089/can.2020.0117.
23. Kursa, W.; Jamiołkowska, A.; Wyrostek, J.; Kowalski, R. Attempts to Use Hemp (*Cannabis Sativa* L. Var. Sativa) Inflorescence Extract to Limit the Growth of Fungi Occurring in Agricultural Crops. *Appl. Sci.* **2024**, *14*, 1680, doi:10.3390/app14041680.
24. Skala, T.; Kahánková, Z.; Tauchen, J.; Janatová, A.; Kloucek, P.; Hubka, V.; Franková, A. Medical Cannabis Dimethyl Ether, Ethanol and Butane Extracts Inhibit the in Vitro Growth of Bacteria and Dermatophytes Causing Common Skin Diseases. *Front. Microbiol.* **2022**, *13*, 953092, doi:10.3389/fmicb.2022.953092.
25. Luca, S.V.; Wojtanowski, K.; Korona-Głowniak, I.; Skalicka-Woźniak, K.; Minceva, M.; Trifan, A. Spent Material Extractives from Hemp Hydrodistillation as an Underexplored Source of Antimicrobial Cannabinoids. *Antibiotics* **2024**, *13*, 485, doi:10.3390/antibiotics13060485.
26. Wilson, W.B.; Urbas, A.A.; Jensen, H.; Sander, L.C. High-Throughput LC-PDA Method for Determination of Δ9-THC and Related Cannabinoids in *Cannabis Sativa*. *Forensic Sci.* **2024**, *41*, 100610, doi:10.1016/j.forc.2024.100610.
27. Glinn, M.A.; Michaud, G.P. Potency Levels of Regulated Cannabis Products in Michigan 2021–2022. *J. Forensic Sci.* **2023**, *68*, 1894–1905, doi:10.1111/1556-4029.15345.

28. De Leersnijder, C.; Duchateau, C.; De Braekeleer, K.; Deconinck, E. Relative Response Factors and Multiple Regression Models in Liquid Chromatography to Quantify Low-Dosed Components Using Alternative Standards—Proof of Concept: Total Δ9-THC Content in Cannabis Flowers Using CBD as Reference. *Anal. Bioanal. Chem.* **2022**, *414*, 6507–6520, doi:10.1007/s00216-022-04208-y.
29. Tran, J.; Elkins, A.C.; Spangenberg, G.C.; Rochfort, S.J. High-Throughput Quantitation of Cannabinoids by Liquid Chromatography Triple-Quadrupole Mass Spectrometry. *Molecules* **2022**, *27*, 742, doi:10.3390/molecules27030742.
30. de Souza, M.R.; Koetz, M.; Limberger, R.P.; Henriques, A.T. DoE-Assisted Development and Validation of a Stability-Indicating HPLC-DAD Method for Simultaneous Determination of Five Cannabinoids in *Cannabis Sativa* L. Based on Analytical Quality by Design (AQbD) Concept. *Phytochem. Anal.* **2022**, *33*, 999–1017, doi:10.1002/pca.3154.
31. Spadafora, N.D.; Felletti, S.; Chenet, T.; Sirangelo, T.M.; Cescon, M.; Catani, M.; De Luca, C.; Stevanin, C.; Cavazzini, A.; Pasti, L. The Influence of Drying and Storage Conditions on the Volatilome and Cannabinoid Content of *Cannabis Sativa* L. Inflorescences. *Anal. Bioanal. Chem.* **2024**, *416*, 3797–3809, doi:10.1007/s00216-024-05321-w.
32. Chan, K.-W. Kick Starting Method Development for Hemp Analysis Using Cannabis as Equivalent Material. *Prob. Forensic Sci.* **2023**, 271–283, doi:10.4467/12307483PFS.22.015.17688.
33. MacLaughlin, L.L.; MacDonald, M.T. Is Nitrogen-Modified Atmosphere Packaging a Tool for Retention of Volatile Terpenes and Cannabinoids in Stored *Cannabis Sativa* Inflorescence? *J. Cannabis. Res.* **2024**, *6*, 42, doi:10.1186/s42238-024-00253-9.
34. Song, L.; LeBlanc, L.; Jovanovich, E.; Mohammad Al-Bataineh, A.; Jervelle Fabien, K. A Rapid and Accurate Liquid Chromatographic Method for Hemp Compliance Testing. *Forensic Chem.* **2024**, *40*, 100592, doi:10.1016/j.forc.2024.100592.
35. Song, L.; Provis, J.; Al-Bataineh, A.M.; Fabien, K.J.; Kotler, M. Development of a Liquid Chromatographic Method with a Different Selectivity for the Quantification of Eighteen Phytocannabinoids in Hemp. *Talanta Open* **2024**, *10*, 100336, doi:10.1016/j.talo.2024.100336.
36. Song, L.; Meyer, G.; Adejumo, E.; Jovanovich, E.; LeBlanc, L.; Provis, J. Potency Testing of up to Sixteen Cannabinoids in Hemp-Infused Edibles Using Liquid Chromatography Diode Array Detector with Optional Confirmation of Identity by Electrospray Ionization Time-of-Flight Mass Spectrometry. *Food Chem.* **2023**, *417*, 135819, doi:10.1016/j.foodchem.2023.135819.
37. Aviram, J.; Atzmony, D.; Frenklakh, A.; Kroll, A.; Zaks, I.; Hazekamp, A. THC Degradation Does Not Impair the Accuracy of THC Doses Aerosolized by the Metered-Dose SyqeAir Inhaler: A 24-Month Stability Trial. *J. Cannabis Res.* **2022**, *4*, 55, doi:10.1186/s42238-022-00166-5.
38. Monton, C.; Tanpao, T.; Navakul, C.; Pengkum, T.; Santasanasuwan, S.; Suksaeree, J.; Charoenchai, L.; Songsak, T. Cannabidiol, Δ9-Tetrahydrocannabinol, and Cannabinol Contents of *Cannabis Sativa* L. Inflorescences Claimed to Be Hang Kra Rog Phu Phan Cultivar Cultivated Outdoors in Various Locations of Thailand. *Phytochem. Lett.* **2023**, *57*, 126–132, doi:10.1016/j.phytol.2023.08.009.
39. Tzimas, P.S.; Petrakis, E.A.; Halabalaki, M.; Skaltsounis, L.A. Extraction Solvent Selection for *Cannabis Sativa* L. by Efficient Exploration of Cannabinoid Selectivity and Phytochemical Diversity. *Phytochem. Anal.* **2024**, *35*, 163–183, doi:10.1002/pca.3282.
40. Tzimas, P.S.; Beteinakis, S.; Petrakis, E.A.; Papastylianou, P.T.; Kakabouki, I.; Small-Howard, A.L.; Skaltsounis, L.A.; Halabalaki, M. Uncovering the Metabolite Complexity and Variability of Cultivated Hemp (*Cannabis Sativa* L.): A First Phytochemical Diversity Mapping in Greece. *Phytochemistry* **2024**, *222*, 114076, doi:10.1016/j.phytochem.2024.114076.

41. Acquavia, M.A.; Tesoro, C.; Pascale, R.; Ostuni, A.; Matera, I.; Bianco, G.; Scrano, L.; Bufo, S.A.; Ciriello, R.; Di Capua, A. Legal *Cannabis Sativa* L. Dried Inflorescences: Cannabinoids Content and Cytotoxic Activity against Human HepG2 Cell Line. *Appl. Sci.* **2023**, *13*, 4960, doi:10.3390/app13084960.
42. Fernández, S.; Castro, R.; López-Radcenco, A.; Rodriguez, P.; Carrera, I.; García-Carnelli, C.; Moyna, G. Beyond Cannabinoids: Application of NMR-Based Metabolomics for the Assessment of *Cannabis Sativa* L. Crop Health. *Front. Plant Sci.* **2023**, *14*, 1025932, doi:10.3389/fpls.2023.1025932.
43. Musetti, B.; Kun, A.; Menchaca, D.; Rodríguez-Haralambides, A.; Varela, J.; Thomson, L.; Bahnson, E.M. *Cannabis Sativa* Extracts Inhibit LDL Oxidation and the Formation of Foam Cells in Vitro, Acting as Potential Multi-Step Inhibitors of Atherosclerosis Development. *PLoS ONE* **2024**, *19*, e0310777, doi:10.1371/journal.pone.0310777.
44. Mostafaei Dehnavi, M.; Ebadi, A.; Peirovi, A.; Taylor, G.; Salami, S.A. THC and CBD Fingerprinting of an Elite Cannabis Collection from Iran: Quantifying Diversity to Underpin Future Cannabis Breeding. *Plants* **2022**, *11*, 129, doi:10.3390/plants11010129.
45. Silva Sofrás, F.M.; Alonso, R.; Retta, D.S.; Di Leo Lira, P.; Desimone, M.F.; van Baren, C.M. Development and Validation of a Simple, Fast, and Accessible HPLC-UV Method for Cannabinoids Determination in *Cannabis Sativa* L. Extracts and Medicinal Oils. *Curr. Pharm. Des.* **2023**, *29*, 1918–1928, doi:10.2174/1381612829666230809094304.
46. Gilmore, A.M.; Elhendawy, M.A.; Radwan, M.M.; Kidder, L.H.; Wanas, A.S.; Godfrey, M.; Hildreth, J.B.; Robinson, A.E.; ElSohly, M.A. Absorbance-Transmittance Excitation Emission Matrix Method for Quantification of Major Cannabinoids and Corresponding Acids: A Rapid Alternative to Chromatography for Rapid Chemotype Discrimination of *Cannabis Sativa* Varieties. *Cannabis Cannabinoid Res.* **2023**, *8*, 911–922, doi:10.1089/can.2021.0165.
47. Tajik, T.; Baghaei, K.; Moghadam, V.E.; Farrokhi, N.; Salami, S.A. Extracellular Vesicles of Cannabis with High CBD Content Induce Anticancer Signaling in Human Hepatocellular Carcinoma. *Biomed. Pharmacother.* **2022**, *152*, 113209, doi:10.1016/j.biopha.2022.113209.
48. Aguiar, F.C. dos S.P.; Rocha, E.D. Facing the Forensic Challenge of Cannabis Regulation: A Methodology for the Differentiation between Hemp and Marijuana Samples. *Braz. J. Anal. Chem.* **2022**, *9*, 162–176, doi:10.30744/brjac.2179-3425.AR-42-2021.
49. Bongiorno, P.; Lopalco, A.; Casiraghi, A.; Spennacchio, A.; Pitruzzella, A.; Lopedota, A.A.; Minghetti, P.; Denora, N. Digital Technologies Applied to Control the One-Step Process of Cannabis Olive Oil Preparations. *Pharmaceutics* **2023**, *15*, 870, doi:10.3390/pharmaceutics15030870.
50. Casiraghi, A.; Gentile, A.; Selmin, F.; Gennari, C.G.M.; Casagni, E.; Roda, G.; Pallotti, G.; Rovellini, P.; Minghetti, P. Ultrasound-Assisted Extraction of Cannabinoids from *Cannabis Sativa* for Medicinal Purpose. *Pharmaceutics* **2022**, *14*, 2718, doi:10.3390/pharmaceutics14122718.
51. Sip, S.; Stasiłowicz-Krzemień, A.; Sip, A.; Szulc, P.; Neumann, M.; Kryszak, A.; Cielecka-Piontek, J. Development of Delivery Systems with Prebiotic and Neuroprotective Potential of Industrial-Grade *Cannabis Sativa* L. *Molecules* **2024**, *29*, 3574, doi:10.3390/molecules29153574.
52. Jokić, S.; Jerković, I.; Pavić, V.; Aladić, K.; Molnar, M.; Kovač, M.J.; Vladimir-Knežević, S. Terpenes and Cannabinoids in Supercritical CO2 Extracts of Industrial Hemp Inflorescences: Optimization of Extraction, Antiradical and Antibacterial Activity. *Pharmaceuticals* **2022**, *15*, 1117, doi:10.3390/ph15091117.
53. Fernández, S.; Carreras, T.; Castro, R.; Perelmuter, K.; Giorgi, V.; Vila, A.; Rosales, A.; Pazos, M.; Moyna, G.; Carrera, I.; et al. A Comparative Study of Supercritical Fluid and Ethanol Extracts of Cannabis Inflorescences: Chemical Profile and Biological Activity. *J. Supercrit. Fluids* **2022**, *179*, 105385, doi:10.1016/j.supflu.2021.105385.

54. Qamar, S.; Torres, Y.J.M.; Parekh, H.S.; Falconer, J.R. Fractional Factorial Design Study for the Extraction of Cannabinoids from CBD-Dominant Cannabis Flowers by Supercritical Carbon Dioxide. *Processes* **2022**, *10*, 93, doi:10.3390/pr10010093.
55. Szalata, M.; Dreger, M.; Zielińska, A.; Banach, J. Simple Extraction of Cannabinoids from Female Inflorescences of Hemp (*Cannabis Sativa* L.). *Molecules* **2022**, *27*, 5868, doi:10.3390/molecules27185868.
56. Stasiłowicz-Krzemień, A.; Sip, S.; Szulc, P.; Walkowiak, J.; Cielecka-Piontek, J. The Antioxidant and Neuroprotective Potential of Leaves and Inflorescences Extracts of Selected Hemp Varieties Obtained with scCO2. *Antioxidants* **2023**, *12*, doi:10.3390/antiox12101827.
57. Malík, M.; Praus, L.; Tlustoš, P. Comparison of Recirculation and Drain-to-Waste Hydroponic Systems in Relation to Medical Cannabis (*Cannabis Sativa* L.) Plants. *Ind. Crop. Prod.* **2023**, *202*, 117059, doi:10.1016/j.indcrop.2023.117059.
58. Toloza, H.; Buitrago, O.Y.; Orjuela, A.; Santaella, M.A.; Hurtado, A.M.; Arturo, D.E. Solvent Extraction of *Cannabis Sativa* under Cryogenic Conditions. *Sep. Purif. Technol.* **2024**, *329*, 124906, doi:10.1016/j.seppur.2023.124906.
59. Abeysekera, S.K.; Robinson, A.; Ooi, M.P.-L.; Kuang, Y.C.; Manley-Harris, M.; Holmes, W.; Hirst, E.; Nowak, J.; Caddie, M.; Steinhorn, G.; et al. Sparse Reproducible Machine Learning for near Infrared Hyperspectral Imaging: Estimating the Tetrahydrocannabinolic Acid Concentration in *Cannabis Sativa* L. *Ind. Crop. Prod.* **2023**, *192*, 116137, doi:10.1016/j.indcrop.2022.116137.
60. Elhendawy, M.A.; Radwan, M.M.; Ibrahim, E.A.; Wanas, A.S.; Chandra, S.; Godfrey, M.; ElSohly, M.A. Validation and Quantitation of Fifteen Cannabinoids in Cannabis and Marketed Products Using High-Performance Liquid Chromatography-Ultraviolet/Photodiode Array Method. *Cannabis Cannabinoid Res.* **2024**, *9*, e1091–e1107, doi:10.1089/can.2022.0335.
61. Ghosh, D.; Chaudhary, N.; Shanker, K.; Kumar, B.; Kumar, N. Monoecious *Cannabis Sativa* L. Discloses the Organ-Specific Variation in Glandular Trichomes, Cannabinoids Content and Antioxidant Potential. *J. Appl. Res. Med. Aromat. Plants* **2023**, *35*, 100476, doi:10.1016/j.jarmap.2023.100476.
62. Mehdi, J.; Husain, D.; Srivastava, A.; Gupta, N.; Shanker, K.; Talha, M.; Lal, R.K.; Kumar, B.; Gupta, A.K. The Genetic Diversity and Chemical Profiling of Natural Populations of Indian Cannabis. *Ind. Crop. Prod.* **2023**, *203*, 117187, doi:10.1016/j.indcrop.2023.117187.
63. Judžentienė, A.; Garjonytė, R.; Būdienė, J. Phytochemical Composition and Antioxidant Activity of Various Extracts of Fibre Hemp (*Cannabis Sativa* L.) Cultivated in Lithuania. *Molecules* **2023**, *28*, 4928, doi:10.3390/molecules28134928.
64. Kim, J.; Kim, D.-G.; Kim, J.-B.; Ryu, J.; Lee, Y.-J.; Woo, N.M.; Sik, J.J.; Kim, S.H. Simple Method for Detecting Tetrahydrocannabinolic Acid and Tetrahydrocannabinol in Cannabis Tissues Using Urine-Based Drug Diagnostic Device. *Ind. Crop. Prod.* **2024**, *219*, 119007, doi:10.1016/j.indcrop.2024.119007.
65. Velechovský, J.; Malík, M.; Šenkyřík, J.B.; Tlustoš, P. Effect of Augmented Nutrient Composition and Fertigation System on Biomass Yield and Cannabinoid Content of Medicinal Cannabis (*Cannabis Sativa* L.) Cultivation. *Front. Plant Sci.* **2024**, *15*, 1322824, doi:10.3389/fpls.2024.1322824.
66. Park, S.-H.; Pauli, C.S.; Gostin, E.L.; Staples, S.K.; Seifried, D.; Kinney, C.; Vanden Heuvel, B.D. Effects of Short-Term Environmental Stresses on the Onset of Cannabinoid Production in Young Immature Flowers of Industrial Hemp (*Cannabis Sativa* L.). *J. Cannabis Res.* **2022**, *4*, 1–13, doi:10.1186/s42238-021-00111-y.
67. Hall, D.R.; Sinclair, J.S.; Bhuyan, D.J.; Khoo, C.; Li, C.G.; Sarris, J.; Low, M. Quality Control of Cannabis Inflorescence and Oil Products: Response Factors for the Cost-Efficient

Determination of Ten Cannabinoids by HPLC. *Talanta Open* **2022**, *5*, 100112, doi:10.1016/j.talo.2022.100112.
68. Wongumpornpinit, V.; Temkitthawon, P.; Paenkaew, S.; Saesong, T.; Waranuch, N.; Ingkaninan, K. Preparation of Cannabis Biomass and Crude Extracts with High Neutral Cannabinoid Content. *Sci. Technol. Asia* **2023**, *28*, 216227, doi:10.14456/SCITECHASIA.2023.40.
69. Mastellone, G.; Marengo, A.; Sgorbini, B.; Rubiolo, P.; Anderson, J.L.; Cagliero, C. Ultrasound-Assisted Dispersive Solid-Liquid Microextraction with Eutectic Solvents for the Determination of Cannabinoids in Different Hemp Products. *J. Chromatogr. B Analyt. Technol. Biomed. Life Sci.* **2024**, *1232*, 123967, doi:10.1016/j.jchromb.2023.123967.
70. Mileti, O.; Baldino, N.; Paleologo, M.F.O.; Lupi, F.R.; Marra, M.; Iacopetta, D.; Gabriele, D. Oil Extraction from Hemp Plant as a Potential Source of Cannabidiol for Healthy Protein Foods. *Antioxidants* **2023**, *12*, 1950, doi:10.3390/antiox12111950.
71. Cuchiaro, J.; Baumgartner, J.; Reynolds, M.M. Modeling a Pesticide Remediation Strategy for Preparative Liquid Chromatography Using High-Performance Liquid Chromatography. *J. Cannabis Res.* **2023**, *5*, 13, doi:10.1186/s42238-023-00172-1.
72. Mazzara, E.; Torresi, J.; Fico, G.; Papini, A.; Kulbaka, N.; Dall'Acqua, S.; Sut, S.; Garzoli, S.; Mustafa, A.M.; Cappellacci, L.; et al. A Comprehensive Phytochemical Analysis of Terpenes, Polyphenols and Cannabinoids, and Micromorphological Characterization of 9 Commercial Varieties of *Cannabis Sativa* L. *Plants* **2022**, *11*, 891, doi:10.3390/plants11070891.
73. Song, L.; Valenzuela, G.; Carlson, S.; Dodson, Z.; Adisa, M. Potency Testing of up to Twenty Cannabinoids by Liquid Chromatography Diode Array Detector with Optional Electrospray Ionization Time-of-Flight Mass Spectrometry. *Anal. Chim. Acta* **2022**, *1207*, 339827, doi:10.1016/j.aca.2022.339827.
74. Duchateau, C.; De Leersnijder, C.; Barhdadi, S.; Canfyn, M.; De Braekeleer, K.; Deconinck, E. Discrepancies between Validated GC-FID and UHPLC-DAD Methods for the Analysis of Δ9-THC and CBD in Dried Hemp Flowers. *Drug Test Anal.* **2022**, *14*, 1732–1743, doi:10.1002/dta.3354.
75. Benkirane, C.; Ben Moumen, A.; Fauconnier, M.-L.; Belhaj, K.; Abid, M.; Caid, H.S.; Elamrani, A.; Mansouri, F. Bioactive Compounds from Hemp (*Cannabis Sativa* L.) Seeds: Optimization of Phenolic Antioxidant Extraction Using Simplex Lattice Mixture Design and HPLC-DAD/ESI-MS(2) Analysis. *RSc Adv.* **2022**, *12*, 25764–25777, doi:10.1039/d2ra04081f.
76. Benkirane, C.; Mansouri, F.; Ben Moumen, A.; Taaifi, Y.; Melhaoui, R.; Caid, H.S.; Fauconnier, M.; Elamrani, A.; Abid, M. Phenolic Profiles of Non-industrial Hemp (*Cannabis Sativa* L.) Seed Varieties Collected from Four Different Moroccan Regions. *Int. J. of Food Sci. Tech.* **2023**, *58*, 1367–1381, doi:10.1111/ijfs.16298.
77. Czauderna, M.; Taubner, T.; Wojtak, W. Comparative Study of Gas and Liquid Chromatography Methods for the Determination of Underivatised Neutral and Acidic Cannabinoids and Cholesterol. *Molecules* **2024**, *29*, 2165, doi:10.3390/molecules29102165.
78. Fu, Y.; Zhu, S.; Duan, S.; Liu, L. Bioassay-Guided Isolation and Identification of Antibacterial Components against *Escherichia Coli* from Industrial Hemp Leaves. *Separations* **2023**, *10*, 35, doi:10.3390/separations10010035.
79. Luangpraditkun, K.; Pimjuk, P.; Phimnuan, P.; Wisanwattana, W.; Wisespongpand, C.; Waranuch, N.; Viyoch, J. Anti-Aging Properties of *Cannabis Sativa* Leaf Extract against UVA Irradiation. *Cosmetics* **2024**, *11*, 45, doi:10.3390/cosmetics11020045.
80. Stasiłowicz-Krzemień, A.; Sip, S.; Szulc, P.; Cielecka-Piontek, J. Determining Antioxidant Activity of Cannabis Leaves Extracts from Different Varieties—Unveiling Nature's Treasure Trove. *Antioxidants* **2023**, *12*, 1390, doi:10.3390/antiox12071390.
81. Erukainure, O.L.; Oyenihi, O.R.; Amaku, J.F.; Chukwuma, C.I.; Nde, A.L.; Salau, V.F.; Matsabisa, M.G. *Cannabis Sativa* L. Modulates Altered Metabolic Pathways Involved in

Key Metabolisms in Human Breast Cancer (MCF-7) Cells: A Metabolomics Study. *Heliyon* **2023**, *9*, e16156, doi:10.1016/j.heliyon.2023.e16156.
82. Nalli, Y.; Bashir Mir, K.; Amin, T.; Gannedi, V.; Jameel, E.; Goswami, A.; Ali, A. Divergent Synthesis of Fractionated *Cannabis Sativa* Extract Led to Multiple Cannabinoids C-&O-Glycosides with Anti-Proliferative/Anti-Metastatic Properties. *Bioorg. Chem.* **2024**, *143*, 107030, doi:10.1016/j.bioorg.2023.107030.
83. Mastellone, G.; Marengo, A.; Sgorbini, B.; Scaglia, F.; Capetti, F.; Gai, F.; Peiretti, P.G.; Rubiolo, P.; Cagliero, C. Characterization and Biological Activity of Fiber-Type *Cannabis Sativa* L. Aerial Parts at Different Growth Stages. *Plants* **2022**, *11*, 419, doi:10.3390/plants11030419.
84. Kim, A.L.; Yun, Y.J.; Choi, H.W.; Hong, C.-H.; Shim, H.J.; Lee, J.H.; Kim, Y.-C. Profiling Cannabinoid Contents and Expression Levels of Corresponding Biosynthetic Genes in Commercial Cannabis (*Cannabis Sativa* L.) Cultivars. *Plants* **2022**, *11*, 3088, doi:10.3390/plants11223088.
85. Boonyayothin, W.; Kobtrakul, K.; Khositanon, P.; Vimolmangkang, S.; Phoolcharoen, W. Development of a Plant-Produced Recombinant Monoclonal Antibody against Δ-9-Tetrahydrocannabinol (Δ9-THC) for Immunoassay Application. *Biotechnol. Rep.* **2022**, *34*, e00725, doi:10.1016/j.btre.2022.e00725.
86. Wilson, W.B.; Urbas, A.A.; Abdur-Rahman, M.; Romares, A.; Mistek-Morabito, E. Determination of Δ9-THC, THCA, Δ8-THC, and Total Δ9-THC in 53 Smokable Hemp Plant Products by Liquid Chromatography and Photodiode Array Detection. *Forensic Chem.* **2024**, *37*, 100550, doi:10.1016/j.forc.2024.100550.
87. Huang, S.; Qiu, R.; Fang, Z.; Min, K.; Van Beek, T.A.; Ma, M.; Chen, B.; Zuilhof, H.; Salentijn, G.Ij. Semiquantitative Screening of THC Analogues by Silica Gel TLC with an Ag(I) Retention Zone and Chromogenic Smartphone Detection. *Anal. Chem.* **2022**, *94*, 13710–13718, doi:10.1021/acs.analchem.2c01627.
88. Melchert, D.; Schaare, F.; Winterhalter, P.; Beuerle, T. CBD Products: Labeling Accuracy of an Obscure Niche Market. *Food Control* **2024**, *160*, 110375, doi:10.1016/j.foodcont.2024.110375.
89. Salazar-Bermeo, J.; Moreno-Chamba, B.; Martínez-Madrid, M.C.; Valero, M.; Rodrigo-García, J.; Hosseinian, F.; Martín-Bermudo, F.; Aguado, M.; De La Torre, R.; Martí, N.; et al. Preventing Mislabeling: A Comparative Chromatographic Analysis for Classifying Medical and Industrial Cannabis. *Molecules* **2023**, *28*, 3552, doi:10.3390/molecules28083552.
90. Jaidee, W.; Siridechakorn, I.; Nessopa, S.; Wisuitiprot, V.; Chaiwangrach, N.; Ingkaninan, K.; Waranuch, N. Kinetics of CBD, Δ^9 -THC Degradation and Cannabinol Formation in Cannabis Resin at Various Temperature and pH Conditions. *Cannabis Cannabinoid Res.* **2022**, *7*, 537–547, doi:10.1089/can.2021.0004.
91. Treyer, A.; Reinhardt, J.K.; Eigenmann, D.E.; Oufir, M.; Hamburger, M. Phytochemical Comparison of Medicinal Cannabis Extracts and Study of Their CYP-Mediated Interactions with Coumarinic Oral Anticoagulants. *Med. Cannabis Cannabinoids* **2023**, *6*, 21–31, doi:10.1159/000528465.
92. Tapia-Tapia, E.; Aránguiz, P.; Diaz, R.; Espinoza, L.; Weinstein-Oppenheimer, C.R.; Cuellar, M. Effect of Cannabis Sativa L. Extracts, Phytocannabinoids and Their Acetylated Derivates on the SHSY-5Y Neuroblastoma Cells' Viability and Caspases 3/7 Activation. *Biol. Res.* **2024**, *57*, 33, doi:10.1186/s40659-024-00506-0.
93. Lazarjani, M.; Seyfoddin, A.; Le, T.T.; Chen, T. Development and Validation of HPLC and GC Methods for Quantification of Cannabinoids and Terpenes Extracted by Ultrasound Assisted Extraction Technique. *Drug Anal. Res.* **2024**, *8*, 33–45, doi:10.22456/2527-2616.139666.

94. Mano-Sousa, B.J.; Alves, B.C.; Pedrosa, A.M.; Lima, P.L.; de Andrade, F.P.; Duarte-Almeida, J.M. Validation of Analytical Method of Cannabinoids: Novel Approach Using Turbo-Extraction. *Talanta* **2023**, *254*, 124108, doi:10.1016/j.talanta.2022.124108.
95. Correia, B.; Ahmad, S.M.; Quintas, A. Determination of Phytocannabinoids in Cannabis Samples by Ultrasound-Assisted Solid-Liquid Extraction and High-Performance Liquid Chromatography with Diode Array Detector Analysis. *J. Chromatogr. A* **2023**, *1705*, 464191, doi:10.1016/j.chroma.2023.464191.
96. Büttenbender, S.; Carlos, G.; Steppe, M.; Ortiz, R.S.; Limberger, R.P.; Mendez, A.S.L. Fast and Reliable Profiling of Cannabinoids in Seized Samples Using the Method of HPLC–DAD Followed by Chemometrics. *Forensic Toxicol.* **2022**, *40*, 407–413, doi:10.1007/s11419-022-00625-x.
97. Majumdar, C.G.; ElSohly, M.A.; Ibrahim, E.A.; Elhendawy, M.A.; Stanford, D.; Chandra, S.; Wanas, A.S.; Radwan, M.M. Effect of Gamma Irradiation on Cannabinoid, Terpene, and Moisture Content of Cannabis Biomass. *Molecules* **2023**, *28*, 7710, doi:10.3390/molecules28237710.
98. Mastellone, G.; Marengo, A.; Sgorbini, B.; Rubiolo, P.; Cagliero, C. Development of a Dispersive Solid-Liquid Microextraction Method Using Natural Eutectic Solvents for a Greener Extraction of Phytochemicals from Fiber-Type Cannabis Sp. *Ind. Crop. Prod.* **2022**, *187*, 115476, doi:10.1016/j.indcrop.2022.115476.
99. Muhammad Zen, N.A.; Kobtrakul, K.; Khositanon, P.; Sanookpan, K.; Buranasudja, V.; Vimolmangkang, S. Vegetable Oil-Based Cannabis: Its Cannabinoid Profiling and Photoprotective Effect on UVA-Irradiated Human Skin Keratinocytes. *Thai J. Pharm. Sci.* **2023**, *46*, 720–733, doi:10.56808/3027-7922.2658.
100. Coppey, F.; Schelling, C.; Veuthey, J.; Esseiva, P. Cloud-Enabled Handheld NIR Spectroscopy: A Transformative Approach for Real-Time Forensic Analysis of Cannabis Specimens. *Helv. Chim. Acta* **2023**, *106*, e202300052, doi:10.1002/hlca.202300052.
101. Darigh, F.; Iranbakhsh, A.; Oraghi Ardebili, Z.; Ebadi, M.; Hassanpour, H. Simulated Microgravity Contributed to Modification of Callogenesis Performance and Secondary Metabolite Production in *Cannabis Indica*. *Plant Physiol. Biochem.* **2022**, *186*, 157–168, doi:10.1016/j.plaphy.2022.07.012.
102. Piani, B.; Ferfuia, C.; Bortolomeazzi, R.; Verardo, G.; Baldini, M. Development and Optimization of an HPLC-PDA Method for the Determination of Major Cannabinoids in Hemp (*Cannabis Sativa* L.) Essential Oil Obtained by Hydrodistillation. *Food Anal. Methods* **2022**, *15*, 1677–1686, doi:10.1007/s12161-022-02229-y.
103. Kalinowska, M.; Płońska, A.; Trusiak, M.; Gołębiewska, E.; Gorlewska-Pietluszenko, A. Comparing the Extraction Methods, Chemical Composition, Phenolic Contents and Antioxidant Activity of Edible Oils from *Cannabis Sativa* and *Silybum Marianu* Seeds. *Sci. Rep.* **2022**, *12*, 20609, doi:10.1038/s41598-022-25030-7.
104. Mouton, M.; Gerber, M.; Van Der Kooy, F. Cure-All Cannabidiol? The Cannabidiol Content of Commercial Products. *Phytomed. Plus* **2024**, *4*, 100520, doi:10.1016/j.phyplu.2023.100520.
105. Putthong, C.; Temkitthawon, P.; Phimnuan, P.; Viyoch, J.; Tongpoolsomjit, K. Formulation of Chewable Nutraceuticals Prepared from Banana Fruit Pulp and Hemp Seed Oil. *Sci. Asia.* **2024**, *50*, 1, doi:10.2306/scienceasia1513-1874.2024.056.
106. Trovato, E.; Arena, K.; La Tella, R.; Rigano, F.; Laganà Vinci, R.; Dugo, P.; Mondello, L.; Guarnaccia, P. Hemp Seed-Based Food Products as Functional Foods: A Comprehensive Characterization of Secondary Metabolites Using Liquid and Gas Chromatography Methods. *J. Food Compos. Anal.* **2023**, *117*, 105151, doi:10.1016/j.jfca.2023.105151.
107. Li, H.; Zhao, Q.; Chang, S.; Wang, L.; Zhao, B. Phytochemical Analysis and Bioactivity of Different Ethanolic Extracts from Cannabidiol Full-Spectrum Oil. *J. Mol. Liq.* **2023**, *372*, 121173, doi:10.1016/j.molliq.2022.121173.

108. Ohtsuki, T.; Friesen, J.B.; Chen, S.-N.; McAlpine, J.B.; Pauli, G.F. Selective Preparation and High Dynamic-Range Analysis of Cannabinoids in “CBD Oil” and Other *Cannabis Sativa* Preparations. *J. Nat. Prod.* **2022**, *85*, 634–646, doi:10.1021/acs.jnatprod.1c00976.
109. Vida, R.G.; Strauss, L.V.; Bajtel, Á.; Kiss, T.; Csupor, D.; Fittler, A. Safety and Risks of CBD Oils Purchased Online: Unveiling Uncertain Quality and Vague Health Claims. *Front. Pharmacol.* **2023**, *14*, 1273540, doi:10.3389/fphar.2023.1273540.
110. Santos Álvarez, I.; Pérez Lloret, P.; González Soriano, J.M.; Pérez Moreno, M. An Approach to the Evaluation of the Potency of Cannabis Resin in Madrid: A Health Hazard? *Adicciones* **2023**, *35*, 279–288, doi:10.20882/adicciones.1630.
111. Chauhan, J.; Bastia, B.K.; Kohli, K.; Chaudhary, B.; Chikara, G.; Gupta, A.; Kumar, A. Phytocannabinoid Profile and Potency of Cannabis Resin (Hashish) of Northwest Himalayas of India. *J. Forensic Sci.* **2024**, *69*, 1918–1925, doi:10.1111/1556-4029.15583.
112. Boumrah, Y.; Kermouche, W.; Zarita, B.; Drardja, E.D.; Bouanani, S. Hashish Seizures in Algeria over the 2019–2020 Period: The Rise of Potent Hashish Hybrids. *J. Forensic Sci.* **2022**, *67*, 889–898, doi:10.1111/1556-4029.15001.
113. Monton, C.; Chankana, N.; Leelawat, S.; Suksaeree, J.; Songsak, T. Optimization of Supercritical Carbon Dioxide Fluid Extraction of Seized Cannabis and Self-Emulsifying Drug Delivery System for Enhancing the Dissolution of Cannabis Extract. *J. Supercrit. Fluids* **2022**, *179*, 105423, doi:10.1016/j.supflu.2021.105423.
114. Song, L.; Carlson, S.; Valenzuela, G.; Chao, M.; Pathipaka, S.B. Development of a Validated Method for Rapid Quantification of up to Sixteen Cannabinoids Using Ultra-High-Performance Liquid Chromatography Diode-Array Detector with Optional Electrospray Ionization Time-of-Flight Mass Spectrometry Detection. *J. Chromatogr. A* **2022**, *1670*, 462953, doi:10.1016/j.chroma.2022.462953.
115. Gardener, H.; Wallin, C.; Bowen, J. Heavy Metal and Phthalate Contamination and Labeling Integrity in a Large Sample of US Commercially Available Cannabidiol (CBD) Products. *Sci. Total Environ.* **2022**, *851*, 158110, doi:10.1016/j.scitotenv.2022.158110.
116. Quiñones, R.; Moreno, S.; Smythers, A.L.; Sullins, C.; Pijor, H.; Brown, G.; Trouten, A.; Richards-Waugh, L.L.; Siddig, A. Quantification of Cannabis in Infused Consumer Products and Their Residues on Skin. *ACS Pharmacol. Transl. Sci.* **2022**, *5*, 642–651.
117. Mouton, M.; Gerber, M.; Van der Kooy, F. Validation of a Fast LC-PDA Method for the Quantification of Cannabinoids in Commercial Tea Samples. *Rev. Bras. Farmacogn.* **2024**, *34*, 197–201.
118. Osiripun, V.; Labua, S. Effects of Thermal Processing on Cannabidiol Degradation in Cannabidiol-Infused Pomegranate Juice and Evaluation of Its Antioxidant Property. *JCST* **2023**, *13*, 107–117.
119. Rylands, M.; Kusza, D.A.; Hlabisa, N.; Gwampa, T.; Ravenscroft, N. Development of Physicochemical Methods for the Quantification of CBD in South African Cannabis-Based Consumer Goods. *S. Afr. J. Chem.* **2024**.
120. Tejada Rodriguez, E.; Vella Szijj, J.; Cachia, M.; Falzon, P.; Axisa, K.; Serracino-Inglott, A.; Azzopardi, L.M. An Efficient HPLC-UV Method for Determination of Tetrahydrocannabinol in Oil. *Asian J. Pharm. Clin. Res.* **2023**, doi:10.22159/ajpcr.2023.v16i3.47462.
121. Huang, S.; Van Beek, T.A.; Claassen, F.W.; Janssen, H.-G.; Ma, M.; Chen, B.; Zuilhof, H.; Salentijn, G.Ij. Comprehensive Cannabinoid Profiling of Acid-Treated CBD Samples and Δ8-THC-Infused Edibles. *Food Chem.* **2024**, *440*, 138187, doi:10.1016/j.foodchem.2023.138187.
122. Johnson, D.A.; Hogan, M.; Marriot, R.; Heaney, L.M.; Bailey, S.J.; Clifford, T.; James, L.J. A Comparison of Advertised versus Actual Cannabidiol (CBD) Content of Oils, Aqueous Tinctures, e-Liquids and Drinks Purchased in the UK. *J. Cannabis Res.* **2023**, *5*, 28, doi:10.1186/s42238-023-00183-y.

123. Namera, A.; Ota, S.; Tomioka, Y.; Saito, T.; Nagao, M. Facile Determination of Natural Cannabinoids in Cannabis Products Using a Conventional Fully Porous Particle Column and Isocratic High-Performance Liquid Chromatography with Diode-Array Detector. *Forensic Toxicol* **2022**, *40*, 417–421, doi:10.1007/s11419-022-00630-0.
124. Hajrulai-Musliu, Z.; Dimitreska Stojkovikj, E.; Gusheski, D.; Musliu, D.; Velkovski, D. High-Performance Liquid Chromatography with DAD Detection for the Determination of Cannabinoids in Commercial Veterinary CBD Oil. *Pharmacy* **2024**, *12*, doi:10.3390/pharmacy12060181.
125. Pathompak, P.; Chankana, N.; Vipunngeun, N.; Phonkrathok, S. HPLC Method Development and Validation for Quantitation of CBD and THC in Suk-SaiYas Pills. *J. Curr. Sci.* **2022**, *12*, 538546, doi:10.14456/JCST.2022.41.
126. Sumontri, S.; Eiamart, W.; Tadtong, S.; Samee, W. Ultra-High-Performance Liquid Chromatography–Tandem Mass Spectrometry Analysis of Δ9-Tetrahydrocannabinol and Cannabidiol in Commercial Suk-Saiyasna Herbal Remedy: Applying Hansen Solubility Parameters for Sample Extraction to Ensure Regulatory Compliance. *Pharmaceuticals* **2024**, *17*, 1502, doi:10.3390/ph17111502.
127. Pires, B.; Oliveira, P.; Simão, A.Y.; Reis, J.; Ramos, S.; Duarte, A.P.; Margalho, C.; Rosado, T.; Barroso, M.; Gallardo, E. Characterisation of Cannabis-Based Products Marketed for Medical and Non-Medical Use Purchased in Portugal. *Molecules* **2024**, *29*, 2737, doi:10.3390/molecules29122737.
128. Quiñones, R.; Casiano-Negroni, A.; Pijor, H.; Moreno, S.; Suarez, K.; Westfall, T.D.; Sullins, C.; Ivey, S.; Buxó, J.A. Analysis of Cannabinoids in Lotions Using High-Performance Liquid Chromatography. *J. Chem. Educ.* **2022**, *99*, 3558–3565, doi:10.1021/acs.jchemed.2c00214.
129. Chaiwangrach, N.; Waranuch, N.; Temkitthawon, P.; Wongwad, E.; Nuengchamnong, N.; Usuwanthim, K.; Saesong, T.; Rakkhetkorn, Y.; Pisutthanan, S.; Ingkaninan, K. Quality Assessment, Stability and in-Vitro Anti-Inflammatory Activities of a Topical Ointment Containing Cannabis and Turmeric Extracts for Haemorrhoids and Skin Diseases. *Phytomed. Plus* **2024**, *4*, 100635, doi:10.1016/j.phyplu.2024.100635.
130. Picco, A.; Segale, L.; Miletto, I.; Pollastro, F.; Aprile, S.; Locatelli, M.; Bari, E.; Torre, M.L.; Giovannelli, L. Spray-Dried Powder Containing Cannabigerol: A New Extemporaneous Emulgel for Topical Administration. *Pharmaceutics* **2023**, *15*, 2747, doi:10.3390/pharmaceutics15122747.
131. Tai, W.; Yau, G.T.Y.; Arnold, J.C.; Chan, H.-K.; Kwok, P.C.L. High-Loading Cannabidiol Powders for Inhalation. *Int. J. Pharm.* **2024**, *660*, 124370, doi:10.1016/j.ijpharm.2024.124370.
132. Tai, W.; Arnold, J.C.; Chan, H.-K.; Kwok, P.C.L. Spray Freeze Dried Cannabidiol with Dipalmitoylphosphatidylcholine (DPPC) for Inhalation and Solubility Enhancement. *Int. J. Pharm.* **2024**, *659*, 124235, doi:10.1016/j.ijpharm.2024.124235.
133. Analakkattillam, S.; Langsi, V.K.; Hanrahan, J.P.; Moore, E. Analytical Method Validation for Assay Determination of Cannabidiol and Tetrahydrocannabinol in Hemp Oil Infused Products by RP-HPLC. *Sci. Rep.* **2022**, *12*, 12453, doi:10.1038/s41598-022-13737-6.
134. Sucontphunt, A.; Lukman Sueree; Jongrungruangchok, S.; Maha, A.; Madaka, F.; Chankana, N.; Chaiyabutr, K.; Leelawat, S.; Songsak, T.; Pradubyat, N. Validation and Comparison of Method Using Different Wavelength Detection at 210 and 228 Nm for Quantitative Analysis of CBD in Sublingual Cannabis Oil Products. *Interprof. J. Health Sci.* **2022**, *20*, 28–35.
135. Zheng, H.; Chen, B.; Rao, J. Nutraceutical Potential of Industrial Hemp (Cannabis Sativa L.) Extracts: Physicochemical Stability and Bioaccessibility of Cannabidiol (CBD) Nanoemulsions. *Food Funct.* **2022**, *13*, 4502–4512, doi:10.1039/d1fo04433h.
136. Hameed al-maraseemi, B.; Usmani, Z.; rizwanullah, md; Amin, S.; Mir, S.R. Box-Behnken Design Assisted Development and Optimization of RP-HPLC-PDA Technique for

Determination of Cannabidiol in the Bulk and Nanoformulation. *Egypt. J. Chem.* **2022**, *65*, 1–2, doi:10.21608/ejchem.2021.82528.4072.
137. Khabir, Z.; Partalis, C.; Panchal, J.V.; Deva, A.; Khatri, A.; Garcia-Bennett, A. Enhanced Skin Penetration of Cannabidiol Using Organosilane Particles as Transdermal Delivery Vehicles. *Pharmaceutics* **2023**, *15*, doi:10.3390/pharmaceutics15030798.
138. Shreiber-Livne, I.; Sulimani, L.; Shapira, A.; Procaccia, S.; Meiri, D.; Sosnik, A. Poly (Ethylene Glycol)-b-Poly (Epsilon-Caprolactone) Nanoparticles as a Platform for the Improved Oral Delivery of Cannabidiol. *Drug Deliv. Transl. Res.* **2023**, *13*, 3192–3203.
139. Ediz, E.F.; Demirel Kars, M.; Çetin, K. Development and Characterization of New Cannabidiol-Loaded Polycaprolactone Nanoparticles for Increased Bioavailability. *ChemistrySelect* **2024**, *9*, e202403609, doi:10.1002/slct.202403609.
140. Lajoie, C.; Doyen, A.; Feutry, P.; Gagnon, D.; Brisson, G. Impact of Emulsifiers for the Nanoencapsulation with Maltodextrin of Cannabis Oil by Spray Drying on the Physicochemical Properties and Bioaccessibility of Cannabinoids. *Food Funct.* **2022**, *13*, 10320–10332, doi:10.1039/d2fo01591a.
141. Morakul, B.; Junyaprasert, V.B.; Sakchaisri, K.; Teeranachaideekul, V. Cannabidiol-Loaded Nanostructured Lipid Carriers (NLCs) for Dermal Delivery: Enhancement of Photostability, Cell Viability, and Anti-Inflammatory Activity. *Pharmaceutics* **2023**, *15*, 537, doi:10.3390/pharmaceutics15020537.
142. Klinsang, T.; Charoensit, P.; Phimnuan, P.; Luangpraditkun, K.; Ross, G.M.; Viennet, C.; Ross, S.; Viyoch, J. In Vitro Wound Healing Potential of a Fibroin Film Incorporating a Cannabidiol/2-Hydroxypropyl-β-Cyclodextrin Complex. *Pharmaceutics* **2023**, *15*, doi:10.3390/pharmaceutics15122682.
143. Barhdadi, S.; Courselle, P.; Deconinck, E.; Vanhee, C. The Analysis of Cannabinoids in E-Cigarette Liquids Using LC-HRAM-MS and LC-UV. *J. Pharm. Biomed. Anal.* **2023**, *230*, 115394, doi:10.1016/j.jpba.2023.115394.
144. Carona, A.; Bicker, J.; Fonseca, C.; da Graça Campos, M.; Falcão, A.; Fortuna, A. Development and Validation of an HPLC-DAD Method for the Quantification of Cannabigerol, Cannabidiol, Cannabinol and Cannabichromene in Human Plasma and Mouse Matrices. *Analyst* **2024**, *149*, 3815–3827, doi:10.1039/d4an00070f.
145. Babayeva, M.; Srdanovic, I. Non-Linear Plasma Protein Binding of Cannabidiol. *J. Cannabis Res.* **2024**, *6*, 27, doi:10.1186/s42238-024-00238-8.
146. Cheng, A.; Zhang, S.; Meng, F.; Xing, M.; Liu, H.; Yang, G.; Gao, Y. Nanosuspension-Loaded Dissolving Microneedle Patches for Enhanced Transdermal Delivery of a Highly Lipophilic Cannabidiol. *IJN* **2024**, *Volume 19*, 4061–4079, doi:10.2147/IJN.S452207.
147. Muresan, P.; McCrorie, P.; Smith, F.; Vasey, C.; Taresco, V.; Scurr, D.J.; Kern, S.; Smith, S.; Gershkovich, P.; Rahman, R. Development of Nanoparticle Loaded Microneedles for Drug Delivery to a Brain Tumour Resection Site. *Eur. J. Pharm. Biopharm.* **2023**, *182*, 53–61, doi:10.1016/j.ejpb.2022.11.016.
148. Kollipara, R.; Langille, E.; Tobin, C.; French, C.R. Phytocannabinoids Reduce Seizures in Larval Zebrafish and Affect Endocannabinoid Gene Expression. *Biomolecules* **2023**, *13*, 1398, doi:10.3390/biom13091398.
149. Patirathnan, N.; Tummarintra, P.; Phakdeenukoolkitja, S. Analysis of THC and CBD in Cannabis Commercial Oil: Preliminary Study. *KKU Research Journal (Graduate Studies)* **2024**, *24*, 143–154.
150. Genovese, S.; Epifano, F.; Palumbo, L.; Collevecchio, C.; Fiorito, S. An Easy Way for the Removal of Residual Hydrocarbon Fractions from Crystallized Cannabigerol and Cannabidiol. *Food Bioprod. Process.* **2023**, *142*, 50–58, doi:10.1016/j.fbp.2023.09.001.
151. Kalinowska, M.; Gryko, K.; Wróblewska, A.M.; Jabłońska-Trypuć, A.; Karpowicz, D. Phenolic Content, Chemical Composition and Anti-/pro-Oxidant Activity of Gold

Milenium and Papierowka Apple Peel Extracts. *Sci. Rep.* **2020**, *10*, 14951, doi:10.1038/s41598-020-71351-w.
152. Jarén, C.; Zambrana, P.C.; Pérez-Roncal, C.; López-Maestresalas, A.; Ábrego, A.; Arazuri, S. Potential of NIRS Technology for the Determination of Cannabinoid Content in Industrial Hemp (*Cannabis Sativa* L.). *Agronomy* **2022**, *12*, 938, doi:10.3390/agronomy12040938.
153. *Recommended Methods for the Identification and Analysis of Cannabis and Cannabis Products: Manual for Use by National Drug Analysis Laboratories*; United Nations Office on Drugs and Crime, Ed.; Rev. and updated.; United Nations: New York, 2009; ISBN 978-92-1-148242-3.
154. Analakkattillam, S.; Langsi, V.K.; Hanrahan, J.P.; Moore, E. Analytical Method Validation for Assay Determination of Cannabidiol and Tetrahydrocannabinol in Hemp Oil Infused Products by RP-HPLC. *Sci Rep* **2022**, *12*, 12453, doi:10.1038/s41598-022-13737-6.
155. Yu, C.; Long, R.; Cao, F.; Zhao, X.; Lan, T.; Xu, D. Development of Pure Certified Reference Material of Cannabidiol. *Molecules* **2024**, *29*, doi:10.3390/molecules29050921.
143. Vella Szijj, J.; Gallo, L.L.; Buhagiar, P.I.; Szyrner, K.; Sammut Bartolo, N.; Ronsisvalle, S.; Serracino Inglott, A.; Azzopardi, L.M. Analysis of Cannabinoids in Oil. *Integr. Med. Rep.* **2024**, *3*, 122–131, doi:10.1089/imr.2024.0019.
157. Brighenti, V.; Marani, M.; Caroli, C.; Bertarini, L.; Gaggiotti, A.; Pollastro, F.; Durante, C.; Cannazza, G.; Pellati, F. A New HPLC Method with Multiple Detection Systems for Impurity Analysis and Discrimination of Natural versus Synthetic Cannabidiol. *Anal. Bioanal. Chem.* **2024**, *416*, 4555–4569, doi:10.1007/s00216-024-05396-5.
158. Fries, A.; Moldes, C.A.; Mazzaferro, L.S. Cost-Efficient Analysis of Cannabinoids in Therapeutic Oils Using HPLC with UV and Mass Spectrometry Detection. *Nat. Prod. Res.* **2024**, 1–9, doi:10.1080/14786419.2024.2439024.
159. Gul, W.; Gul, S.W.; Radwan, M.M.; Wanas, A.S.; Mehmedic, Z.; Khan, I.I.; Sharaf, M.H.M.; ElSohly, M.A. Determination of 11 Cannabinoids in Biomass and Extracts of Different Varieties of Cannabis Using High-Performance Liquid Chromatography. *J. AOAC Int.* **2015**, *98*, 1523–1528, doi:10.5740/jaoacint.15-095.
160. Birenboim, M.; Fallik, E.; Kengisbuch, D.; Shimshoni, J.A. Quantitative and Qualitative Spectroscopic Parameters Determination of Major Cannabinoids. *J. Lumin.* **2022**, *252*, 119387, doi:10.1016/j.jlumin.2022.119387.
161. Peters, F.T.; Drummer, O.H.; Musshoff, F. Validation of New Methods. *Forensic Sci. Int.* **2007**, *165*, 216–224, doi:10.1016/j.forsciint.2006.05.021.
162. Pittiglio, M.K.; Ramirez, G.A.; Tesfatsion, T.T.; Ray, K.P.; Cruces, W. HPLC Method for Better Separation of THC Isomers to Ensure Safety and Compliance in the Hemp Market. *ACS Omega* **2024**, *9*, 25390–25394, doi:10.1021/acsomega.4c03897.
